# Integrated Molecular Characterization of Testicular Germ Cell Tumors

**DOI:** 10.1016/j.celrep.2018.05.039

**Published:** 2018-06-12

**Authors:** Hui Shen, Juliann Shih, Daniel P. Hollern, Linghua Wang, Reanne Bowlby, Satish K. Tickoo, Vésteinn Thorsson, Andrew J. Mungall, Yulia Newton, Apurva M. Hegde, Joshua Armenia, Francisco Sánchez-Vega, John Pluta, Louise C. Pyle, Rohit Mehra, Victor E. Reuter, Guilherme Godoy, Jeffrey Jones, Carl S. Shelley, Darren R. Feldman, Daniel O. Vidal, Davor Lessel, Tomislav Kulis, Flavio M. Cárcano, Kristen M. Leraas, Tara M. Lichtenberg, Denise Brooks, Andrew D. Cherniack, Juok Cho, David I. Heiman, Katayoon Kasaian, Minwei Liu, Michael S. Noble, Liu Xi, Hailei Zhang, Wanding Zhou, Jean C. ZenKlusen, Carolyn M. Hutter, Ina Felau, Jiashan Zhang, Nikolaus Schultz, Gad Getz, Matthew Meyerson, Joshua M. Stuart, The Cancer Genome Atlas Research Network, Rehan Akbani, David A. Wheeler, Peter W. Laird, Katherine L. Nathanson, Victoria K. Cortessis, Katherine A. Hoadley

**Affiliations:** 1 Van Andel Research Institute, Grand Rapids, MI 49503, USA; 2 The Eli and Edythe L. Broad Institute of Massachusetts Institute of Technology and Harvard University, Cambridge, MA 02142, USA; 3 Department of Medical Oncology, Dana-Farber Cancer Institute, Boston, MA 02215, USA; 4 Tufts University School of Medicine, 136 Harrison Avenue, Boston, MA 02111, USA; 5 Department of Genetics, Lineberger Comprehensive Cancer Center, University of North Carolina at Chapel Hill, Chapel Hill, NC 27599, USA; 6 Human Genome Sequencing Center, Baylor College of Medicine, Houston, TX 77030, USA; 7 Department of Genomic Medicine, Division of Cancer Medicine, The University of Texas MD Anderson Cancer Center, Houston, TX 77054, USA; 8 Canada’s Michael Smith Genome Sciences Centre, BC Cancer Agency, Vancouver, BC V5Z 4S6, Canada; 9 Department of Pathology, Memorial Sloan Kettering Cancer Center, New York, NY 10065, USA; 10 Institute for Systems Biology, Seattle, WA 98109, USA; 11 Department of Biomolecular Engineering and Center for Biomolecular Science and Engineering, University of California, Santa Cruz, Santa Cruz, CA 95064, USA; 12 Department of Bioinformatics and Computational Biology, The University of Texas MD Anderson Cancer Center, Houston, TX 77030, USA; 13 Center for Molecular Oncology, Memorial Sloan Kettering Cancer Center, 1275 York Avenue, New York, NY 10065, USA; 14 Division of Translational Medicine and Human Genetics, Department of Medicine, Perelman School of Medicine, University of Pennsylvania, Philadelphia, PA 19105, USA; 15 Division of Genetics and Metabolism, Department of Pediatrics, Children’s Hospital of Philadelphia, Philadelphia, PA 19104, USA; 16 University of Michigan Hospital and Health Systems, 2G332 UH, 1500 East Medical Center Drive, Ann Arbor, MI 48109, USA; 17 Scott Department of Urology, Baylor College of Medicine, Houston, TX 77030, USA; 18 University of Wisconsin School of Medicine and Public Health, Madison, WI 53726, USA; 19 Genitourinary Oncology Service, Department of Medicine, Memorial Sloan Kettering Cancer Center, New York, NY 10065, USA; 20 Molecular Oncology Research Center, Barretos Cancer Hospital, Rua Antenor Duarte Villela, 1331, Barretos, Sao Paolo-SP, 14784-400, Brazil; 21 Institute of Human Genetics, University of Ulm, 89081 Ulm, Germany; 22 Institute of Human Genetics, University Medical Center Hamburg-Eppendorf, 20246 Hamburg, Germany; 23 Department of Urology, University Hospital Center Zagreb, University of Zagreb School of Medicine, 10000 Zagreb, Croatia; 24 Department of Clinical Oncology, Barretos Cancer Hospital, Rua Antenor Duarte Villela, 1331, Barretos, Sao Paolo-SP, 14784-400, Brazil; 25 The Research Institute at Nationwide Children’s Hospital, Columbus, OH 43205, USA; 26 Computational Biology Center, Memorial Sloan Kettering Cancer Center, New York, NY 10065, USA; 27 National Cancer Institute, NIH, Bethesda, MD 20892, USA; 28 National Human Genome Research Institute, NIH, Bethesda, MD 20892, USA; 29 Massachusetts General Hospital Cancer Center and Department of Pathology, Massachusetts General Hospital and Harvard Medical School, Boston, MA 02114, USA; 30 Abramson Cancer Center, Perelman School of Medicine at the University of Pennsylvania, Philadelphia, PA 19104, USA; 31 Departments of Preventive Medicine and Obstetrics and Gynecology, Norris Comprehensive Cancer Center, Keck School of Medicine, University of Southern California, Los Angeles, CA 90033, USA

## Abstract

We studied 137 primary testicular germ cell tumors (TGCTs) using high-dimensional assays of genomic, epigenomic, transcriptomic, and proteomic features. These tumors exhibited high aneuploidy and a paucity of somatic mutations. Somatic mutation of only three genes achieved significance—KIT, KRAS, and NRAS—exclusively in samples with seminoma components. Integrated analyses identified distinct molecular patterns that characterized the major recognized histologic subtypes of TGCT: seminoma, embryonal carcinoma, yolk sac tumor, and teratoma. Striking differences in global DNA methylation and microRNA expression between histology subtypes highlight a likely role of epigenomic processes in determining histologic fates in TGCTs. We also identified a subset of pure seminomas defined by KIT mutations, increased immune infiltration, globally demethylated DNA, and decreased KRAS copy number. We report potential biomarkers for risk stratification, such as miRNA specifically expressed in teratoma, and others with molecular diagnostic potential, such as CpH (CpA/CpC/CpT) methylation identifying embryonal carcinomas.

## In Brief

Shen et al. identify molecular characteristics that classify testicular germ cell tumor types, including a separate subset of seminomas defined by *KIT* mutations. This provides a set of candidate biomarkers for risk stratification and potential therapeutic targeting.

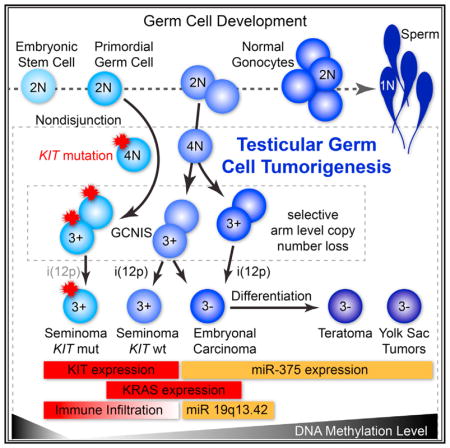

## INTRODUCTION

The most common malignancy of young adult males of European descent are testicular germ cell tumors (TGCTs) of the type derived from germ cell neoplasia *in situ* (GCNIS) (Moch et al., 2016). There are two major histologic types: pure classic seminoma and nonseminomatous germ cell tumors (NSGCTs). The latter, comprising embryonal carcinoma (EC), choriocarcinoma, yolk sac tumor, and teratoma, can contain a mix of both seminomatous and nonseminomatous components. Seminoma often has more indolent behavior, while NSGCT tends to occur at younger ages and confer higher mortality (Cortessis, 2003). TGCTs are now highly treatable, and overall relative survival of men with TGCTs exceeds 95% (Stang et al., 2013). However, survivors can experience devastating late effects of treatment, and a pressing research goal is the discovery of rational means of risk stratification that could spare some patients unnecessary chemotherapy, radiation, and surgery.

GCNIS is postulated to arise from incompletely differentiated fetal germ cells (primordial germ cells [PGCs]), based on shared morphology and immunohistochemical expression (Jørgensen et al., 1995). Both TGCTs and GCNIS cells are typically aneuploid, with hypertriploid to subtetraploid karyotypes (Summersgill et al., 2001), but GCNIS rarely exhibits 12p gains, which are pathognomonic for TGCTs (Ottesen et al., 2003). A model of tumor evolution postulates that nondisjunction creates tetraploid precursor cells, followed by a gain of isochromosome 12p during the transition from GCNIS to malignant NSGCTs (Frigyesi et al., 2004). A shared biological basis of seminoma and NSGCTs is supported by karyotypic similarities, TGCT risk alleles (Litchfield et al., 2016; Wang et al., 2017), and a report that tumor histology is unassociated in men with two primary TGCTs after adjustment for age (Thomas et al., 2013). DNA exome sequencing of several small cohorts of TGCTs have identified few significantly mutated somatic genes, primarily *KIT* and *KRAS* (Cutcutache et al., 2015; Litchfield et al., 2015; Taylor-Weiner et al., 2016). Lack of DNA methylation at CpG islands as determined by microarrays has been observed in seminomas (Smiraglia et al., 2002), and a global lack of methylated cytosines by immunohistochemistry staining has been described for GCNIS but not NSGCTs (Netto et al., 2008). Here, we characterize 137 TGCTs by DNA exome sequencing, RNA and microRNA (miRNA) sequencing, DNA SNP arrays, DNA methylation arrays, and reverse phase protein arrays.

## RESULTS

### Histologic Classification

**Figure 1:**
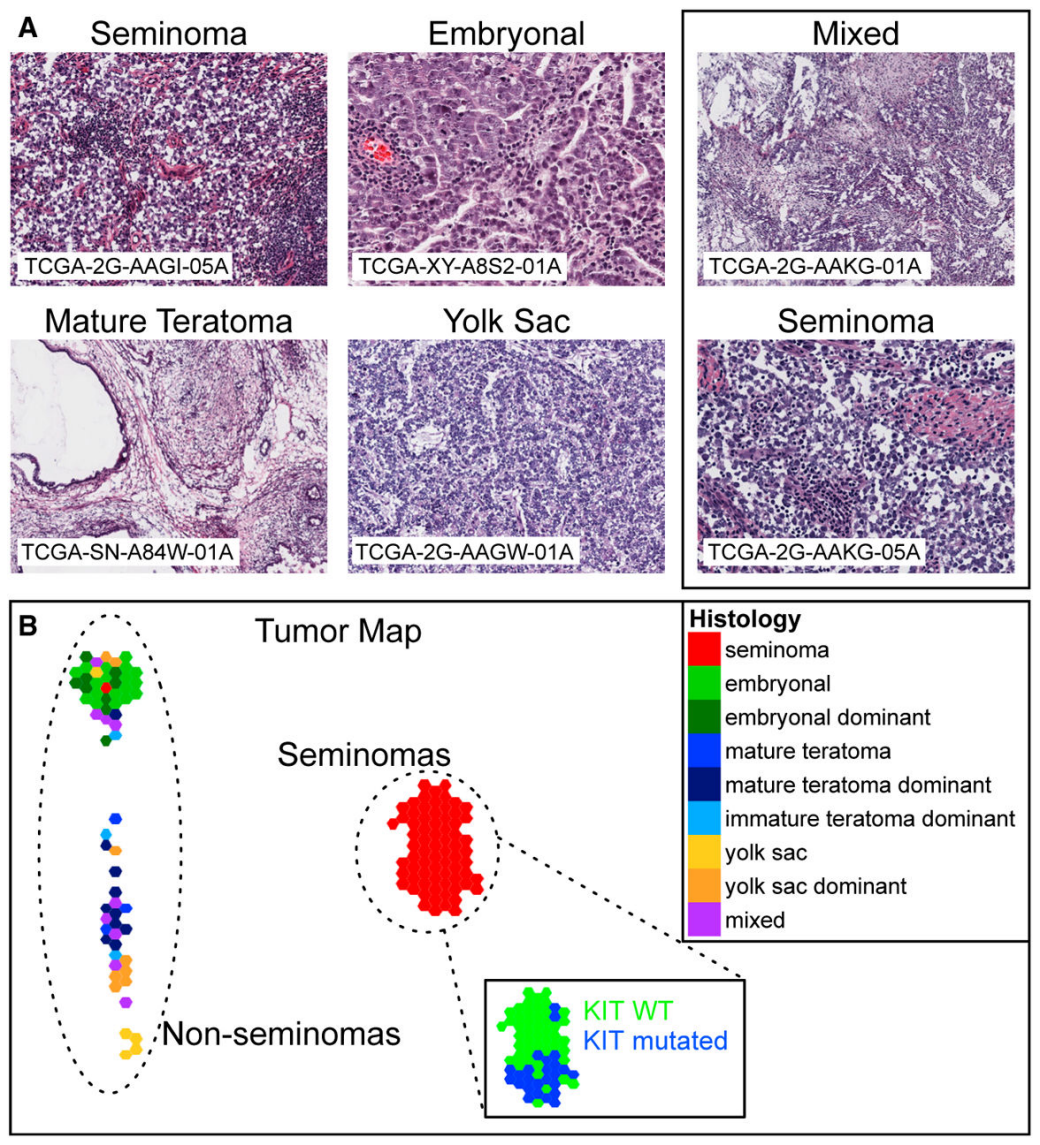
Histologic and Molecular Classification of TGCTs (A) Representative images of H&E-stained slides of frozen sections are shown for seminomas, EC, mature teratomas, and yolk sac tumors. Box at right shows two asynchronous primaries from the same patient. All images 100× magnification. (B) Tumor Map visual representation of molecular heterogeneity separating seminomas and NSGCTs. Samples are displayed as hexagons, and the spatial layout reflects sample groupings and molecular relations between samples. Samples are colored based on their histological classification. In the seminoma inset, samples are colored by KIT mutation status. *KIT* wild-type, green; *KIT* mutant, blue. See also Figures S1 and S5 and Tables S4, S5, S6, and S7.

Four pathologists reviewed fresh-frozen sections immediately adjacent to the tissue used for molecular analysis to confirm TGCT histology (Figure 1A). A consensus diagnosis was determined when at least three of four pathologists agreed on the tumor components and their percentage (within 10%) in the tissue block. Frozen sections of less than ideal quality were re-evaluated along with formalin-fixed, paraffin-embedded tissue sections to arrive at a final diagnosis. We used the final consensus histology from our pathology review for all of the analyses.

Samples were classified as “pure” for 100% and “dominant” for >60% presence of a given histology. The set of 137 tumors consisted of 72 seminoma, 18 EC, 9 EC dominant, 3 mature teratoma, 10 mature teratoma dominant, 3 immature teratoma dominant, 5 yolk sac tumor, 8 yolk sac tumor dominant, and 9 mixed tumors with no dominant component (Table S1). Two-class analyses compared pure seminoma (n = 72) with NSGCTs (n = 65). For patient-level analyses, we used the histology of the first or the only primary tumor (Table S2).

### Sample Characteristics

We studied 137 primary TGCTs from 133 patients, including 2 tumors from 4 patients with metachronous diagnoses. NSGCTs tended to be diagnosed at younger ages than were seminomas (median 30 versus 34 years, t test p value = 0.02). A personal history of cryptorchidism was more common among men diagnosed as having seminoma (17 of 68) rather than NSGCTs (5 of 65, χ^2^ p value = 0.008), but prevalence of a positive family history did not differ between these groups (χ^2^ p value = 0.3). Clinical characteristics were consistent with prior reports (Table S2).

### Unsupervised Classification of TGCTs

Unsupervised clustering analyses were performed to stratify tumor samples by each molecular platform (Figure S1). Seminomas were clearly distinguished from NSGCTs by DNA methylation, mRNA, miRNA, and protein. DNA copy number also distinguished seminoma from NSGCTs, although less completely.

We used Tumor Map to integrate mRNA expression, somatic copy number, and DNA methylation to visualize and spatially project relations among the samples (Newton et al., 2017). The resulting Tumor Map view (Figure 1B) completely distinguishes seminomas from NSGCTs in the molecular space, with EC-containing tumors positioned further apart from other NSGCTs. *KIT* mutation status further separates seminomas into two groups. The strong discrimination of histological types by unsupervised analysis leads us to focus subsequent analyses using the histological classification.

### DNA Sequence and Content

**Figure 2:**
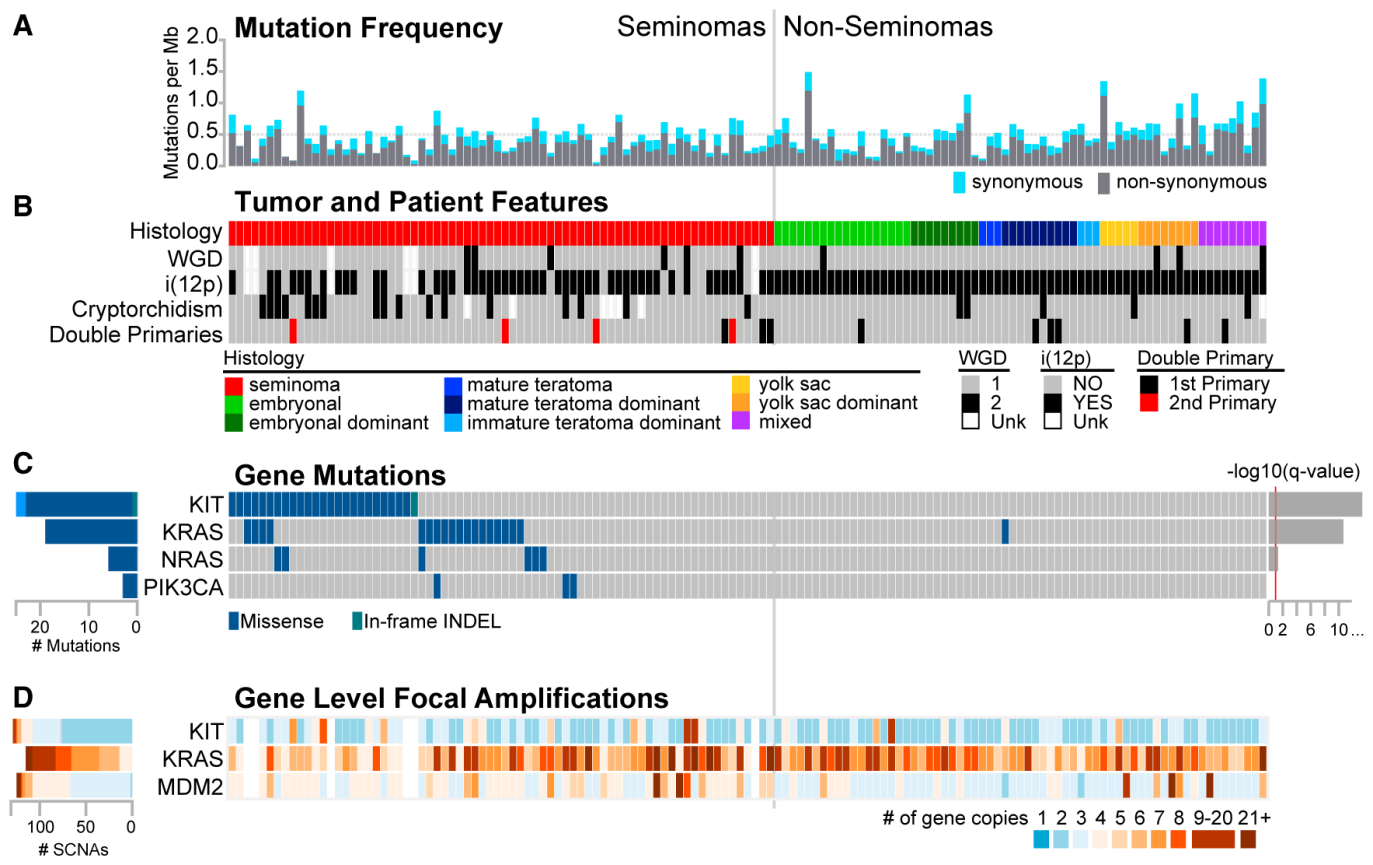
Molecular Alterations and Features across 137 TGCT Samples (A) Somatic mutation frequency (mutations/Mb) from exome sequencing. The horizontal gray dashed line marks the median mutation rate of 0.5 mutations/Mb. The vertical gray line divides pure seminomas from NSGCTs. (B) Tumor and patient features per sample. Whole genome doubling (WGD) and i(12p) status are using the ABSOLUTE algorithm. Calls for WGD or inferred i(12p) status could not be made for six low-purity samples. Cryptorchidism status, family history of testicular germ cell tumor (TGCT) or other cancer, and presence of double primaries are displayed. Unk, unknown. (C) Significant recurrent mutations (*KIT*, *KRAS*, and *NRAS*) or curated based on frequency or biological relevance. (D) Three known oncogenes were significantly focally amplified. Values represent the number of gene copies detected using the ABSOLUTE integer copy number. See also Figures S2 and S3 and Table S3.

Somatic mutation frequency varied by histology (Figure S2A). Overall median frequency, 0.5 mutations/Mb of targeted DNA (Figure 2A), was higher than that reported in pediatric tumors, but lower than most adult tumors (Lawrence et al., 2014) studied in The Cancer Genome Atlas (TCGA) (Figure S2B). The frequency of nonsynonymous mutations, 0.3 mutations/Mb, was similar to estimates from other TGCT exome-sequencing efforts (Cutcutache et al., 2015; Litchfield et al., 2015; Taylor-Weiner et al., 2016).

The most frequent type of mutation was the cytosine to thymine (C > T) transition, accounting for 40% of mutations (Table S1). Using mutational signature analysis as described by Covington et al. (2016), levels of C > T transition at CpG dinucleotides was significantly lower in seminoma with somatic *KIT* mutations than in either seminoma with wild-type *KIT* or NSGCTs (p = 0.002; Figure S2C). This signature, which correlates with Catalogue of Somatic Mutations in Cancer (COSMIC) mutation signature 1, is observed in most human tumors and postulated to result from the accumulation of 5-methylcytosine deamination events (Alexandrov et al., 2013).

Three genes were significantly somatically mutated: *KIT* (18%), *KRAS* (14%), and *NRAS* (4%) (Figure 2C), all described previously in TGCTs (Litchfield et al., 2015; Tian et al., 1999). These genes were exclusive to seminomas except for one *KRAS* mutation in an NSGCT with 30% seminoma. The *KIT* mutations were located in the activation loop of the KIT protein tyrosine kinase 2 (n = 19), the juxtamembrane domain (n = 6), and the protein tyrosine kinase 1 domain (n = 1), resembling those previously described in TGCTs (Litchfield et al., 2015) and intracranial germ cell tumors (Wang et al., 2014) (Figure S2D). *RAS* mutations clustered at known mutation hotspots (Figure S2D) and mutations in *KRAS* and *NRAS* co-existed in only one seminoma (Figure 2C). These mutations were particularly prevalent in seminomas diagnosed in men with a history of cryptorchidism (13 of 17). Of the six seminomas with mutations in both *KIT* and *KRAS*/*NRAS*, four were in men with a history of cryptorchidism (odds ratio = 7.3 [95% confidence interval 1.2–45.0]), all in the ipsilateral testicle. The PI3-kinase pathway influences germ cell proliferation in a Kit/Kit ligand-dependent fashion (Cardoso et al., 2014). Of note, three seminomas contained *PIK3CA* mutations, two at E545K and one at N345K (Figure S2D). Somatic *PIK3CA* mutations have been reported previously in two platinum-resistant TGCTs (Feldman et al., 2014). Only five other recurrently mutated genes were observed in our cohort, most with likely non-pathogenic mutations.

### SCNAs

All TGCTs had ploidy exceeding two, but NSGCTs demonstrated significantly lower ploidy than seminomas (median 2.8 versus 3.1, p = 1.3 × 10^−8^, Mann-Whitney *U* test) with variability across histology types (Figure S3A). Increased chromosomal content above a ploidy of two suggests that whole-genome duplication (WGD) occurred in all of the samples, and ten samples had evidence of two WGD events (Figure 2B), which is consistent with the proposed model of WGD followed by the deletion of chromosome arms (Frigyesi et al., 2004). Chromosome arm loss after WGD was specific to histological subtypes. NSGCTs had fewer copies of chromosomes (Chr) 19q, 15, 22, 19p, 10q, 8p, 2q, and 8q, whereas seminomas had fewer copies of 11q (Figure S1E). Even for arm-level somatic copy number alterations (SCNAs) shared between histologies, the timing of alterations differed between seminomas and NSGCTs, as inferred from the frequency of each event and the level of aneuploidy (Table S3; Figure S3C). For example, the deletion of Chr 4 was inferred to be an early event and 1p to be moderately early in all of the samples, whereas the deletion of 11q was inferred to be early only in seminomas and the deletion of Chr 15 to be early only in NSGCTs. We could not assess the copy number for six samples because of the low tumor purity.

We observed allelic copy number profiles consistent with the presence of at least one isochromosome 12p (i[12p]) in 114 of 131 (87%) tumors. All 17 tumors inferred lacking the i(12p) event were seminomas (Figure 2B) and retained at least 4 copies of 12p (Figure S1E). Only 2 of 131 samples exhibited loss of 12q heterozygosity, suggesting that most tumors had undergone a second WGD or a Chr 12 duplication event before i(12p) formation, as previously described (Geurts van Kessel et al., 1989).

We observed significantly recurrent focal amplifications of *KIT*, *KRAS*, and *MDM2* (Figures 2D and S3D)(McIntyre et al., 2004, 2005; Mostert et al., 2000). These amplifications contained entire genes and occurred with similar frequency in seminomas and NSGCTs. Seminomas with increased copies of *KRAS* (Chr 12) were more likely to have wild-type *KIT* (Figure 2D; t test p = 0.0007). Significantly reccurring focal deletions in chromosomal fragile sites *GRID2/ATOH1*, *JARID2*, *WWOX*, *NEGR1*, *PDE4D*, and *PARK2* occurred almost exclusively in NSGCTs and were shorter than the genes that they affected (Figure S3D).

### Inferred Order of Major Genetic Alterations

**Figure 3:**
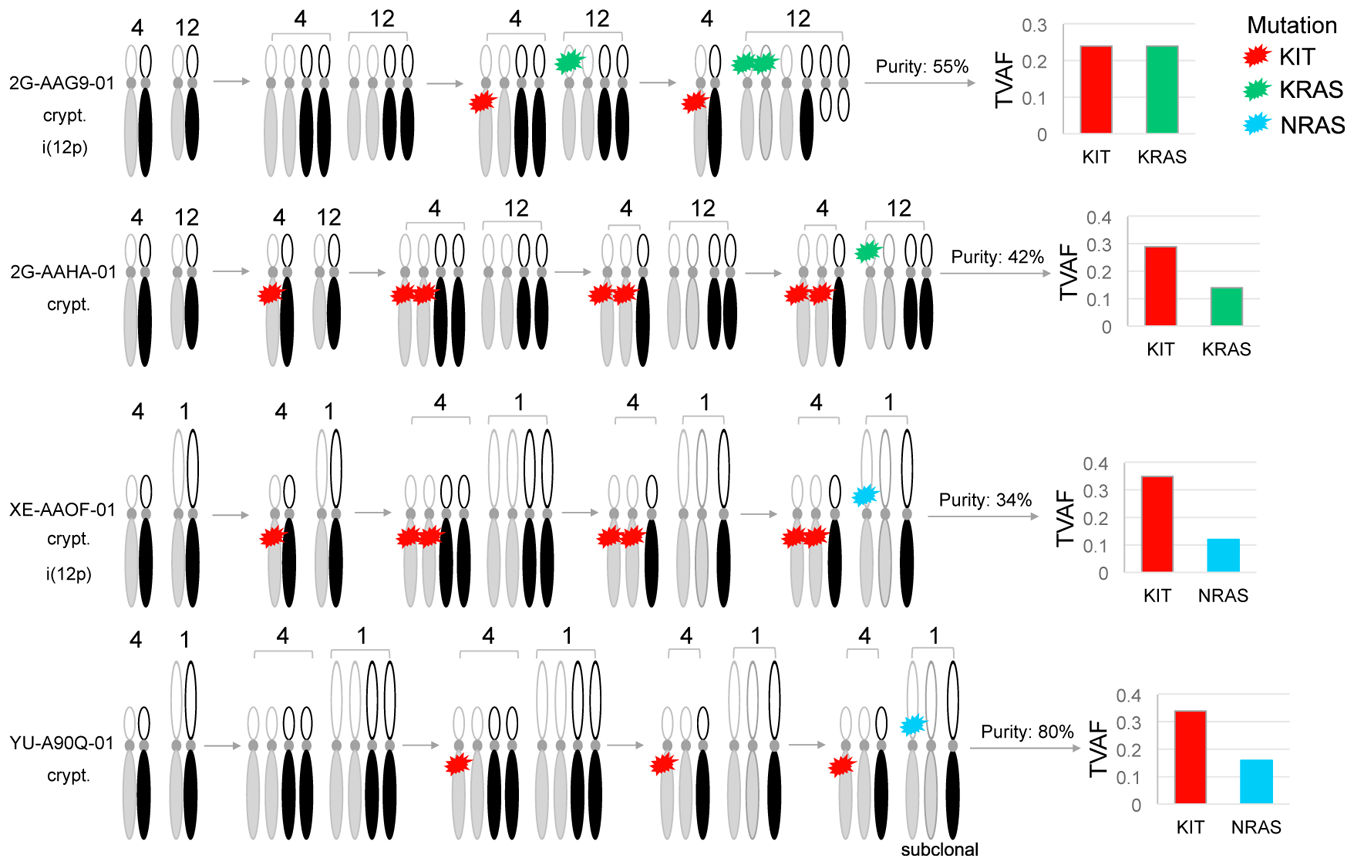
Inferred Order of Somatic Mutations and DNA Copy Number Alterations in TGCTs Four seminomas with co-existing somatic mutations in *KIT*, *KRAS*, and *NRAS* were selected. The timing of somatic events within each sample was inferred by integrated analysis of mutation multiplicity, allelic integer copy number, and whole-genome doubling status. Mutation multiplicity (s_q_) was calculated from purity, total copy number (CN), and tumor variant allele fraction (TVAF) as follows: s_q_ = TVAF[(CN*purity)+(2*(1–purity))]/purity. Integer copy number, whole-genome doubling status, and purity of tumor genomes were calculated using the ABSOLUTE algorithm. Cryptorchidism (crypt), isochromosome 12p [i(12p)]. Gray and black identify homologous chromosomes.

We inferred the relative order of alterations in tumors with mutations and sufficient tumor purity for estimating copy number. We used the variant allele fraction, allelic integer copy number, WGD, and purity estimates to calculate the mutation multiplicity, an inferred measurement of the number of alleles with a mutation. Four examples with mutations in both *KIT* and *KRAS/NRAS* are illustrated in Figure 3. Somatic *KIT* mutations were inferred to occur before WGD in two samples. *KIT* mutant multiplicities of an additional eight seminomas present a similar pattern, with variant allele fractions from DNA and RNA indicating a clonal nature. Genetic activation of *KIT* may arise early in TGCT tumorigenesis. In contrast, *RAS* mutations were inferred to be later events, all occurring after WGD. With the *KRAS* locus on 12p, we were able to infer the relative order between *KRAS* mutations and inferred i(12p) formation for 10 samples. Six samples had low mutation multiplicities, suggesting that they arose after i(12p) on either allele, while four samples had increased mutation multiplicities, suggesting that mutations arose before or during i(12p) formation. The other samples lacked i(12p) (n = 3), had low purity (n = 2), or we were unable to infer the order of events (n = 4). The number of wild-type (WT) *KRAS* copies was correlated with expression, but the number of mutant *KRAS* copies was not (Figure S3E).

### DNA Methylation

**Figure 4:**
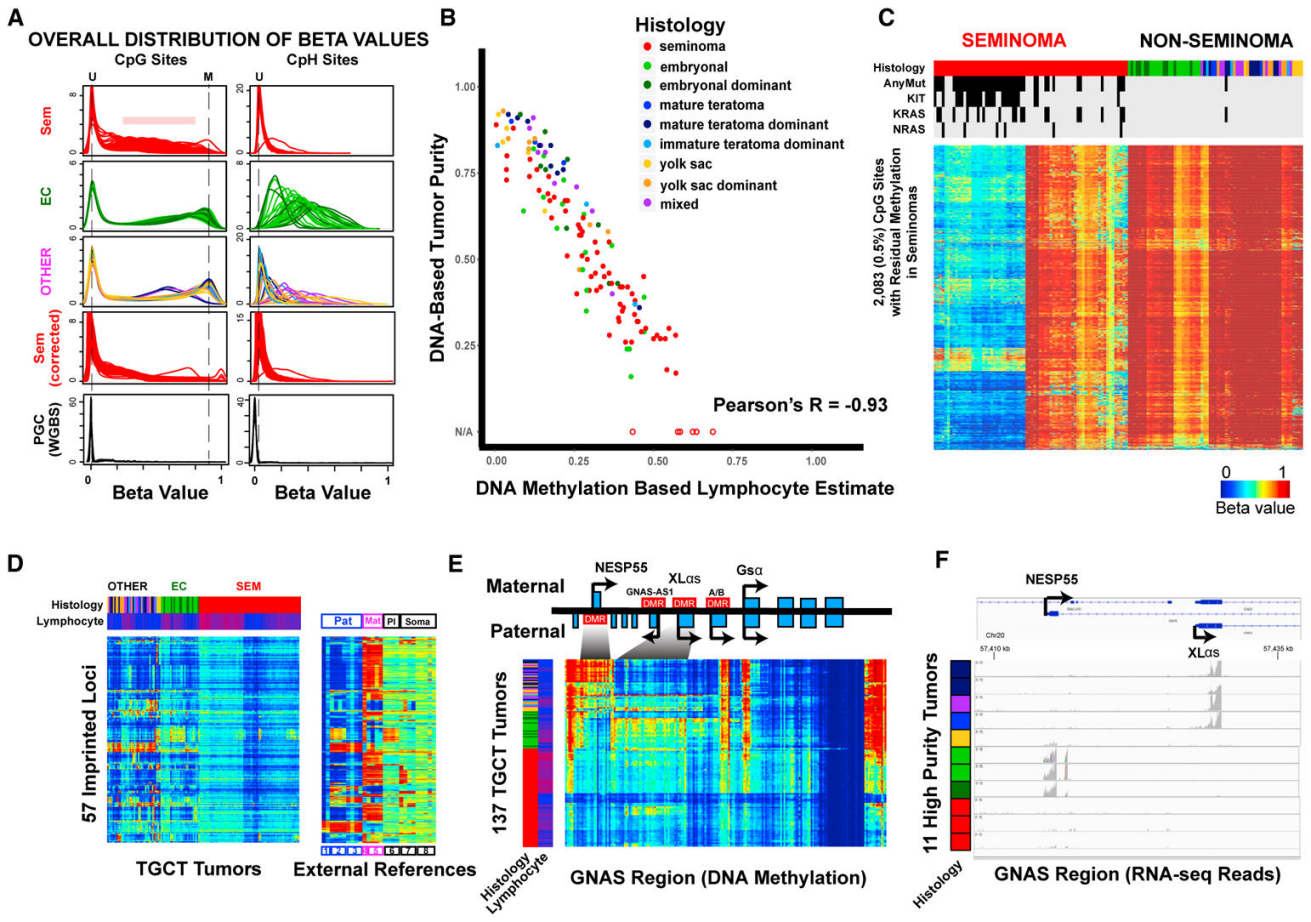
Dramatic DNA Methylation Differences Observed between TGCT Histology Types (A) Smoothed density plots show overall distributions of beta values at CpG (left) and CpH sites (right) grouped by seminoma, EC and EC dominant, and other (OTHER) tumors. Vertical dashed lines indicate locations corresponding to unmethylated (U) and methylated (M) sites. Four primordial germ cell (PGC) samples from external whole-genome bisulfite sequencing (WGBS) studies are plotted for the same sites included on the HM450 arrays. (B) Overall correlation between the DNA methylation signature-based lymphocyte estimates (x axis) versus mutation and SNP array-based (ABSOLUTE) tumor purity estimates (y axis) for 131 tumors. Six additional tumors without ABSOLUTE estimates as a result of extremely low purity are plotted with hollow circles. (C) DNA methylation at 2,083 (0.5%) loci (rows) with residual methylation in seminomas (columns) differs based on *KIT/RAS* mutation status. Data are corrected for lymphocyte infiltration (uncorrected data shown in Figure S1D). Blue to red indicates 0% to 100% methylation. Top color bars annotate the histology of each tumor and mutation status in *KIT/KRAS/NRAS* (black, mutants; gray, wild-type). (D) DNA methylation patterns at 57 imprinted loci. Inferred lymphocyte fraction is included as the second column color bar (blue to red: low to high level of contamination). External reference data (right) are plotted for the same set of loci representing paternal (Pat; 1, sperm; 2, hydatidiform mole; 3, paternal Unipaternal Disomy [pUPD] leukocyte), maternal (Mat; 4, maternal Unipaternal Disomy [mUPD] leukocyte; 5, parthenogenetically derived oocytes), and placental (Pl; 6, placenta)-imprinting patterns, in addition to ESCs (7, ESCs from 2, oocyte) and somatic tissues (8, somatic tissues). (E) The *GNAS* complex locus demonstrates contrasting DNA methylation patterns in different subtypes. Seminomas show an overall lack of methylation (observed methylation explainable by lymphocytic infiltration); EC and EC-dominant tumors show extensive methylation at the paternal DMR at the NESP55 promoter, and other tumors tend to have methylation at the maternal DMR near the XLαs promoter. (F) RNA-seq reads for different *GNAS* transcripts are consistent with DNA methylation patterns. Eleven tumors with relatively high purity of different histologies are shown. See also Figure S4.

Histological subtypes exhibited dramatically different global DNA methylation patterns. DNA methylation level as methylation fraction at a single locus is measured by the beta value, ranging from 0 to 1. For NSGCTs, the overall distribution of beta values at canonical CpG sites (Figure 4A) followed the bimodal pattern that is characteristic of most primary human tissue samples, with peaks for unmethylated and methylated CpGs. However, the methylated peak was not observed in seminomas, which instead demonstrated intermediate DNA methylation peaks in addition to the unmethylated peak, suggesting that seminoma samples contained two major cell types, one completely unmethylated and the other with full methylation at a subset of the loci. Using cell-type DNA methylation signatures, we identified infiltrating lymphocytes as the contaminant (Figure S4A), which is consistent with prior reports of extensive lymphocytic infiltration in seminomas (Hvarness et al., 2013; Parker et al., 2002). We estimated the percentage of lymphocytes in each tumor using cell-specific DNA methylation patterns. We also estimated tumor purity with ABSOLUTE (Carter et al., 2012), using copy number and mutation data (Figure S3B). A near-perfect anti-correlation (R = −0.93, p < 0.0001; Figure 4B) was observed between estimated lymphocyte fraction and tumor purity, validating both methods. Subtraction of lymphocyte DNA methylation contribution from all tumors led to the disappearance of intermediate methylation peaks in seminomas (Figure 4A), whereas the methylated peak remained in NSGCTs (Figure S4B). Consistent with a PGC origin for seminoma, the corrected density plot shows the majority of CpGs to be completely unmethylated in seminomas, similar to public PGC data (Figure 4A).

EC exhibited extensive methylation at non-canonical cytosine sites (e.g., CpA, CpT, CpC), collectively termed CpH sites (Figure 4A). CpH methylation was observed in tumors with an EC component, which is highly correlated with the pathological quantification of EC content (R = 0.86; Figure S4E) and associated with a high mRNA level of *de novo* DNA methyltransferases, DNMT3A/3B (Figure S4G). CpH methylation was first described in embryonic stem cells (ESCs) (Lister et al., 2009). Analysis of external PGC data (Figure 4A) revealed a lack of CpH methylation, indicating that this epigenetic similarity between EC and ESCs is not shared with PGCs (Figure S4D).

Global methylation is low in seminomas, with recurrent methylation observed at only <1% of all of the sites included on the HM450 array (Figure 4C). After correction for lymphocytes, the remnant methylation is absent in a subgroup of seminomas that are highly enriched for *KIT/KRAS* mutations (Figures 4C and S4C; p < 0.0001), suggesting an essentially complete lack of DNA methylation in this subset.

We comprehensively surveyed imprinted loci identified on the HM450 platform (Court et al., 2014). In seminomas, the degree of observed DNA methylation at these imprinted loci was in general lower than the levels that are characteristic of the biparental imprinting of soma (~0.5) and consistent with lymphocytic infiltration (Figure 4D). This pattern is expected in seminomas free of methylation at imprinted loci but contaminated by lymphocytes. The methylated CpG signal at these imprinted sites disappeared after correction for lymphocyte methylation in seminomas (data not shown), confirming the general lack of methylation at these sites. DNA methylation at imprinted loci was largely erased in NSGCTs as well, although some NSGCTs exhibited methylation at certain imprinted loci (Figure 4D), notably the *GNAS* complex imprinted locus, for which paternally, maternally, and biallelically expressed transcripts have been reported. Non-EC NSGCTs tended to be methylated at the paternal differentially methylated region (DMR), while EC had intermediate to high methylation at the maternal DMR (Figure 4E). RNA sequencing (RNA-seq) data from this region confirmed alternative usage of the promoters (Figure 4F) consistent with the observed DNA methylation pattern.

We investigated whether the presence of global DNA methylation in NSGCTs was random or followed certain patterns by examining the distribution of methylation by chromatin states in H1 ESCs (Figure S4F). We observed that active promoters in H1 ESCs, usually CpG islands, are generally unmethylated, whereas heterochromatin regions are extensively methylated. Thus, if this methylation was re-established after the DNA methylation nadir of PGCs, it largely followed preset rules similar to those in normal development, despite a failure to correctly establish imprinting methylation. EC exhibited overall DNA methylation similar to H1 ESCs (Figure S4D). However, poised (bivalent) promoters, which are prone to cancer-specific gain of methylation (Widschwendter et al., 2007), exhibited gain of methylation in NSGCTs. These sites include tumor suppressors, for which epigenetic silencing could contribute to tumorigenesis.

We observed epigenetic silencing of important tumor suppressors, including *BRCA1* (Koul et al., 2002), *MGMT* (Martinelli et al., 2016), and *RASSF1A* (Honorio et al., 2003) exclusively in NSGCTs (Figure S4H). We found epigenetic silencing of *RAD51C* in 16 NSGCTs (Figure S4I). *BRCA1* and *RAD51C* both are involved in the homologous recombination (HR) DNA repair pathway. Epigenetic silencing of *RAD51C* has been described in ovarian cancer with *BRCA1* deficiency (The Cancer Genome Atlas Research Network, 2011) but not in TGCTs. A locus containing *RAD51C* has been associated with TGCT susceptibility (Chung et al., 2013), highlighting the potential importance of homologous repair deficiency in TGCTs. We also found epigenetic silencing in *DNAJC15/MCJ* (Figure S4H), which in breast and uterine cancer cells has been associated with drug resistance (Fernández-Cabezudo et al., 2016).

### Expression of miRNA, mRNA, and Protein in TGCTs

Profiles of miRNA, mRNA, and protein differed between seminomas and NSGCTs. We noted several associations and confirmed previously reported characteristics, such as high *KIT* gene and protein expression in seminoma (Tables S4, S6, and S7).

EC tumors were distinguished by the high expression of numerous miRNAs. Expression of the *miR-519* genomic cluster on 19q13.42 was 25- to 50-fold higher in EC than in seminoma and 300- to 600-fold higher than in other types of NSGCTs, but it was not associated with copy number gain (Figure S5A). The miRNAs in this cluster have been shown to also be expressed in ESCs (Wilson et al., 2009). These miRNAs are likely to negatively regulate the expression of mRNA in EC because many of their targets have lower expression in EC (Table S6).

The miRNAs *miR-371*, *miR-372*, and *miR-373* have been proposed as serum biomarkers for monitoring patients with TGCTs for active disease as a strategy to minimize systemic therapy and attendant late effects (Syring et al., 2015). Highest sensitivity and specificity were reported for *miR-371a-3p* (Dieckmann et al., 2017). We interrogated 30 other TCGA tumor types and found *miR-371a-3p* to be dramatically overexpressed in TGCTs (Figure S5B), specifically seminoma, EC, and mixed NSGCTs, but minimally expressed in teratomas (Figure S5C). Conversely, *miR-375* was highly expressed in teratomas, yolk sac tumors, and mixed tumors containing these elements, but not in seminoma or EC (Figure S5C). Using a random forest classification, we defined a ranked series of miRNAs that distinguish seminoma, EC, and other NSGCTs (Table S5).

Using Paradigm to infer the activity of proteins, complexes, and general processes based on copy number and gene expression data, we identified seven major pathway activity clusters (Figure S1G). Three clusters, including *KRAS* signaling and immune infiltration, showed enriched activity in seminomas. All NSGCTs had enriched pathway activity for Wnt and MYC signaling. Samples with teratoma components had high pathway activities for the mammalian target of rapamycin (mTOR) and myogenesis, which is consistent with their differentiated nature.

### Immune Infiltration in Seminomas

**Figure 5:**
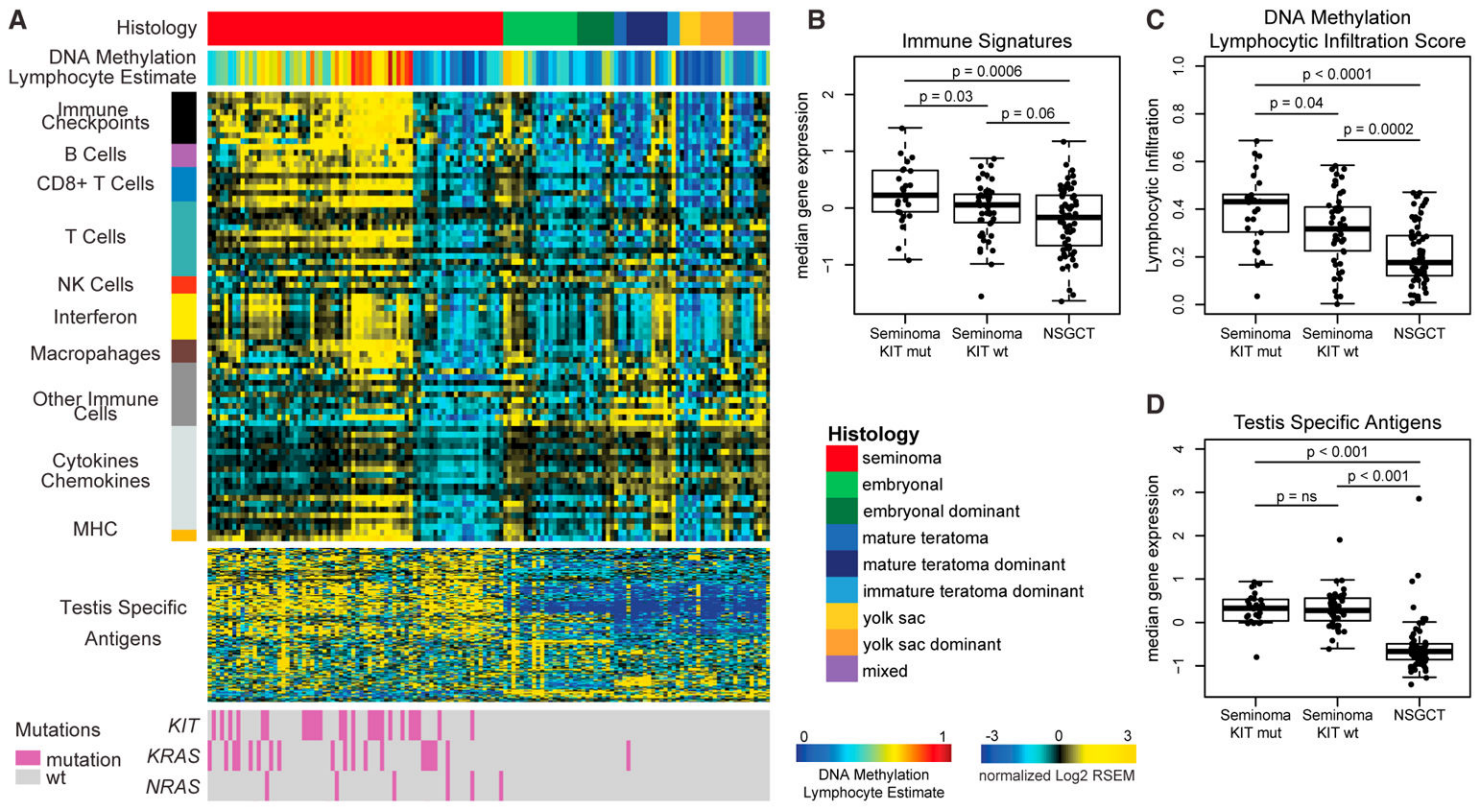
Immune Signatures Are High in Seminomas (A) Gene expression data (log2 median-centered RNA-seq by expectation maximization [RSEM] values) are displayed for 78 published gene expression signatures and ordered by immune category (left vertical bar). Tumors are ordered by histology and clustered by gene expression. Annotation tracks for DNA methylation lymphocyte infiltration score and mutation status are displayed. (B–D) Boxplots of immune features comparing seminoma *KIT* mutant, seminoma *KIT* WT, and NSGCTs. (B) Median expression of immune signatures, (C) DNA methylation lymphocytic infiltration scores, and (D) median cancer-testis-specific antigen gene expression. Boxplots display the median value, upper and lower quartiles, and the whiskers represent the interquartile range. Each dot represents the value of a single sample. See also Figure S6.

Extensive immune infiltration was noted in many of our seminoma samples during pathologic review and in the DNA methylation analysis (Figure S4A). Expression of 78 published immune gene expression signatures correlated with our DNA methylation-based lymphocyte content estimates (Figure 5A) and were highest in seminoma with *KIT* mutations compared to other samples (Figures 5B and 5C). The gene signatures suggest infiltration of several specific types of T cells (cytotoxic, CD8^+^, T central memory, T effector memory, and regulatory T cells), B cells, and activated dendritic cells.

We further analyzed T cell receptor (TCR; Figure S6A) and B cell receptor (BCR; Figure S6B) diversity across the sample set. Seminoma samples had both higher levels of TCRs and higher diversity of BCRs and TCRs. Examining total mutation load and predicted neoantigens, we identified high-affinity peptides but no specific antigen. Neither neoantigen signal nor total mutation load correlated with immune signatures (data not shown). We also did not find any association between immune cell signatures and either total or individual viral loads by mapping RNA-seq data to viral genomes (data not shown). Seminomas had higher expression of T effector memory cell signatures, suggesting the presence of antigen-experienced T cells. We interrogated mRNA levels of established cancer-testis-specific antigen (CTA) genes (Almeida et al., 2009) and noted higher levels in seminomas (Figure 5A), which may explain a polyclonal antigen-driven immune response around the tumor. However, although immune infiltration was increased in seminomas with *KIT* mutations, CTA genes expression levels did not differ by KIT status within seminomas (Figure 5D).

### KIT Pathway Alterations

**Figure 6:**
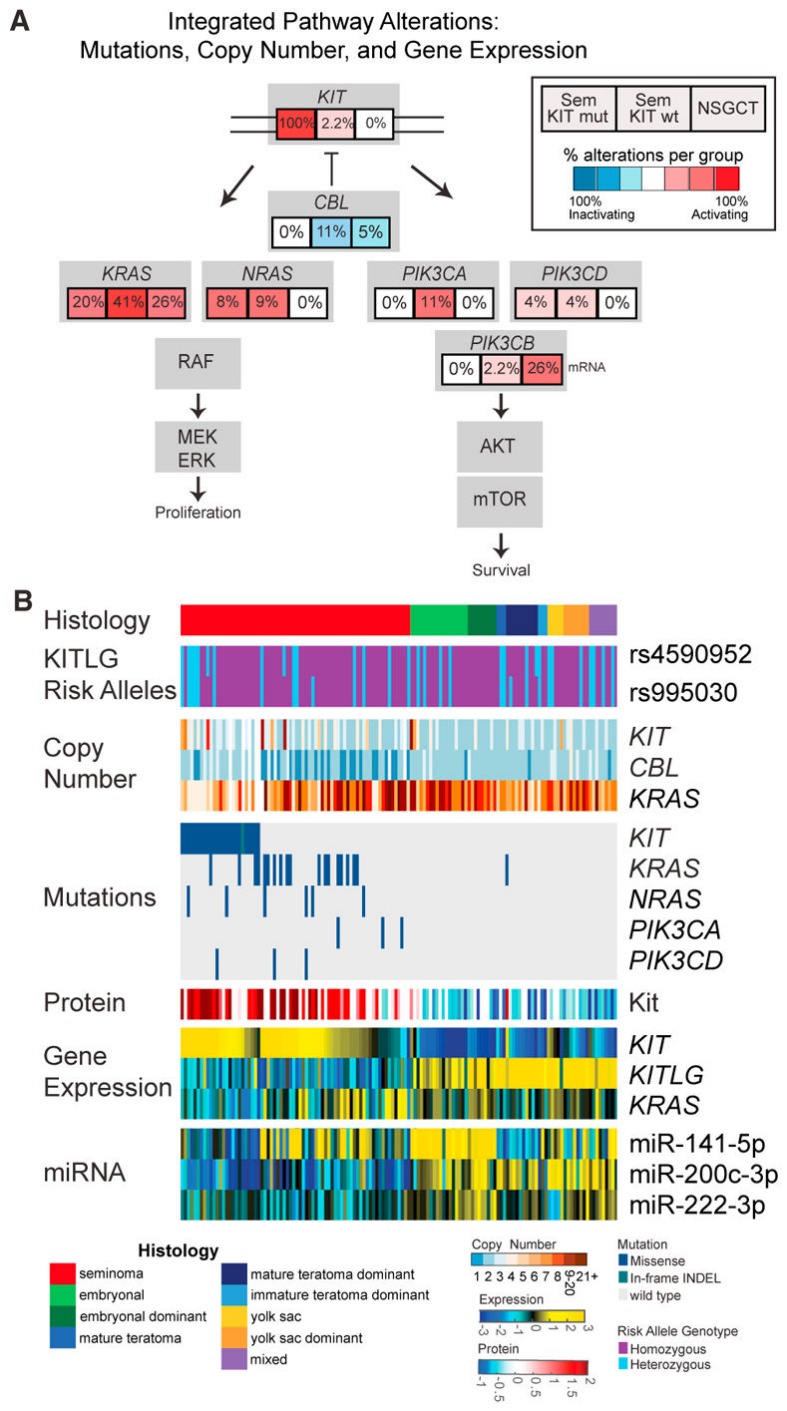
Analysis of *KIT* and *KIT* Ligand in TGCTs (A) Integrated analysis of the Kit pathway indicating the frequency of multiple genomic alterations within the Kit pathway. (B) A multiple platform characterization of *KIT* and *KIT* ligand across testicular germ cell tumors. Samples are first ordered by tumor histology. Within histology, tumors are ordered by *KIT* mutation status and then by *KIT* mRNA expression from high to low. Missing values are depicted as blank or white space within each heatmap. See also Figure S7.

Spermatogenesis requires coordinated germ cell proliferation and apoptosis, partly governed by KITLG-mediated KIT signaling via the PI3K pathway in mammals. All of the tumors had at least one risk allele as defined by each of two *KITLG* polymorphisms, which is consistent with prior germline data (Kanetsky et al., 2009). We calculated the percentage of alterations from mutations, copy number, and gene expression, and the KIT-PI3K pathway was the only enriched pathway, predominantly in seminomas (Figure 6A). This pathway includes five recurrently mutated genes: *KIT*, *KRAS*, *NRAS*, *PIK3CA*, and *PIK3CD*. Not only were *KIT* mutations enriched in seminomas but also *KIT* mRNA and protein were highly expressed in seminomas (Figure 6B). Within seminomas, *KIT* gene expression was higher in *KIT*-mutated tumors than in *KIT*-WT tumors, confirming the gain-of-function nature of these mutations (p = 0.001; Figure S7A). *KIT* focal amplifications were rare and did not, in general, amplify mutated copies of *KIT* (Table S1). Compared to NSGCTs, even seminomas without a *KIT* mutation or *KIT* focal amplification had higher expression of *KIT* mRNA and protein. *KIT* mutant seminomas had lower-level copy number levels and gene expression of *KRAS* than either *KIT* WT seminomas or NSGCTs. Gene signatures downstream of KIT signaling such as AKT, PI3K, KRAS, and JAK/STAT were high in seminomas, regardless of *KIT* or *KRAS* mutation status.

*CBL*, which regulates ubiquitin-mediated degradation of KIT, was deleted in 48% of KIT-WT seminomas, leaving just one copy (Figure 6B). *CBL* copy number negatively correlated with KIT protein expression for most tumors; however, seminomas with *KIT* or *KRAS* mutations maintained high protein levels of KIT regardless of *CBL* copies, apparently escaping *CBL* regulation (Figure S7C). In NSGCTs, we also observed high expression of *miR-222-3p*, a validated miRNA regulator of KIT. In tumors expressing the miR, *KIT* gene expression levels were low (Spearman’s rho = −0.55; Figure S7B; Gits et al., 2013).

### Double Primary TGCTs

**Figure 7:**
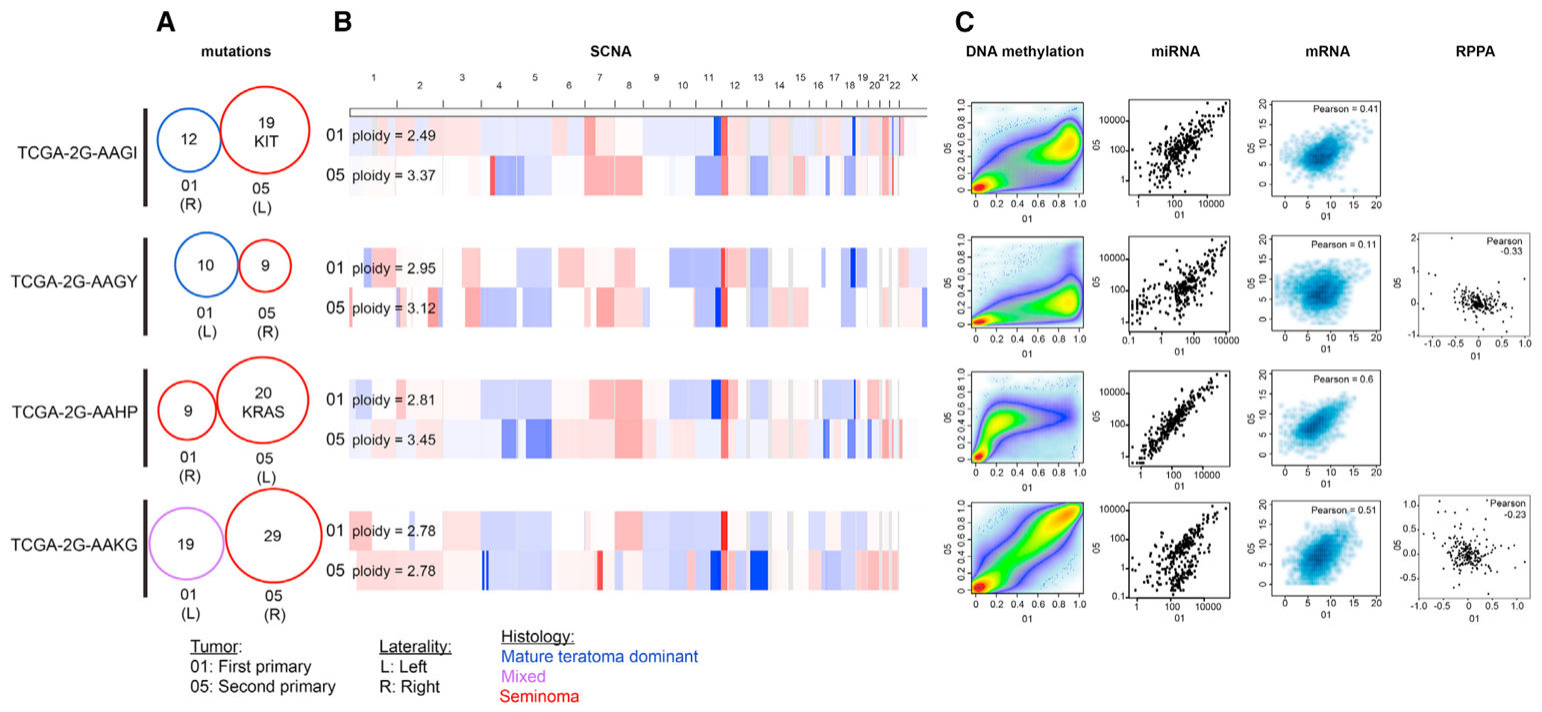
Genomic Alterations and Features in Four Patients with Asynchronous Double Primaries (A) Venn diagrams showing no overlap between somatic mutations identified in the first and second primary tumors, with number of significantly mutated genes shown. 01, first primary; 05, second primary; R, right; L, left. (B) SCNAs across the genome relative to the tumor ploidy (also shown). Red, amplification; blue, deletion. (C) For each platform, the first primary tumor is on the x axis and the second primary tumor is on the y axis. From left to right: beta values as a measure of DNA methylation across probes, with color representing the smoothed density of the probes; the reads per million (RPM) abundance of 303 miRNAs used in unsupervised clustering analysis on a log scale; log2 mRNA expression (RSEM) of 2,878 variably and highly expressed genes used for unsupervised clustering; RPPA expression values for 218 antibodies assayed. RPPA was not assessed for at least one primary tumor for patients TCGA-2G-AAGI and TCGA-2G-AAHP.

Approximately 2%–4% of men diagnosed as having TGCTs develop a second primary TGCT in the contralateral testicle (Fosså et al., 2005). We molecularly profiled both tumors from four men. One first primary was seminomas and three were NSGCTs; all second primaries were seminomas. The three histologically discordant pairs exhibited notably different profiles of all molecular features. Among all of the data types, the miRNA data were most highly correlated within all of the pairs (Figure 7). No mutation was shared between paired primaries (Figure 7A), as reported in three other pairs (Brabrand et al., 2015). Data from the two seminoma primaries of TCGA-2G-AAHP were the most similar across all of the platforms, even though this patient had received radiation between primaries. The apparent difference in ploidy and DNA methylation was the result of different amounts of lymphocytic contamination. Somatic mutation profiles yet again diverged between the two tumors, suggesting that genetic mutations are likely later events in these patients, and early copy number, epigenetic alterations, or both produce cells that are prone to transformation. The primaries from TCGA-2G-AAKG were divergent in histology, with the first tumor being a mixed tumor with 40% EC and the second being a pure seminoma. However, their DNA methylation profiles, both at CpG and CpH sites, were highly similar. The presence of CpH methylation in the first tumor is explained by the EC component, but CpH methylation in the second was unexpected, considering this sample had molecular and histologic appearances consistent with seminoma. This patient was the only one of the four with documented chemotherapy delivered between the two primary tumors.

## DISCUSSION

Integration of tumor characteristics and genomic and epigenomic data revealed distinctive molecular landscapes of TGCT histologic types and identified previously unappreciated diversity within seminomas (Table 1). All of the samples evinced WGD and a low mutation density. Only a few driver mutations were identified, exclusively in seminomas or samples with seminoma components. *KIT-*mutated seminomas separated from the KIT-WT seminomas on the Tumor Map and exhibited unique characteristics, including the highest levels of lymphocyte infiltration, the absence of global DNA methylation, reduced *KRAS* mutation frequency and copy number alterations, reduced frequency of estimated presence of inferred i(12p) events, and a more prevalent history of cryptorchidism (Table 1). Because *KIT* mutation was never observed in tumors lacking seminoma components, we postulate that this subset of seminomas is locked in a PGC-like status and remain pure seminomas, while those lacking *KIT* mutations may have the potential to differentiate into other histologies. We showed that cryptorchidism was enriched in seminomas, especially in men with *KIT*-mutated seminomas, shedding new light on established cryptorchidism-TGCT associations that warrant further investigation (Banks et al., 2013). All of the subtypes of NSGCTs shared genomic characteristics, including lower ploidy and higher purity than seminoma, and universal i(12)p. Recurrent somatic mutations were rarely present in NSGCTs, even though the overall mutation density was not dramatically different from seminomas (Table 1).

Previous studies noted both extensive lymphocytic infiltration and lack of DNA methylation in seminomas, features that we show for the first time to be more extreme in *KIT*-mutated seminomas. Signals from infiltrating cells influence genomic readout from the mixture of cellular components in the bulk tissue analyzed and need to be distinguished from tumor-specific signals. In our study, almost all of the DNA methylation signal in seminomas came from lymphocytes. Only by removing it were we able to reveal that seminomas lacked methylation genome wide and that those with *KIT* mutations had more complete lack of methylation. Although the recruitment and role of lymphocytes in TGCTs remain unclear, this immune response is likely multiclonal in nature because we did not observe clonal restriction of BCRs or TCRs. Demethylating agents were shown to elicit an immune reaction via “viral mimicry” caused by demethylation and consequent expression of endogenous retroviral elements (Chiappinelli et al., 2015; Roulois et al., 2015). Globally demethylated genomes of *KIT*-mutated seminoma cells could provoke a similar immune response. In line with this hypothesis, overexpression of human endogenous retroviral loci was reported in several seminoma samples (Gimenez et al., 2010). Global demethylation in *KIT-*mutated seminomas also may explain their significantly lower COSMIC mutational signature 1, because 5-methylcytosine, which occurs primarily in the CpG context, is 10 times more likely to mutate than a regular cytosine (C → T in the CpG context, explaining the majority of mutations that are observed in human cancers) (Shen and Laird, 2013).

TGCT models acknowledge that seminomas most closely resemble PGCs and GCNIS based on histologic appearance, gene expression, and lower levels of DNA methylation. GCNIS or seminomas are proposed precursors of EC, which is in turn the proposed precursor for extraembryonic (yolk sac tumor and choriocarcinoma) and somatic (teratoma) lineages (Honecker et al., 2006). We postulate that only seminomas without *KIT* mutations may be capable of acquiring nonseminomatous histology because all NSGCTs, including mixed TGCTs with seminoma components, lacked *KIT* mutations. Activating *KIT* mutations may lock KIT mutant seminoma cells into a PGC-like state in which *UHRF1* and *DNMT1* expression are suppressed, preventing the development of NSGCT components, which appear to require DNA methylation capacity. This may explain why seminomas have proven difficult to propagate *in vitro*, because DNA methylation at certain sites is essential for the survival of cancer cell lines (De Carvalho et al., 2012). Analysis of an external dataset (GSE60787) shows that TCAM-2, the sole seminoma cell line derived to date, has substantial DNA methylation.

Likely starting from an unmethylated precursor (PGC/GCNIS), NGSCTs re-establish methylation patterns corresponding to their cellular phenotypes: the epigenetic profile of EC resembles that of ESC, with extensive non-canonical CpH methylation (Lister et al., 2009), while non-EC NSGSTs adopt DNA methylation patterns resembling soma and extraembryonal lineages. Frequent promoter DNA methylation inactivating genes in the HR pathway also occurs in non-EC NSGSTs, so acquisition of DNA methylation capacity could be a key step for NSGCT precursors to embark on the path toward EC and its differentiated lineages. However, because imprinting methylation is never properly re-established, early genetic, epigenetic, or genetic and epigenetic defects likely occur before the re-establishment of imprinting methylation (i.e., *in utero*).

Treatment refractory TGCT is rare and mortality is now low, but late effects of chemotherapy and morbidity associated with surgery remain a clinical challenge that several of our results may help to address. Despite considerable effort (Gilbert et al., 2016; Vergouwe et al., 2003), a need remains to identify, among patients with stage I NSGCTs, the 50%–70% of men without occult metastases who could be cured by orchiectomy alone. A panel interrogating circulating *miR-371* plus *miR-375* is envisioned for identifying patients free from residual disease following orchiectomy, who could be spared adjuvant chemotherapy from which they would receive no benefit. *miR-371*, already proposed for this purpose (Syring et al., 2015), was highly expressed in seminomas and EC, but expression was low in yolk sac tumors and minimal in teratomas. We found *miR-375* to be highly expressed in teratomas and yolk sac tumors, for which it is a promising serum marker because circulating levels are reportedly low in healthy young men (Zhang et al., 2015). *miR-375* alone also is a promising marker for identifying, among patients with residual masses ≥1 cm following chemotherapy for stage II tumors, the 55%–60% whose masses contain only scar tissue (Daneshmand et al., 2012). These patients presently undergo extraordinarily invasive surgery because they cannot be distinguished before the procedure from patients whose masses harbor teratoma cells, requiring surgery to achieve cure. Translational studies to validate these miRNAs as predictive serum markers could start to fill these significant unmet needs.

Other results suggest strategies for targeted therapy. DNA methyltransferase inhibitors in NSGCT could reprogram the epigenome into a hypomethylated state and induce immunogenicity. A study showed that cells from refractory TGCTs are highly sensitive to guadecitabine (Albany et al., 2017). *BRCA1* and *RAD51C* promoter methylation in 35% of non-EC NSGCTs makes a significant proportion of these tumors candidates for treatment with PARP inhibitors (Cavallo et al., 2012). Finally, recurrent epigenetic silencing of *DNAJC15/MCJ* in NGSCTs makes these genes candidate predictive markers, because their expression in breast and uterine cancer cells is reportedly associated with drug resistance (Fernández-Cabezudo et al., 2016).

We have provided a rich source of data from multiple platforms that describe a large set of well-characterized TGCTs. Integrative analysis identified numerous molecular features that distinguish each histology and reflect the histological composition of mixed tumors; it also identified molecularly defined subsets of seminomas associated with *KIT* mutations. These data afford a more complete view of previously articulated hypotheses, provide additional insights into mechanisms of TGCT tumorigenesis, and identify possible new approaches to the treatment of TGCTs.

## EXPERIMENTAL PROCEDURES

Tumor tissue and normal whole-blood samples were obtained from patients at contributing centers with informed consent, according to their local institutional review boards (IRBs). Biospecimens were centrally processed, and DNA, RNA, and protein were distributed to TCGA analysis centers.

TCGA project management has collected the necessary human subjects documentation to ensure that the project complies with 45 CFR 46 (the “Common Rule”). The program has obtained documentation from every contributing clinical site to verify that IRB approval has been obtained to participate in TCGA. Such documented approval may include one or more of the following items:

- An IRB-approved protocol with informed consent specific to TCGA or a substantially similar program. In the latter case, if the protocol was not TCGA specific, the clinical site’s principal investigator (PI) provided a further finding from the IRB that the already-approved protocol was sufficient to participate in TCGA.
- A TCGA-specific IRB waiver has been granted.
- A TCGA-specific letter that the IRB considers one of the exemptions in 45 CFR 46 to be applicable. The two most common exemptions cited were that the research falls under 46.102(f)(2) or 46.101(b)(4). Both exempt requirements for informed consent because the received data and material do not contain directly identifiable private information.
- A TCGA-specific letter that the IRB does not consider the use of these data and materials to be human subjects research. This was most common for collections in which the donors were deceased.

This study included 137 primary TGCTs and matched germline control DNA obtained from 133 male patients. The median age of diagnosis was 31 years, with a range of 14–67. Patient tumor histology was classified according to a consensus of expert pathologists. Molecular and genomic data were collected using reverse phase protein arrays (RPPAs), whole-exome DNA sequencing, RNA-seq, miRNA sequencing, DNA methylation arrays, and SNP arrays for copy number analysis. Detailed methods are provided in the Supplemental Experimental Procedures.

## Supplementary Material

1

2

3

4

5

6

7

8

## Figures and Tables

**Table 1:** Summary of Clinical, Epigenetic, and Molecular Alterations in TGCTs

		Seminoma		NSGCT-EC	NSGCT-Other		
		
		Seminoma *KIT* Mutant	Seminoma *KIT* Wild-Type	EC	Teratoma	Yolk Sac	Mixed NSGCTs
	Samples, no.	25	47	27	16	13	9

	Cryptorchidism, no. (%)	10 (40)	7 (14.9)^a^	2 (7.4)	1 (6.3)	1 (7.7)	1 (12.5)^a^

	Age at diagnosis, y, median (range)	34 (20–52)	31 (20–47)	26 (14–67)	28 (20–53)	31 (20–66)	28 (19–39)

DNA Methylation	Overall DNA methylation	fully unmethylated	residual methylation	ESC-like CpG + CpH	CpG methylation, CGI hypermethylation		

	Imprinting	fully erased	ully erased	fully erased; methylated *XLαs*	fully erased; methylated *NESP55*		

	Promoter Methylation						

	*BRCA1*, no. (%)	0	0	2 (7.4)	5 (31.2)	7 (53.8)	2 (22.2)

	*RAD51C*, no. (%)	0	0	3 (11.1)	5 (31.2)	4 (30.8)	4 (44.4)

	*MGMT*, no. (%)	0	0	3 (11.1)	9 (56.2)	5 (38.5)	4 (44.4)

	*DNAJC15*, no. (%)	0	0	0	8 (50)	4 (30.8)	1 (11.1)

	Leukocyte infiltration, median (range)	0.43 (0.04–0.69)	0.32 (0.004–0.58)	0.28 (0.08–0.47)	0.16 (0.01–0.46)	0.11 (0.01–0.36)	0.16 (0.11–0.37)

	Lymphocyte expression signatures	High	medium–high	low	low	low	low

	CTA expression	High	high	low	low	low	low

Mutations	Mutation rate, total mutations/Mb, median (range)	0.44 (0.12–1.28)	0.44 (0.26–1.07)	0.46 (0.09–1.78)	0.48 (0.20–0.73)	0.65 (0.26–1.41)	0.73 (0.29–1.83)

	C > T mutation at CpG sites	Low	low–medium	medium	medium	medium	medium

Copy Number	Purity (ABSOLUTE), median (range)	0.44 (0.18–0.8)^b^	0.46 (0.17–0.89)^b^	0.59 (0.16–0.92)	0.83 (0.36–0.92)	0.84 (0.47–0.93)	0.81 (0.4–0.91)

	Ploidy (ABSOLUTE), median (range)	3.01 (2.73–3.55)^b^	3.09 (2.67–4.97)^b^	2.84 (2.36–4.54)	2.67 (2.2–2.99)	2.78 (2.44–4.66)	2.8 (2.45–3.86)

	i(12p), no. (%)	12 (60)^b^	37 (80)^b^	27 (100)	16 (100)	13 (100)	9 (100)

	12p copies, median (range)	5 (4–8)^b^	7 (4–14)^b^	8 (5–12)	7 (4–9)	7 (5–17)	7 (5–7)

miRNA	miR 19q13.42 cluster	Low	low	high	low	low	low

	miR-371a-3p	High	high	high	low	moderate	high

	miR-375	Low	low	moderate	high	high	high

KIT/KRAS Pathway	*KIT* mutations, no. (%)	25 (100)	0	0	0	0	0

	*KIT* copies, median (range)	3 (2–8)^b^	2 (2–12)^b^	2 (2–16)	2 (2–4)	2 (2–6)	2 (2–3)

	*KIT* mRNA	High	high	low	low	low	low

	KIT protein	High	high	low	low	low	low

	*KITLG* mRNA	Low	low	moderate	high	high	high

	*KRAS* mutations, no. (%)	4 (15.4)	14 (29.8)	0	1 (6.3)^c^	0	0

	*KRAS* copies, median (range)	5 (4–8)^b^	7 (4–R60)^b^	7 (5–13)	6.5 (4–9)	8 (5–17)	7 (5–53)^d^

	*KRAS* mRNA	Low	high	high	low	low	low

	*CBL* copies, average (range)	2.1 (1–3)^b^	1.6 (1–3)^b^	2.1 (1–3)	1.9 (1–2)	2.4 (2–4)	2.1 (1–3)

	miR-222-3p	low	low	high	low	moderate	high

See also Tables S1 and S2.

a Missing data for cryptorchidism (seminoma WT n = 6, Mixed Nseminoma n = 1).

b Six samples with low purity and inability to estimate copy number (5 KIT mutations, 1 KIT WT). Samples excluded from analysis.

c KRAS mutation in mature teratoma-dominant sample with 30% seminoma component.

d Sample with 53 copies of KRAS had 55% seminoma component.

## Highlights

- *KIT*-mutated seminoma has distinct DNA methylation and immune infiltration profiles
- DNA methylation and miRNA expression differ greatly between histology types
- Significant somatic mutations are present only in TGCTs with seminoma components
- All histology types exhibit extensive aneuploidy and low mutation frequency
