# Supplementary material for: Integrated Molecular Characterization of Testicular Germ Cell Tumors
Source: Cell Rep. Author manuscript; Available in PMC 2018 Aug 3. (PMC6075738; doi:10.1016/j.celrep.2018.05.039)
Supplement: 1 [file NIHMS977848-supplement-1.pdf]

Cell Reports, Volume *23*

Supplemental Information

Integrated Molecular Characterization of Testicular Germ Cell Tumors

Hui Shen, Juliann Shih, Daniel P. Hollern, Linghua Wang, Reanne Bowlby, Satish K. Tickoo, Vésteinn Thorsson, Andrew J. Mungall, Yulia Newton, Apurva M. Hegde, Joshua Armenia, Francisco Sánchez-Vega, John Pluta, Louise C. Pyle, Rohit Mehra, Victor E. Reuter, Guilherme Godoy, Jeffrey Jones, Carl S. Shelley, Darren R. Feldman, Daniel O. Vidal, Davor Lessel, Tomislav Kulis, Flavio M. Cárcano, Kristen M. Leraas, Tara M. Lichtenberg, Denise Brooks, Andrew D. Cherniack, Juok Cho, David I. Heiman, Katayoon Kasaian, Minwei Liu, Michael S. Noble, Liu Xi, Hailei Zhang, Wanding Zhou, Jean C. ZenKlusen, Carolyn M. Hutter, Ina Felau, Jiashan Zhang, Nikolaus Schultz, Gad Getz, Matthew Meyerson, Joshua M. Stuart, The Cancer Genome Atlas Research Network, Rehan Akbani, David A. Wheeler, Peter W. Laird, Katherine L. Nathanson, Victoria K. Cortessis, and Katherine A. Hoadley

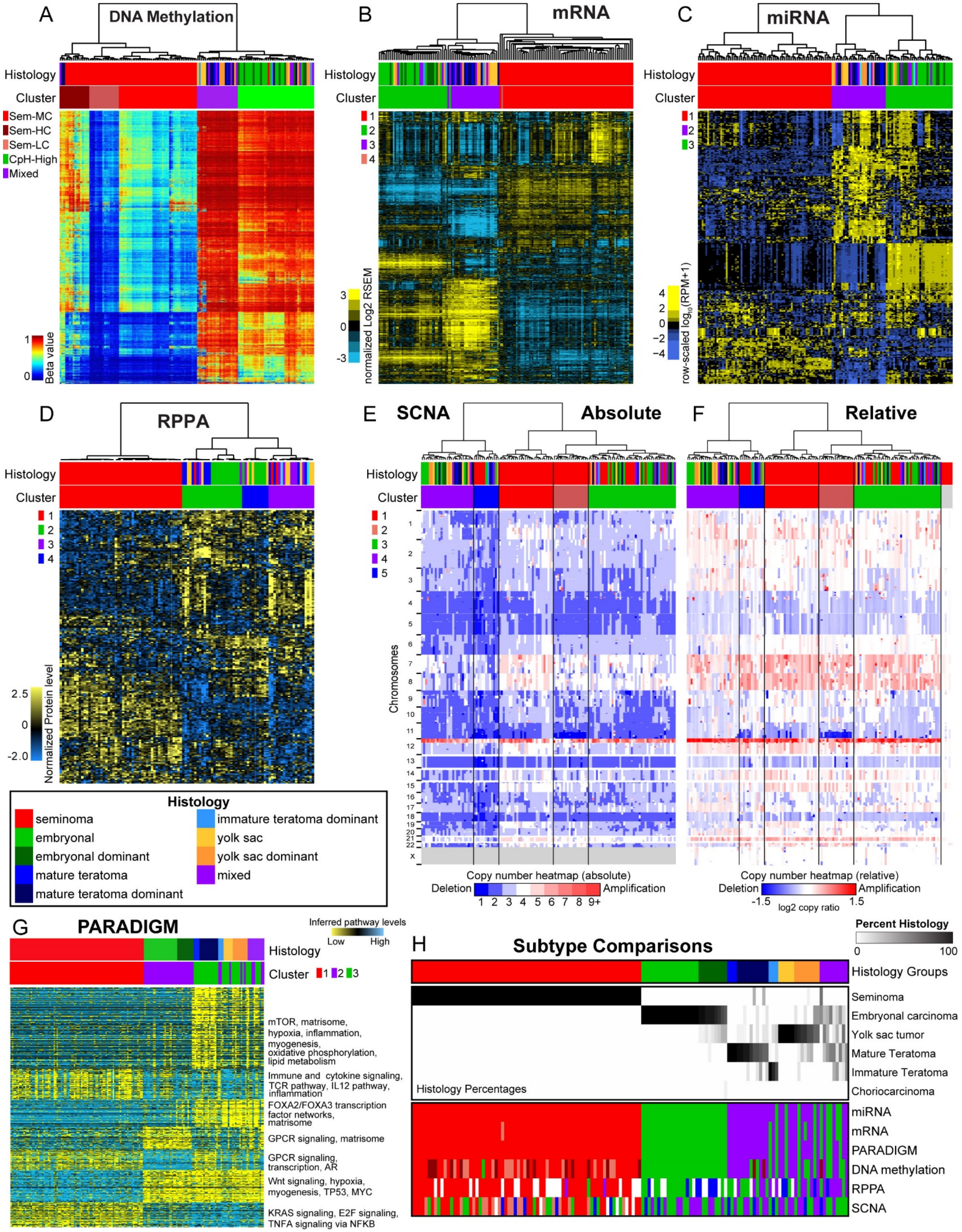
A
DNA Methylation
Histology
Cluster
Sem-MC
Sem-HC
Sem-LC
CpH-High
Mixed
Beta value
B
mRNA
normalized Log2 RSEM
C
miRNA
row-scaled log10(RPM+1)
D
RPPA
Normalized Protein level
E
SCNA
Absolute
Chromosomes
Copy number heatmap (absolute)
Deletion
Amplification
F
Relative
Copy number heatmap (relative)
log2 copy ratio
Histology
seminoma
embryonal
embryonal dominant
mature teratoma
mature teratoma dominant
immature teratoma dominant
yolk sac
yolk sac dominant
mixed
G
PARADIGM
Inferred pathway levels
Low
High
mTOR, matrisome, hypoxia, inflammation, myogenesis, oxidative phosphorylation, lipid metabolism
Immune and cytokine signaling, TCR pathway, IL12 pathway, inflammation
FOXA2/FOXA3 transcription factor networks, matrisome
GPCR signaling, matrisome
GPCR signaling, transcription, AR
Wnt signaling, hypoxia, myogenesis, TP53, MYC
KRAS signaling, E2F signaling, TNFA signaling via NFKB
H
Subtype Comparisons
Percent Histology
Histology Groups
Seminoma
Embryonal carcinoma
Yolk sac tumor
Mature Teratoma
Immature Teratoma
Choriocarcinoma
Histology Percentages
miRNA
mRNA
PARADIGM
DNA methylation
RPPA
SCNA

Figure S1. Related to Figure 1 and Table S1. Molecular classification of TGCT. A) DNA methylation, unsupervised clustering of 9,614 variably methylated autosomal CpG probes identifies five clusters predominantly separating histological types. **B) mRNA,** unsupervised consensus hierarchical clustering of 2,787 variably and highly expressed genes identified three main clusters with high concordance to histology types. **C) miRNA,** unsupervised hierarchical clustering of the top 25% most variable miRNA 5p or 3p strands identified 3 clusters. **D) Protein,** unsupervised clustering of RPPA data for 104 samples and 218 antibodies identified four clusters. **E,F) Copy number,** unsupervised hierarchical clustering of arm-level somatic copy number alterations separates 137 TGCT tumors into five groups. The heatmaps show absolute (total integer copies, **E**) and relative copy number (corrected to average sample ploidy, **F**), with chromosomes ordered from top to bottom. **G) Paradigm,** clustering of protein coding inferred pathway levels (IPLs) produced by PARADIGM integrating mRNA and copy number identified 3 subtypes. Distinct groups of genes are enriched in various pathways, annotated to the right of the gene group. **H)** Comparison of histology groups, individual histology percentages, and molecular subtypes.

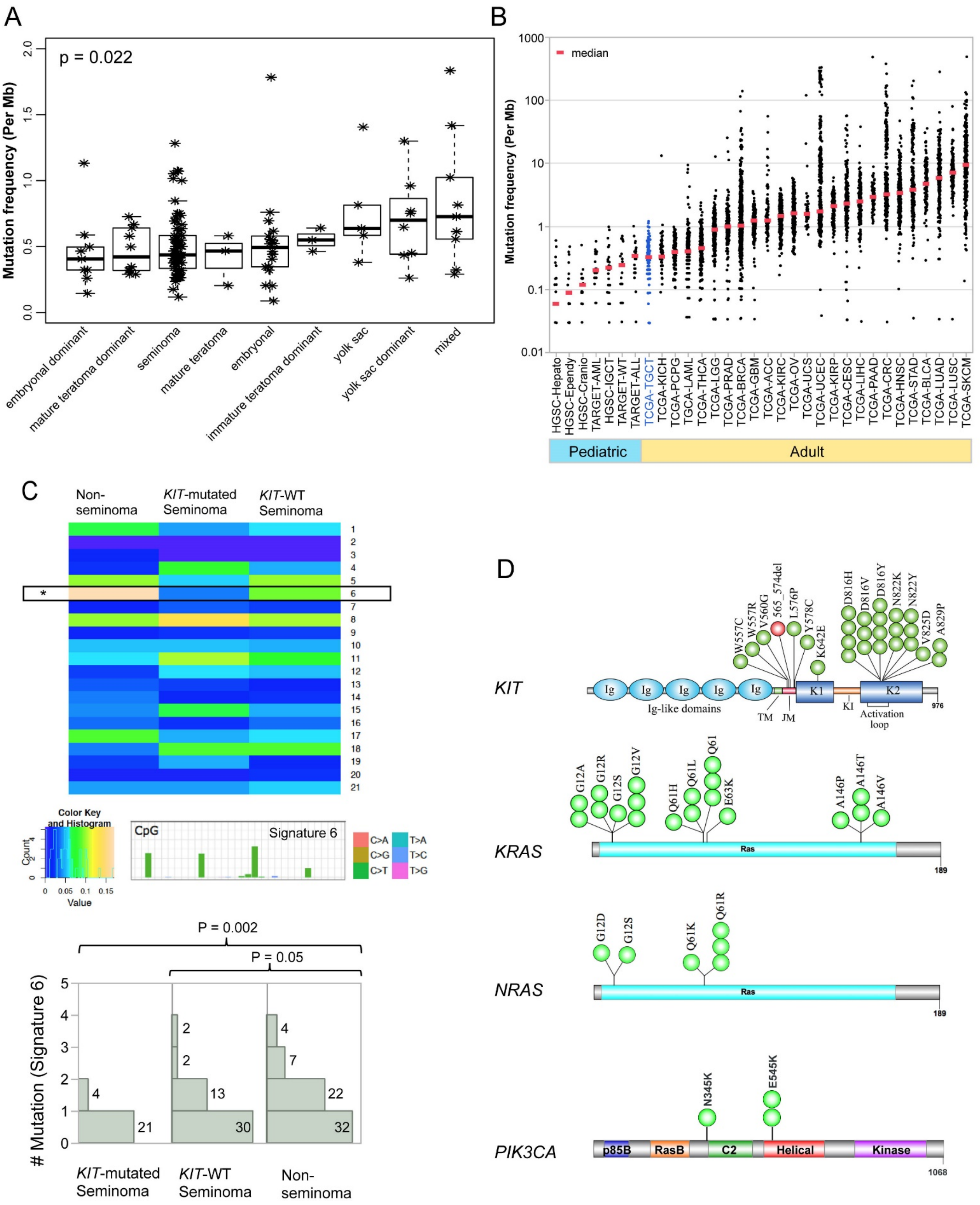

Figure S2. Related to Figure 2 and Table S1. Somatic mutations and mutational signatures in TGCT. A) Total somatic mutation rate across histological subtypes of TGCT, sorted from lowest to highest median mutation frequency by histology. **B)** Total somatic mutation rate across 7 pediatric and 25 adult tumor types, sorted from lowest to median mutation frequency. TGCT is highlighted in blue. **C)** Increased mutational signature 6 observed in non-seminomas. The heatmap shows the mutational signatures across three TGCT groups, defined by histology and *KIT* mutation status. Somatic mutations were pooled from tumors within the same group for signature analysis. Of 21 cancer mutation signatures (Covington and Wheeler, 2015), Signature 6 (C>T substitutions at CpG

dinucleotides suggestive of the number of cell replications) was the only one for which mutation frequencies were significantly different across groups. The middle panel shows the relative proportions of each base change that characterize Signature 6 displayed according to the 96 sequence contexts immediately 3’ and 5’ to the mutated base. Lower panel, somatic mutations attributable to Signature 6. The bar chart displays the number of somatic mutations in each sample with a Signature 6 mutation profile. P-values are calculated for each pair of groups using the Wilcoxon *T* test. D**)** Schematic representation of somatic mutations identified in *KIT*, *KRAS*, *NRAS*, and *PIK3CA*. Missense mutation, green circle; INDEL, red circle.

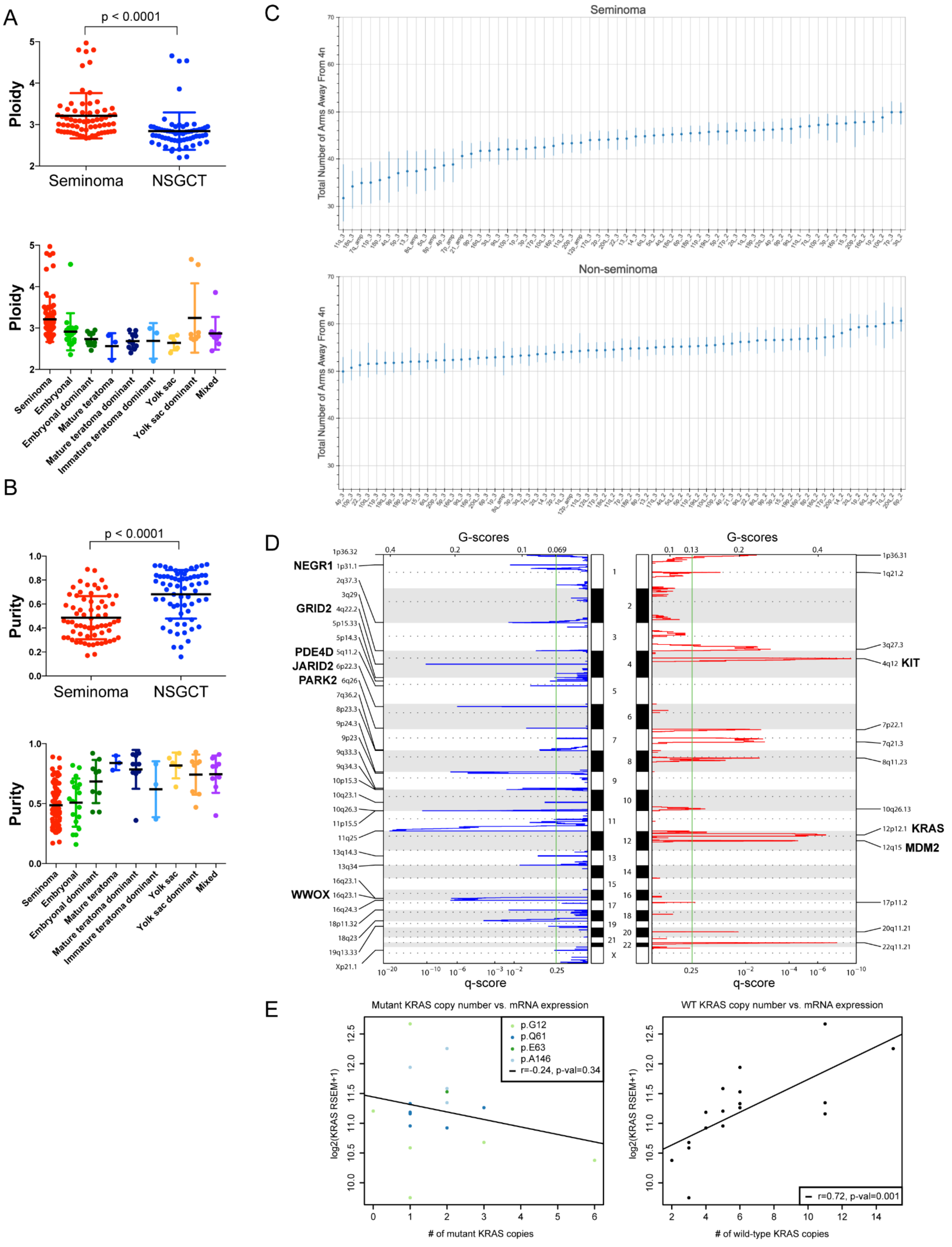

A
p < 0.0001
Ploidy
Seminoma
NSGCT
Ploidy
Seminoma
Embryonal
Embryonal dominant
Mature teratoma
Mature teratoma dominant
Immature teratoma dominant
Yolk sac
Yolk sac dominant
Mixed
B
p < 0.0001
Purity
Seminoma
NSGCT
Purity
Seminoma
Embryonal
Embryonal dominant
Mature teratoma
Mature teratoma dominant
Immature teratoma dominant
Yolk sac
Yolk sac dominant
Mixed
C
Seminoma
Total Number of Arms Away From 4n
Non-seminoma
Total Number of Arms Away From 4n
D
G-scores
G-scores
NEGR1
GRID2
PDE4D
JARID2
PARK2
WWOX
KIT
KRAS
MDM2
q-score
q-score
E
Mutant KRAS copy number vs. mRNA expression
p.G12
p.Q61
p.E63
p.A146
r=-0.24, p-val=0.34
log2(KRAS RSEM+1)
# of mutant KRAS copies
WT KRAS copy number vs. mRNA expression
r=0.72, p-val=0.001
log2(KRAS RSEM+1)
# of wild-type KRAS copies

Figure S3. Related to Figure 2 and Tables S1 and S3. Copy number alterations and characteristics differ across TGCT histology types. A) Tumor purity and **B)** ploidy across SCNA clusters by histology. **C)** Significant focal copy number alterations by GISTIC 2.0 analysis. Left: significantly amplified genomic regions; known oncogenes *KIT, KRAS*, and *MDM2* are within significant peaks as noted. Right: significantly deleted genomic regions; known fragile sites *NEGR1, GRID2, PDE4D, JARID2, PARK2,* and *WWOX* are within significant peaks as noted. **D)** Timing of arm-level events in groups of TGCT. Arm-level copy number alterations with at least seven occurrences across samples ordered from left to right by the mean "aneuploidy score" (total number of arms away from tetraploidy) of tumors possessing the alteration. Because tumors tend to gain aneuploidy or chromosomal instability over the course of tumorigenesis, "aneuploidy score" acts as a surrogate for relative timing, with events sorted from earliest to latest from left to right. The span of each vertical line represents bootstrapped 95% confidence intervals. **E)** DNA copies of wild-type *KRAS*, but not copies of mutant *KRAS*, are associated with *KRAS* gene expression. *KRAS* mRNA expression by the number of mutant (left) or wild-type (WT, right) *KRAS* copies present in each of the 17/19 tumors with copy number calls. Each tumor is colored by the identity of the mutant *KRAS* allele it possesses. Pearson's correlation and p-value are reported (right).

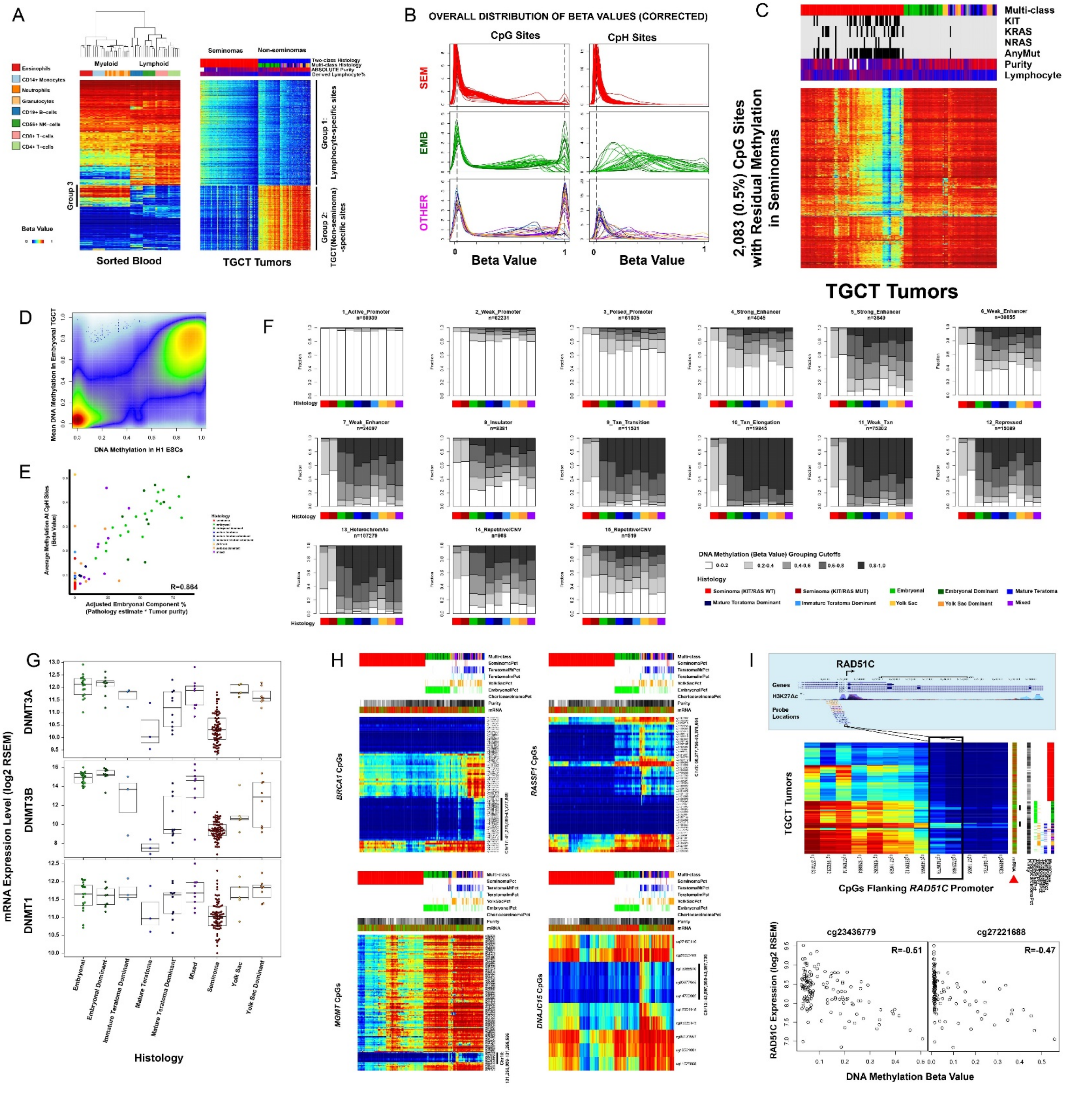

Figure S4. Related to Figure 4 and Table S1. ***DNA methylation patterns across TGCT*. A)** Lymphocyte-specific DNA methylation fingerprint is present in the TGCT tumors, especially seminomas. DNA methylation levels for 1,211 probes were plotted in the same order for sorted blood populations (left panel, samples ordered by hierarchical clustering) and TGCT (right panel, samples ordered by histology, then lymphocytic methylation signature). Lymphocytic-specific probes (Group 1, n=719 probes) were chosen by selecting CpG sites with a high DNA methylation level in lymphocytes (mean beta value >0.7) and low in non-seminomas (mean beta value <0.3). As a control, TGCT (Non-seminoma)-specific probes (Group 2, n=472) were chosen by selecting CpG sites with low DNA methylation level in lymphocytes (<0.3) and high DNA methylation level in non-seminomas (>0.7). Within Group 2, the myeloid DNA-methylation signature (Group 3) was not observed in seminoma. The kernel-smoothed DNA methylation distribution at these sites is used as a surrogate for lymphocyte fraction and is plotted as the bottom column-side color bar on the right panel (labeled

‘Derived Lymphocyte %’; blue to red – low to high lymphocyte %). **B)** Overall CpH and CpG methylation distributions after correction for lymphocyte methylation reveal almost complete lack of methylation in seminomas but not in other subtypes. Methylated peaks in seminomas disappear after lymphocyte methylation subtraction, while this correction does not eliminate the methylated peaks in non-seminomas. The color of each smoothed density plot lines indicates the annotated histology of the corresponding sample. **C)** Residual DNA methylation in seminomas differs based on *KIT*/*RAS* mutation status before correction for lymphocyte methylation. A heatmap shows 2,083 (0.5%) loci (rows) with residual methylation in seminomas (columns). Blue to red indicates 0% to 100% methylation, before correction for lymphocyte methylation. Top color bars annotate the histology of each tumor and mutation status in *KIT/KRAS/NRAS* (black – mutants; gray – wildtype). **D)** Comparison of global DNA methylation in embryonal and embryonal dominant TGCT tumors and H1 ESC. DNA methylation levels in H1 ESCs from ENCODE (x-axis) for all HM450 probes are plotted against the average DNA methylation levels in embryonal and embryonal dominant TGCT tumors (y-axis). Color indicates smoothed density of CpGs/probes, where blue indicates lowest and red indicates highest density. **E)** Average CpH methylation highly correlates with percent embryonal carcinoma component. The x-axis represents adjusted embryonal carcinoma component percentage, calculated as pathologists’ estimate of embryonal carcinoma component percentage within the tumor compartment, multiplied by ABSOLUTE-based tumor purity estimate (to derive the percentage of embryonal carcinoma component in the bulk DNA). The y-axis denotes the average CpH methylation level for the corresponding tumor, while the color of the tumor indicates its histological classification. Pearson’s correlation coefficient (R) between the two measurements is denoted in the lower right. **F)** Distribution of DNA methylation in the genome. The genome is segmented into different chromatin states based on the ChromHMM algorithm {Ernst, 2012 #81} applied to ChIP-seq results obtained from H1 embryonic stem cells (ESCs). Probes/CpGs that overlap with each chromatin state are plotted as one panel and the total number of probes for each panel is indicated below the name of the corresponding chromatin state. TGCT tumors with sufficient purity (>0.7 based on ASBOLUTE estimates) are grouped into ten histology groups and within each group, the fractions of CpGs with median DNA methylation levels within five consecutive ranges (0-0.2, 0.2-0.4,0.4-0.6,0.6-0.8,0.8-1.0) are shown with bar charts. Seminomas are split into KIT/RAS wildtype (WT) cases and mutant cases (MUT). **G)** The *de novo* DNA methyltransferases DNMT3A/B are overexpressed in embyronal and embryonal dominant TGCT tumors. mRNA expression levels (log2 RSEM) for the *de novo* DNA methyltransferases DNMT3A, DNMT3B and maintenance DNA methyltransferase DNMT1 are plotted as boxplots, with dot plots showing each tumor. **H)** Promoter hypermethylation and/or epigenetic silencing of *BRCA1*, *RASSF1*, *DNAJC15*, and *MGMT* (clockwise from top right). DNA methylation levels (blue – red: low to high level of DNA methylation) of all probes located within (-1500, +200) bp of all transcripts mapped to each gene (rows; sorted by increasing genomic location from bottom to top) for each sample (columns) are shown as a heatmap. Selected features of each sample are plotted as column-side color bars with the same notations used in other figures. In particular, an mRNA expression bar (green – red: low – high level of expression) is plotted, to show that samples with hypermethylation across the region of interest have low expression. A vertical bar indicates the location of the region showing cancer-specific hypermethylation together with the corresponding hg19/GRCh37 coordinates. **I)** Epigenetic silencing of *RAD51C*. Upper panel – a genomic view of the *RAD51C* gene. H3K27Ac peaks indicate two separate regulatory elements. Locations of probes plotted in Panel B are indicated in relation to the presumed *RAD51C* promoter. Middle panel – heatmap showing the DNA methylation level (blue – red: low to high level of DNA methylation) of all probes located within +/- 1500bp of the *RAD51C* TSS. Hypermethylation at two CpGs (black box) from the first H3K27Ac peak closest to the TSS is observed. Samples with methylation at these sites also have low mRNA expression (mRNA bar indicated by a red triangle; green to red color represent low to high level of mRNA expression). Bottom panel – scatterplots of the *RAD51C* mRNA level (y-axis) versus DNA methylation level (x-axis) at these two CpG sites demonstrate decreased expression with increased methylation.

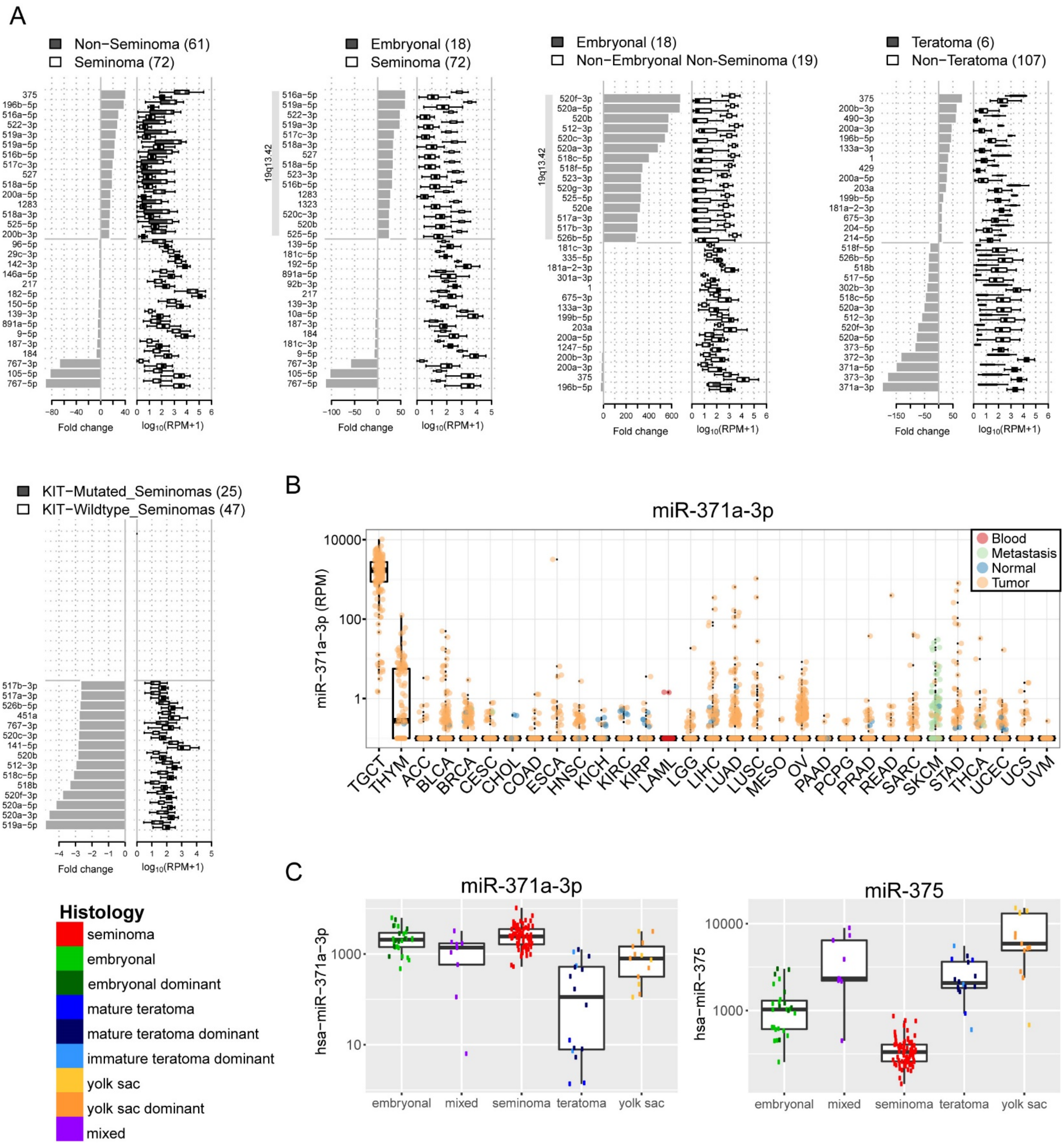

Figure S5. Related to Figure 1 and Tables S1, S4, and S5. miRNA analysis of 137 TGCTs. A) Differentially abundant miRNAs between histological types. Each panel has (left) a barplot of median-based fold change, and (right) boxplots showing distributions of normalized (RPM) abundance, with black vertical lines indicating medians. Up to 15 of the largest fold changes in each direction are shown. The numbers of samples in each group are in parentheses. Because miRNAs with higher abundance are likely more influential (Mullokandov 2012, Tay 2014), the graph shows only miRNAs that have a mean abundance of at least 50 RPM. **B)** TGCT-specific expression of miR-371a-3p in TCGA. RPM abundance of miR-371a-3p across TCGA tumor and normal samples is shown, sorted from left to right by decreasing median. **C)** Two additional discriminatory miRNAs (miR-371a-3p and miR-375) based on differential expression analysis are shown.

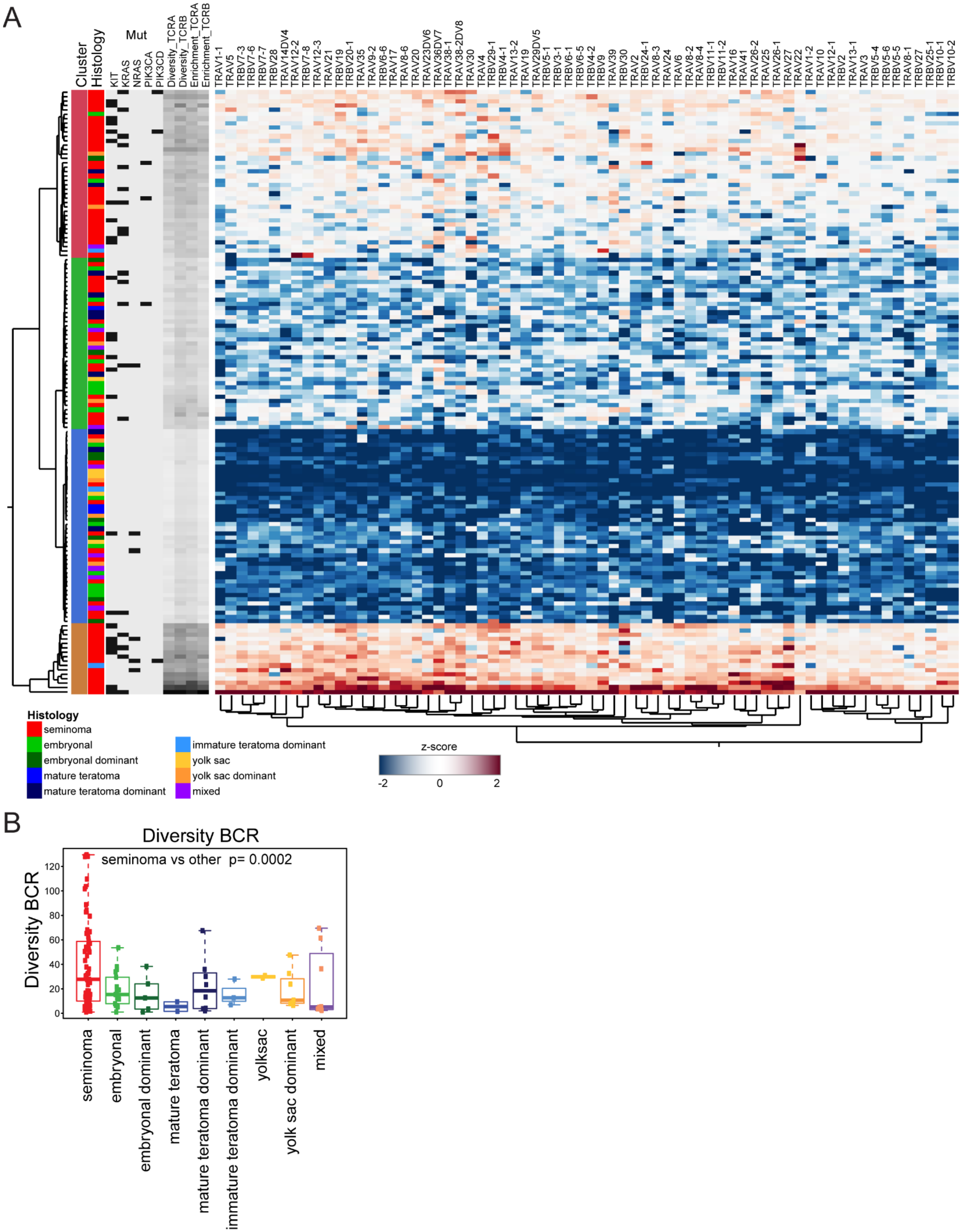

A
Cluster
Histology
Mut
KIT
KRAS
NRAS
PIK3CA
PIK3CD
Diversity_TCRA
Diversity_TCRB
Enrichment_TCRA
Enrichment_TCRB
TRAV1-1
TRAV5
TRBV7-3
TRBV7-6
TRBV7-7
TRBV28
TRAV14DV4
TRAV12-2
TRBV7-8
TRAV12-3
TRAV21
TRBV19
TRBV20-1
TRAV35
TRAV9-2
TRBV6-6
TRAV17
TRAV8-6
TRAV20
TRAV23DV6
TRAV36DV7
TRAV38-1
TRAV38-2DV8
TRAV30
TRAV4
TRBV29-1
TRBV4-1
TRAV13-2
TRAV19
TRAV29DV5
TRBV5-1
TRBV3-1
TRBV6-1
TRBV6-5
TRBV4-2
TRBV9
TRAV39
TRBV30
TRAV2
TRBV24-1
TRAV8-3
TRAV24
TRAV6
TRAV8-2
TRAV8-4
TRBV11-1
TRBV11-2
TRAV16
TRAV41
TRAV26-2
TRAV25
TRAV26-1
TRAV27
TRAV22
TRAV1-2
TRAV10
TRAV12-1
TRBV2
TRAV13-1
TRAV3
TRBV5-4
TRBV5-6
TRBV5-5
TRAV8-1
TRBV27
TRBV25-1
TRBV10-1
TRBV10-2
Histology
seminoma
embryonal
embryonal dominant
mature teratoma
mature teratoma dominant
immature teratoma dominant
yolk sac
yolk sac dominant
mixed
z-score
-2
0
2
B
Diversity BCR
seminoma vs other p= 0.0002
Diversity BCR
0
20
40
60
80
100
120
seminoma
embryonal
embryonal dominant
mature teratoma
mature teratoma dominant
immature teratoma dominant
yolksac
yolk sac dominant
mixed

Figure S6. Related to Figure 5. T-Cell and B-Cell sequence diversity across TGCTs and key molecular associations with immune infiltration in seminomas. A) The diversity, enrichment, and expression of T-cell receptor across testicular germ cell. **B)** B-cell receptor diversity across histological types testicular germs tumors.

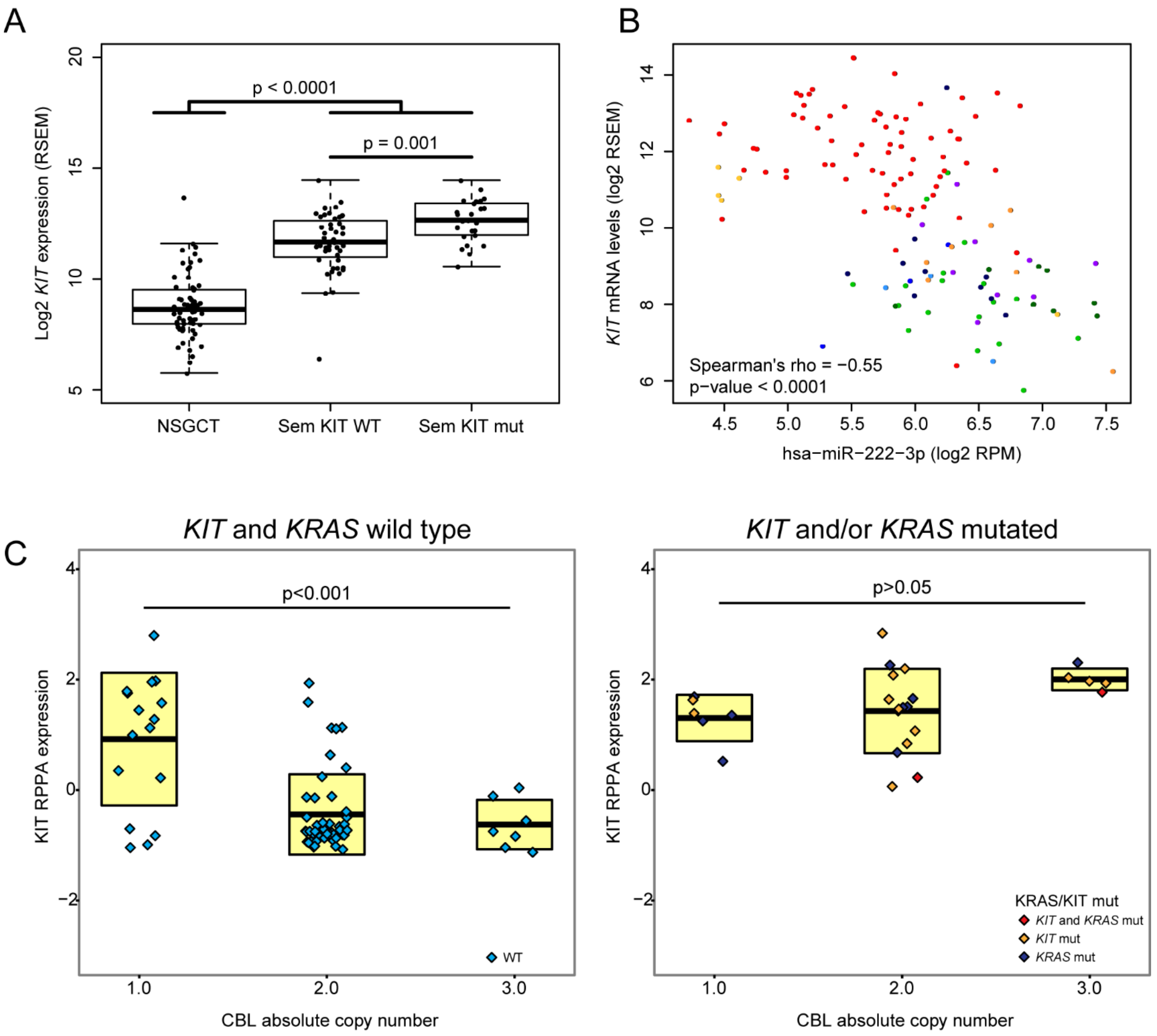

Figure S7. Related to Figure 6 and Table S1. Somatic alterations in *KIT* pathway genes. A) *KIT* gene expression is higher in in Sem and SEM with *KIT*-mutated. Nonparametric comparisons for each pair were done using the Wilcoxon Method to calculate the p values. **B)** Negative correlation between has-miR-222-3p and *KIT* mRNA expression. Spearman's rho and p-values were calculated. **C)** *CBL* copy number is negatively correlated with *KIT* RPPA expression in *KIT/KRAS* wild-type tumors (left), but not in *KIT/KRAS* mutated tumors (right). Tumors with mutations in *KIT*, *KRAS*, or both are indicated in yellow, dark blue, and red respectively and those wild-type (WT) for both genes are in light blue.

Table S1. Related to Table 1. Detailed Patient Characteristics.

Table S2. Related to Table 1. Patient Summary Characteristics.

	Histology of First or Only TGCT	
	Sem[a] (N=68)	NSGCT[b] (N=65)
Age at first or only diagnosis (median in years)	33	28
Race		
European descent (89%)	59	59
African descent (5%)	3	3
Asian descent (3%)	3	1
Unknown (4%)	3	2
Ethnicity		
Hispanic or Latino (9%)	6	6
Non-Hispanic (83%)	58	52
Unknown (8%)	4	7
Family history of any cancer (41%)	35	19
Family history of TGCT (11%)	7	7
Personal history of cryptorchidism (17%)	17	5
Detailed TGCT histology		
Seminoma[c]	68	-
Embryonal carcinoma[c]	-	18
Embryonal carcinoma dominant[d]	-	9
Immature teratoma dominant[d]	-	3
Mature teratoma[c]	-	3
Mature teratoma dominant[c]	-	10
Yolk sac[c]	-	5
Yolk sac dominant[d]	-	8
Mixed[e]	-	9
History of two primary TGCTs (7%)	3	6
Tumor Clinical Stage at diagnosis		
Stage I (70%)	52	41
Stage II-III (23%)	9	22
Unknown (7%)	7	2
Clinical outcomes		
Local recurrence (15%)	7	13
Distant metastasis (5%)	1	5

[a] 100% seminoma,
[b] Any histology except 100% seminoma
[c] 100% named histology
[d] >60% named histology
[e] No single dominant histology

Table S3. Related to Figure 2 and 3. Inferred integer copy number values by arm per tumor.

Table S4. Related to Figures 1, S1, and S5. Differentially abundant miRNAs by histology type.

Table S5. Related to Figures 1 and S5. Random forest classification out for miRNAs available to distinguish seminoma, embryonal carcinoma, and NSGCT.

Table S6. Related to Figures 1 and S1. Differentially expressed mRNAs by histology types.

Table S7. Related to Figures 1 and S1. Differentially expressed proteins by histology types.

Supplemental Experimental Methods

Sample Acquisition

Patients with testicular germ cell tumors were enrolled into the TCGA from 15 referral centers (Analytical Biological Services, Inc. (Indianapolis, IN, USA); Barretos Cancer Hospital (Barretos, Brazil); two contributing sites from Baylor (Houston, TX, USA); Cleveland Clinic (Cleveland, OH, USA); Erasmus Medical Center (Rotterdam, Netherlands); Gundersen Lutheran (La Crosse, WI, USA); International Genomics Consortium (Phoenix, AZ, USA); ProteoGenex (Culver City, CA, USA); Spectrum Health (Grand Rapids, MI, USA); University of Minnesota (Minneapolis, MN, USA); University of North Carolina (Chapel Hill, NC, USA); University of Pennsylvania (Philadelphia, PA, USA); University of Southern California (Los Angeles, CA, USA); University of Ulm (Ulm, Germany)) under IRB-approved protocols. Spermatocytic seminoma cases were excluded from this study. Primary tumor samples and matched germline control DNA (blood or blood components, including DNA extracted at the submitting site) were obtained from patients who had received no prior treatment for their disease (chemotherapy or radiotherapy; exception allowed for second primary tumor collection). Specimens were shipped overnight to the Biospecimen Core Resource (BCR) using a cryoport that maintained an average temperature of less than -180°C.

Cases were staged according to the American Joint Committee on Cancer (AJCC) staging system and, if the patient had received chemotherapy, the International Germ Cell Cancer Collaborative Group (IGCCG) staging. Pathology quality control was performed on each tumor specimen from either a frozen section slide prepared by the BCR or from a permanent section taken from a FFPE block immediately adjacent to the submitted frozen tumor specimen. Hematoxylin and eosin (H&E) stained sections from each sample were subjected to independent pathology review to confirm that the tumor specimen was histologically consistent the reported testicular germ cell histology. The percent rumor nuclei, percent necrosis, and other pathology annotations were also assessed. Tumor samples with ≥60% tumor nuclei and ≤20% necrosis were submitted for nucleic acid extraction.

TCGA Project Management has collected necessary human subjects documentation to ensure the project complies with 45-CFR-46 (the "Common Rule"). The program has obtained documentation from every contributing clinical site to verify that IRB approval has been obtained to participate in TCGA. Such documented approval may include one or more of the following:

- An IRB-approved protocol with Informed Consent specific to TCGA or a substantially similar program. In the latter case, if the protocol was not TCGA-specific, the clinical site PI provided a further finding from the IRB that the already-approved protocol is sufficient to participate in TCGA.
- A TCGA-specific IRB waiver has been granted.
- A TCGA-specific letter that the IRB considers one of the exemptions in 45-CFR-46 applicable. The two most common exemptions cited were are that the research fall under 46.102(f)(2) or 46.101(b)(4). Both exempt requirements for informed consent because the received data and material do not contain directly identifiable private information.
- A TCGA-specific letter that the IRB does not consider the use of these data and materials to be human subjects research. This was most common for collections in which the donors were deceased.

Sample Processing

DNA and RNA were extracted and quality was assessed at the central BCR. RNA and DNA were extracted from tumor using a modification of the DNA/RNA AllPrep kit (Qiagen). The flow-through from the Qiagen DNA column was processed using a *mir*Vana miRNA Isolation Kit (Ambion). This latter step generated RNA preparations that included RNA <200 nt suitable for miRNA analysis. DNA was extracted from blood using the QiaAmp DNA Blood Midi kit (Qiagen).

RNA samples were quantified by measuring Abs_{260} with a UV spectrophotometer and DNA quantified by PicoGreen assay. DNA specimens were resolved by 1% agarose gel electrophoresis to confirm high molecular weight fragments. A custom Sequenom SNP panel or the AmpFISTR Identifiler (Applied Biosystems) was utilized to verify that tumor DNA and germline DNA representing a case were derived from the same patient. Five hundred nanograms of each tumor and germline DNA were sent to Qiagen (Hilden, Germany) for REPLI-g whole genome amplification using a 100 µg reaction scale. RNA was analyzed via the RNA6000 Nano assay (Agilent) for determination of an RNA Integrity Number (RIN), and only analytes with a RIN≥7.0 were included in this study. Only cases yielding a minimum of 6.9 µg of tumor DNA, 5.15 µg RNA, and 4.9 µg of germline DNA were included in this study.

Sample Qualification

The BCR received tumor samples with germline controls from a total of 379 cases, of which 296 cases qualified. Of these qualified cases, 150 were sent for genomic analysis. Of the 83 that disqualified, 1 case was an unacceptable diagnosis, 4 cases had insufficient tumor nuclei (<60%), 2 cases had excessive necrosis (≥20%), 3 had both insufficient tumor nuclei and excessive necrosis. Another 3 had a genotypic mismatch between the tumor and germline samples, 39 had RNA integrity scores of <7.0, and the remaining had insufficient nucleic acid yields.

Twelve cases had sufficient residual tumor tissue following extraction of nucleic acids to allow for proteomics analysis. A 10 to 20 mg piece of snap-frozen tumor adjacent to the piece used for molecular sequencing and characterization was submitted to MD Anderson for reverse phase protein array (RPPA analysis).

Clinical Data

The clinical data collected included patient age, sex, race, ethnicity, height, weight, histologic diagnoses and percentage of each in the submitted tumor, tumor anatomic location and extent, history of undescended tesis and related correction information, history of hypospadias, pre- and post-treatment serum marker levels, clinical and pathologic AJCC staging, IGCCCG staging, history of prior cancers, synchronous cancers and subsequent cancers including distant metastasis or second primary cancers, date of death, and date of last contact.

Expert Pathology Committee Histologic Evaluation of Testicular Germ Cell Tumors

Hematoxylin and eosin (H&E) stained sections from each frozen tissue sample were subjected to independent pathology review by an expert pathology panel consisting of 4 genitourinary pathologists. Each pathologist independently reported the germ cell tumor component/s and the percentage of each component present in the frozen tissue block. The diagnosis was considered "consensus" when at least 3 pathologists agreed on the tumor components and their percentage (within a range of 10%) in the tissue block. In the small number of cases with non-consensus diagnosis (primarily because of less than ideal quality of the frozen tissue sections), the slides from the frozen tissue block were re-evaluated along with formalin-fixed, paraffin-embedded permanent tissue sections from the case to arrive at a final diagnosis.

Tumors were classified into histology groups. Tumors were classified as "pure" if they contained 100% of the histology on the EPC review and dominant if the sample had at least 60% of a given histology type. Samples with no dominant histology were classified as mixed.

Exome Mutation Analysis and Validation

<u>WES library preparation</u>

DNA samples were constructed into Illumina paired-end (PE) pre-capture libraries according to the manufacture's protocol (Illumina Multiplexing_SamplePrep_Guide_1005361_D) with modifications as described in the BCM-HGSC Illumina Barcoded Paired-End Capture Library Preparation protocol. Libraries were prepared using Beckman robotic workstations (Biomek NXp and FXp models). The complete protocol and oligonucleotide sequences are accessible from the HGSC website https://www.hgsc.bcm.edu/sites/default/files/documents/Illumina_Barcoded_Paired-End_Capture_Library_Preparation.pdf. Briefly, 1 μg DNA was sheared into fragments of 200–300 base pairs in a Covaris plate with E210 system (Covaris, Inc. Woburn, MA) followed by end-repair, A-tailing and ligation of Illumina multiplexing PE adaptors. Pre-capture Ligation Mediated-PCR (LM-PCR) was performed using the Library Amplification Readymix containing KAPA HiFi DNA Polymerase (Kapa Biosystems, Cat. No. KK2612). Universal primer IMUX-P1.0 and a pre-capture barcoded primer IBC were used in the PCR amplification. In total, a set of 12 such barcoded primers were used on these samples. Purification was performed with Agencourt AMPure XP beads after enzymatic reactions. Following the final XP beads purification, quantification and size distribution of the pre-capture LM-PCR product was determined using the LabChip GX electrophoresis system (PerkinElmer) and gel analysis using AlphaView SA v3.4 software.

<u>Exome capture</u>

For the hybridization step, four pre-capture libraries were pooled together (~250 ng/sample). These pooled libraries were then hybridized in solution to the HGSC VCRome 2.1 design (42Mb, NimbleGen) according to the manufacturer's protocol NimbleGen SeqCap EZ Exome Library SR User's Guide (Version 2.2) with minor revisions(Bainbridge et al., 2011). Human COT1 DNA and full-length Illumina adaptor-specific blocking oligonucleotides were added into the hybridization to block repetitive genomic sequences and the adaptor sequences. For post-capture LM-PCR amplification, either matching KAPA HiFi DNA Polymerase or Phusion PCR Supermix HiFi (2×) was used. After the final AMPure XP bead purification, quantity and size of the capture library was analyzed using the Agilent Bioanalyzer 2100 DNA Chip 7500. The efficiency of the capture was evaluated by performing a qPCR-based quality check on the four standard NimbleGen internal controls. Successful enrichment of the capture libraries was estimated to range from a 6 to 9 of ΔCt value over the non-enriched samples.

<u>Sequencing</u>

Library templates were prepared for sequencing using Illumina's cBot cluster generation system with TruSeq PE Cluster Generation Kits. Briefly, these libraries were denatured with sodium hydroxide and diluted to 6-9 pM in hybridization buffer in order to achieve a load density of ~800K clusters/mm. Each library pool was loaded in a single lane of a HiSeq flow cell, and each lane was spiked with 1% phiX control library for run quality control. The sample libraries then underwent bridge amplification to form clonal clusters, followed by hybridization with the sequencing primer. Sequencing runs were performed in paired-end mode using the Illumina HiSeq 2000 platform. Using the TruSeq SBS Kits, sequencing-by-synthesis reactions were extended for 101 cycles from each end, with an additional 7 cycles for the index read. Sequencing runs generated approximately 300-400 million successful reads on each lane of a flow cell, yielding 7-14 Gb per sample. With these sequencing yields, samples achieved an average of 96% of the targeted exome bases covered to a depth of 20X or greater.

<u>WES data alignment</u>

Initial sequence analysis was performed using the HGSC Mercury analysis pipeline(Reid et al., 2014). First, the primary analysis software on the instrument produces .bcl files that are transferred off-instrument into the HGSC analysis infrastructure by the HiSeq Real-time Analysis module. Once the run is complete and all .bcl files are transferred, Mercury runs the vendor's primary analysis

software (CASAVA), which demultiplexes pooled samples and generates sequence reads and base-call confidence values (qualities). The next step is the mapping of reads to the GRCh37 Human reference genome (http://www.ncbi.nlm.nih.gov/projects/genome/assembly/grc/human/) using the Burrows- Wheeler aligner (BWA(Li and Durbin, 2009), http://bio-bwa.sourceforge.net/) and producing a BAM (binary alignment/map) file(Li et al., 2009). The third step involves quality recalibration (using GATK(DePristo et al., 2011), http://www.broadinstitute.org/gatk/), and where necessary the merging of separate sequence event BAMs into a single sample-level BAM. BAM sorting, duplicate read marking, and realignment to improve insertion and deletion (Indel) discovery all occur at this step.

Mutation calling

Mutation calling by multiple analysis centers was carried out, essentially as previously described (The Cancer Genome Atlas Research Network, 2013). Mutations were called using tumor and matched normal BAM files by the BCM HGSC, Broad Institute, University of California, Santa Cruz (UCSC), and British Colombia Genome Sequencing Center.

Strelka (Saunders et al., 2012)(v1.0.6) was used to identify somatic single nucleotide variants, and short insertions and deletions from the TCGA TGCT exome dataset. All parameters were set to defaults, with the exception of "isSkipDepthFilters", which was set to 1 in order to skip depth filtration given the higher coverage in exome datasets. 156 pairs of libraries were analyzed. When a blood sample was available, it served as the matched normal specimen; otherwise, the matched normal tissue was used. The variants were subsequently annotated using SnpEff (Cingolani et al., 2012)and the COSMIC (v61)(Forbes et al., 2009) and dbSNP (v137) (Smigielski et al., 2000)databases.

Single nucleotide somatic mutations were identified by RADIA (RNA AND DNA Integrated Analysis), a method that combines the patient matched normal and tumor DNA whole exome sequencing (DNA-WES) with the tumor RNA sequencing (RNA-Seq) for somatic mutation detection (software available at: https://github.com/aradenbaugh/radia/)(Radenbaugh et al., 2014). The inclusion of the RNA-Seq data in RADIA increases the power to detect somatic mutations, especially at low DNA allelic frequencies. By integrating the DNA and RNA, mutations that would be missed by traditional mutation calling algorithms that only examine the DNA can be rescued back. RADIA classifies somatic mutations into 3 categories depending on the read support from the DNA and RNA: 1) DNA calls – mutations that had high support in the DNA, 2) RNA Confirmation calls – mutations that had high support in both the DNA and RNA, 3) RNA Rescue calls – mutations that had high support in the RNA and weak support in the DNA. Here RADIA identified 6,894 DNA mutations, 908 RNA Confirmation mutations, and 366 RNA Rescue mutations.

Significantly mutated gene and mutational signature analysis

The statistical significance of mutation frequency in each gene was determined using the MutSigCV algorithm (v1.4)[Lawrence et al., 2013]. A p value of < 0.05 and a q value (false discovery rate) of < 0.1 were considered statistically significant. Mutation signature analysis was performed as previously described(Wang et al., 2015).

Mutation validation using NimbleGen capture array

Twenty-five frequently altered genes and five SNPs in functionally related genes were selected for mutation validation. A custom NimbleGen array (target size: ~200kb) was designed for mutation validation. All the samples including both tumors their matched control DNAs were sequenced. The basic Illumina library preparation protocol and the regents used were identical to that of WES except that for validation, a set of 96 molecular barcodes were used to allow high density multiplexing. The capture enrichment protocol was also same as described earlier for WES, with the following modifications: for capture, six pools of 43-48 libraries/pool were prepared and hybridized in solution to the custom capture design. Overnight hybridization at 56^0C was carried out instead of the 3-day hybridization at 47^0C as done for WES. Post enrichment steps were also similar: six enriched pools were pooled into three groups (86, 92, 92 libraries/pool) and sequenced on three lanes of HiSeq 2500. Except one control sample (TCGA-2G-AAKM-10A-01D-A433-10) had a library failure, all the samples were successfully sequenced. On an average, 298 Mb of uniquely mapped sequence data were generated across the target region and the targeted bases were covered to 599×.

Genes and sites selected for validation study.

Gene	Selection criteria	Design
KIT	Significantly mutated gene by MutSigCV	capture array#
KRAS	Significantly mutated gene by MutSigCV	capture array
NRAS	Significantly mutated gene by MutSigCV	capture array
PIK3CD	KIT/RAS/PI3K pathway, Recurrently mutated	capture array
PIK3CA	KIT/RAS/PI3K pathway, Recurrently mutated	capture array
PIK3CB	KIT/RAS/PI3K pathway, Recurrently mutated	capture array
PIK3C2G	KIT/RAS/PI3K pathway, Recurrently mutated	capture array
PIK3IP1	KIT/RAS/PI3K pathway, Recurrently mutated	capture array

SOS1	KIT/RAS/PI3K pathway, Recurrently mutated	capture array
NF1	Regulator of KIT/RAS/PI3K pathway, Recurrently mutated	capture array
PIK3C2A	KIT/RAS/PI3K pathway	capture array
PIK3C2B	KIT/RAS/PI3K pathway	capture array
PIK3R1	KIT/RAS/PI3K pathway	capture array
PIK3R5	KIT/RAS/PI3K pathway	capture array
BRAF	KIT/RAS/PI3K pathway	capture array
AKT3	KIT/RAS/PI3K pathway	capture array
MTOR	KIT/RAS/PI3K pathway	capture array
CBL	Regulator of KIT/RAS/PI3K pathway	capture array
JARID2	Frequently altered, with mutation, focal deletion and fusion, functionally important	capture array
CREBBP	Recurrently mutated and functionally important	capture array
SRCAP	Recurrently mutated, CREBBP activator	capture array
BIRC6	Recurrently mutated and functionally important	capture array
NUDC	Fusion partner of JARID2	capture array
rs995030	KITLG SNP	capture array
rs1508595	KITLG SNP	capture array
rs4590952	KITLG SNP	capture array
rs4624820	SPRY4 SNP	capture array
rs60180747	MAP2K1 SNP	capture array
CYTB	Recurrent mutation, mitochondrial gene, a mediator of FAS-induced apoptosis	LR-PCR*
RNR2	Recurrent mutation, mitochondrial RNA gene, suppress apoptosis	LR-PCR

#Capture array, NimbleGen targeted capture array; *LR-PCR: long-range PCR.

Mutation validation using LR-PCR

Mutations in two mitochondrial genes (CYTB and RNR2) were validated using long-range PCR (LR-PCR) approach. The mitochondrial DNA (mtDNA) were isolated from the tumor and matched control of 17 related TGCT cases, the LR-PCR, Illumina library construction and sequencing was performed using the same protocol as previously described(Davis et al., 2014). Briefly, the mtDNA products were successfully obtained from all 34 samples. A total of 500 ng final mtDNA LR-PCR product was used for Illumina paired-end library construction. All libraries were equally pooled and sequenced on one lane of HiSeq 2500. The sequencing reads were mapped to the mitochondrial reference genome (NC_012920.1 Mito Reference) using BWA(Li and Durbin, 2009), producing a BAM file(Li et al., 2009). The workflow of sequencing data processing and mutation calling was identical to that of WES.

Validation data analysis

A mutation is valid somatic if it was detected in the tumor sample but not in its matched control sample and passed all the quality filters as shown in the figure.

Mutation filtering and generation of final somatic MAF

To prepare the final somatic MAF for downstream integrative analysis, a stepwise filtering approach was applied to the multi-center MAF. First, the quality filters we applied and only those meeting the following criteria were selected: 1) the total read coverage in tumor and its matched control sample ≥5; 2) the variant read coverage in tumor ≥ 5 and the variant allele fraction (VAF) in tumor ≥ 0.05; 3) the ratio of VAF in normal and its tumor ≤ 0.15. Second, the germline filters were applied and any variants observed in the germline MAF file of the cohort were removed. Third, common SNPs were filtered out using dbSNP139 (MAF≤0.005). Then, all mutations left were visualized in Integrative Genomics Viewer (IGV, https://www.broadinstitute.org/igv/) and the mapping quality and uniqueness of the supporting reads was assessed using BLAT (https://genome.ucsc.edu/FAQ/FAQblat.html). The ones with ambiguous mapping and poor mapping quality were removed from the list. After that, the non-coding calls (at 3'UTR, 5'UTR, 3'Flank, 5'Flank, RNA, Intron, IGR) were removed. Finally, the validation data were added, the invalid calls, valid germline calls, and the single-center calls without validation data were removed. The valid mutations and cancer hotspot mutations are retained.

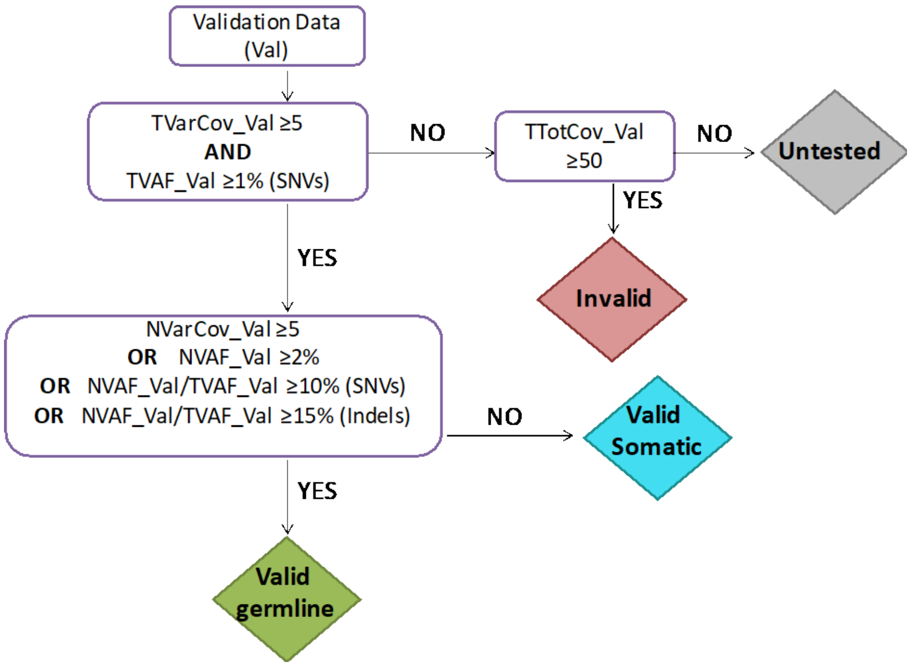

The workflow for validation analysis. Val, validation data; TVarCov, the tumor variant coverage; TVAF, the tumor variant allelic fraction; TTotCov, the tumor total coverage; NVarCov, the control variant coverage, NVAF, the control variant allelic fraction; NVAF_Val/TVAF_Val, the ratio of the variant allelic fraction in the matched control sample to that of the tumor sample.

Pathway Analysis

All sentinel loci established from eleven published germ-line GWAS of individuals with history of TGCT were evaluated. All genes within r2>0.8 linkage disequilibrium (LD) of the sentinel locus were included in analysis. Where more than one gene lay within the LD block and one gene contained a highly suggestive coding change in close LD with the sentinel risk variant, analysis of that gene was favored (e.g. *PMF1*). PubMed (http://www.ncbi.nih.gov/pubmed/), Online Mendelian Inheritance in Man (http://omim.org), and the Mouse Genome Informatics (http://www.informatics.jax.org/) databases were systematically searched using the gene name as primary search term to elicit primary references (April 2016). Where multiple historic names for the same gene were identified (e.g. *ZFP42/REX1*) each name was searched independently. Genes were assigned to pathways as stated by functional studies in the primary literature. All implicated pathways were included, including where multiple pathways were implicated.

Risk Alleles

Risk allele genotypes were determined by imputation for 36 SNPs in 128 cases in the cohort, and were assigned a risk burden value of 0 (homozygous for risk allele), 1 (heterozygous), or 2 (homozygous for non-risk allele).

Viral detection

The microbial database (v10) used for virus mapping was built by the CAMERA project (The Community Cyberinfrastructure for Advanced Marine Microbial Ecology Research and Analysis, http://camera.calit2.net) and was downloaded from its ftp site (ftp://portal.camera.calit2.net/ftp-links/cam_datasets/blastdb/). All unmapped reads (both paired and unpaired) were extracted from the full RNA-seq bam file. PCR or optical duplicates as well as anything that does not meet QC standards were removed and the remaining unmapped reads were converted to FASTA format. The unmapped reads were blasted against the microbial database to look for paired reads in which one read mapped to the human genome and the other mapped to the virus genome. Only unmapped reads in which had a significant blast hit to the virus genome and have the pair read mapped to the human genome were included for further analysis. The NDC (normalized depth coverage) was then calculated. NDC is the depth (total base pair) coverage normalized by the percent coverage, it is a combined scoring metric that takes into account the total number of mapped base pair to a given virus as well as the distribution of those mapped base pairs across that virus.

Filtering, annotation, and analysis of germline variants

The germline variants were filtered against the Phase-3 1000 Genome Project (http://phase3browser.1000genomes.org/Homo_sapiens/Info/Index), the NHLBI GO Exome Sequencing Project (ESP6500, http://evs.gs.washington.edu/EVS/), and the ExAc (the Exome Aggregation Consortium, http://exac.broadinstitute.org) and the variant with a population minor allele frequency greater than 0.5% were removed. The nonsynonymous variants including nonsense, missense, frame-shift variants and variants at splicing site were then selected for further analysis and the intronic mutations, mutations

at 3’ or 5’ UTR or UTR flanking regions, silent mutations, small in-frame insertions and deletions were excluded. To further evaluate the probability of a missense mutation being functionally deleterious, dbNSFP (v3.0) (Liu et al., 2016) was applied to add prediction scores for all missense mutations from twelve commonly used functional prediction algorithms: Polyphen-2 (Adzhubei et al., 2013), SIFT (Kumar et al., 2009), MutationTaster (Schwarz et al., 2014), Mutation Assessor (Reva et al., 2011), LRT (Chun and Fay, 2009), FATHMM-MKL (Shihab et al., 2015) and DANN (Quang et al., 2015), PROVEAN(Choi et al., 2012), WEST3 (Carter et al., 2013), CADD (Kircher et al., 2014), GERP++(Davydov et al., 2010), MetaSVM and MetaLR (Dong et al., 2015). A missense mutation that was called as “deleterious” or “damaging” by five or more algorithms were defined as a “deleterious” mutation. The genes with truncating or deleterious variants in two or more patients with documented 2nd primary tumors (n=9) were selected and the gene mutation frequencies were compared to patients without documented 2nd primary tumors (n=124) on the freeze set and the p-values were calculated using two-tailed Fisher’s Exact test.

Copy number analysis

SNP-based copy number analysis

DNA from each tumor or germline sample was hybridized to Affymetrix SNP 6.0 arrays using protocols at the Genome Analysis Platform of the Broad Institute as previously described(McCarroll et al., 2008). From raw .CEL files, Birdseed was used to infer a preliminary copy-number at each probe locus(Korn et al., 2008). For each tumor, genome-wide copy number estimates were refined using tangent normalization, in which tumor signal intensities are divided by signal intensities from the linear combination of all normal samples that are most similar to the tumor(Network, 2011) (and Tabak B. and Beroukhim R. Manuscript in preparation). This linear combination of normal samples tends to match the noise profile of the tumor better than any set of individual normal samples, thereby reducing the contribution of noise to the final copy-number profile. Individual copy-number estimates then underwent segmentation using Circular Binary Segmentation(Olshen et al., 2004). As part of this process of copy-number assessment and segmentation, regions corresponding to germline copy-number alterations were removed by applying filters generated from either the TCGA germline samples from the ovarian cancer analysis or from samples from this collection. Segmented copy number profiles for tumor and matched control DNAs were analyzed using Ziggurat Deconstruction, an algorithm that parsimoniously assigns a length and amplitude to the set of inferred copy-number changes underlying each segmented copy number profile(Mermel et al., 2011). Allelic copy number, whole genome doubling, subclonality, and purity and ploidy estimates were calculated using the ABSOLUTE algorithm(Carter et al., 2012). For samples with ABSOLUTE corrected copy number, CBS-derived segmented copy number values were re-centered using the *In Silico* Admixture Removal (ISAR) procedure(Zack et al., 2013). Significant focal copy number alterations across all tumors and within each histologic subtype were identified from ISAR-corrected segmented data using GISTIC 2.0.22(Mermel et al., 2011). For copy number based clustering, tumors were clustered based on total integer arm-level copy number as derived from the ABSOLUTE algorithm, normalized over four copies for tumors estimated to have been whole genome doubled once, and eight copies for tumors estimated to have been whole genome doubled twice. Clustering was done in R based on Manhattan distance using Ward's method. Timing of arm-level copy number alterations was inferred based on frequency of the event and level of aneuploidy in tumors containing the event relative to other events(Frigyesi et al., 2004). Assuming that level of aneuploidy increases overall during tumor progression, events associated with low levels of aneuploidy were predicted to occur early and events associated with high aneuploidy were predicted to occur late.

Inference of isochromosome 12p status

For samples with ABSOLUTE corrected copy number (as described above), integer segmental copy numbers were designated to allele a1 or a2 (minor or major alleles respectively). The modal copy number was recorded across each chromosome arm for both a1 and a2 (see “Before Inference” below). If the a1 (minor) allele copy number of chromosome arm 12p was equal to either the minor or major allele copy number of 12q, the specified 12q allele was inferred to correspond with a1; i.e., from the same homologous chromosome as the 12p a1 (minor) allele. If the difference between the a2 copy number of 12p and the a2 copy number of 12q was greater or equal to 2, the tumor was considered consistent with harboring an isochromosome 12p.

Tumor	TCGA-2G-AAFJ-01		
	chr 12 arm	modal a1	modal a2
Before Inference	p	2	5
	q	1	2

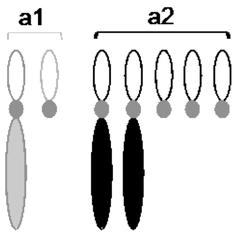

After Inference	p	2	5
	q	2	1

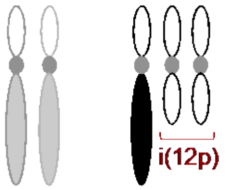

Methylation Analysis

Data processing

We performed bisulfite conversion on 1 µg of genomic DNA from 157 samples (including 150 primary, five secondary TGCT tumors and two technical replicates) using the EZ-96 DNA Methylation Kit (Zymo Research, Irvine, CA) according to the manufacturer's instructions. We assessed the amount of bisulfite converted DNA and completeness of bisulfite conversion using a panel of MethyLight-based quality control (QC) reactions as previously described(Campan et al., 2009). All bisulfite-converted DNA samples were then subject to repair using the Illumina Restoration Solution. The entire amount of each repaired sample entered the Infinium DNA methylation assay pipeline. Bisulfite-converted DNAs were whole genome amplified (WGA) and enzymatically-fragmented prior to hybridization to the Illumina Infinium HumanMethylation450 Beadchip array. BeadArrays were scanned using the Illumina iScan technology to produce IDAT files. Raw IDAT files for each sample were processed with the R/Bioconductor package *methylumi*.

Level 1 data contain raw IDAT files (two per sample) as produced by the iScan system and as mapped by the SDRF. These IDAT files can be directly processed by the R/Bioconductor package *methylumi*. Level 2 data contain background-corrected methylated (M) and unmethylated (U) summary intensities as extracted by the R/Bioconductor package *methylumi*. Non-detection probabilities (*P* values) were computed as the minimum of the two values (one per allele) for the empirical cumulative density function of the negative control probes in the appropriate color channel. Background correction was performed via normal-exponential deconvolution, as previously described(Triche et al., 2013). Multiple-batch archives have the intensities in each of the two channels multiplicatively scaled to match a reference sample (sample with R/G ratio of the normalization control probes closest to 1.0). Level 3 data contain β-value calculations with annotations for HGNC gene symbol, chromosome (UCSC hg19, Feb 2009), and genomic coordinate (UCSC hg19, Feb 2009) for each targeted CpG/CpH site on the array. Probes having a common SNP (Minor Allele Frequency > 0.01, per dbSNP build 135 via the UCSC snp135common track) within 10bp of the interrogated CpG site or having a 15bp from the interrogated CpG site which overlaps with a repetitive element (as defined by RepeatMasker and Tandem Repeat Finder Masks contained in the *BSgenome.Hsapiens.UCSC.hg19* R package) were masked as "NA" across all samples, and probes with a non-detection probability (*P* value) greater than 0.05 in a given sample were masked as "NA" on that array. Probes that were mapped to multiple sites on hg19 were annotated as "NA" for chromosome and 0 for CpG/CpH coordinate.

Unsupervised clustering

We downloaded Level 3 DNA methylation data from the TCGA data portal. For each CpG probe, we calculated the standard deviation (SD) of beta values across the 137 tumor samples in the final data freeze. Autosomal CpG probes (with probe IDs starting with 'cg') with an SD of greater than 0.26 (n=9,614) were used for clustering. The R function *hclust* was used for hierarchical clustering of these tumors with Ward's method, and the resulting dendrogram was cut at different levels with the R function *cutree* to evaluate cluster stability and biological relevance. When the tree is cut at k=2, seminomas and non-seminomas formed two major clusters. As illustrated in Figure S1, seminomas with different degrees of lymphocytic infiltration/contamination split from the main seminoma cluster, and non-seminomas further divided into two clusters, one with mixed components and low CpH methylation, and another primarily with embryonal components and relatively high CpH methylation at k=5. Therefore, we renamed the DNA methylation clusters to Sem-HC (seminomas with high contamination, Cluster 2), Sem-MC (seminomas with moderate contamination, Cluster 1), Sem-LC (seminomas with low contamination, Cluster 5), Mixed (Cluster 4) and CpH-high (Cluster 3). These names suggest group behavior and a sample in the CpH-high group could have low CpH methylation level.

Estimate of Lymphocyte Percentage and Subtraction of Lymphocyte DNA Contamination

We downloaded sorted blood dataset as previously published (GSE49618), and calculated mean DNA methylation beta values for lymphocytes (CD4 Tell, CD8 T cell, CD19 B cell) for each CpG locus. We correlated the DNA methylation levels observed in seminomas with different normal cell types generated throughout TCGA and in house and identified lymphocytes to be of the highest correlation. We identified 719 CpGs with high methylation level in lymphocytes (mean beta value >0.7) and low in non-seminomas (mean beta value <0.3) (Group 1 in Figure S1B). These probes represent a lymphocyte methylation signatures on an unmethylated testicular germ cell tumor background; therefore, we calculated the mode of kernel-smoothed DNA methylation distribution at these sites for each sample *j* as a surrogate for lymphocyte fraction for that sample, denoted as $\pi_{lym,j}$. 492 probes with low DNA methylation level in lymphocytes (mean beta value <0.3) and high DNA methylation level in non-seminomas (mean beta value >0.7) (Group 2 probes in Figure S1B) were used as a control, as seminomas were indistinguishable from lymphocytes at these loci and therefore lymphocytic infiltration would not influence the DNA methylation readouts in seminomas, but will dilute the high-level DNA methylation in non-seminomas. In contrast, no myeloid-specific DNA methylation fingerprint (Group 3 probes in Figure S1B) was observed. Pearson correlation coefficient between the calculated lymphocyte estimates with ABSOLUTE-derived tumor purity using 131 samples with estimates from both platforms was calculated with R and was determined to be -0.94.

Using a linear model, we can derive the corrected methylation (beta value) level for tumor sample *j* at locus *i*, denoted as $\beta'_{tum,i,j}$ using the following equation:

$$\beta'_{tum,i,j} = \frac{\beta_{tum,i,j} - \beta_{lym,i,j} * \pi_{lym,j}}{1 - \pi_{lym,j}}$$

Imprinted Loci

We downloaded the list of imprinted loci from a previously published study (Court et al., 2014), along with reference samples used in this study (GSE52578).

miRNA Sequencing Analysis

microRNA libraries and sequencing

We generated microRNA sequence (miRNA-seq) data for 133 TGCT tumor samples using methods described previously except that 1ug of total RNA (at 250ng/uL) was used as input instead of messenger RNA-depleted RNA (Chu et al., 2015). Briefly, reads were aligned to the GRCh37/hg19 reference human genome using BWA-MEM, and read count abundance was annotated with miRBase v16 stemloops and mature strands. While the read counts included only exact-match read alignments, .bam files at CGHub (cghub.ucsc.edu) (Wilks et al., 2014) include all sequence reads. We used miRBase v20 to assign 5p and 3p mature strand (miR) names to MIMAT accession IDs.

Unsupervised clustering of miRNA mature strands

To identify subtypes within the TGCT cohort we used hierarchical clustering with pheatmap v1.0.2 in R. The input was a reads-per-million (RPM) data matrix for the 303 (top 25%) miRBase v16 5p or 3p mature strands that had the largest variances across the cohort. We transformed each row of the matrix by $\log_{10}(RPM + 1)$, then used pheatmap to scale the rows. We used Ward.D2 for the clustering method with Pearson correlation and Euclidean as the distance measures for clustering the columns and rows respectively (Figure S3A).

Differentially abundant microRNAs

We identified miRs that were differentially abundant using unpaired two-class SAM analyses (samr v2.0) with an RPM input matrix and an FDR threshold of 0.05 (Li and Tibshirani, 2013). We compared pure seminoma (n=72) to samples with no seminoma component (n=61), pure seminoma (n=72) to pure embryonal (n=18), pure embryonal (n=18) to samples with no embryonal and no seminoma component (n=19), samples that only have a mature or immature teratoma component (n=6) to samples that have no mature or immature teratoma component (n=107), and pure seminomas with a KIT mutation (n=25) to pure seminomas without a KIT mutation (n=47) (Figure S3B, Table S4).

Random Forest

We ranked miRNAs from most important to least important for classifying samples into 3 groups: pure seminoms (n=72), pure or dominant embryonal (n=27), and pure or dominant yolk sac and teratoma (mature/immature) (n=29). The 9 samples that had no dominant histology were excluded from this analysis. To do this, we used a random forest classification software from Salford Systems, Inc and created 10,000 trees, each using 3 miRNAs as predictors (Figure S3C, Table S5).

mRNA Sequencing Analysis

RNA library construction, sequencing, and analysis

One μg of total RNA was converted to mRNA libraries using the lllumina mRNA TruSeq kit (RS-122-2001 or RS-122-2002) following the manufacturer's directions. Libraries were sequenced 48x7x48bp on the Illumina HiSeq 2000 as previously described (TCGA, 2012). FASTQ files were generated by CASAVA. RNA reads were aligned to the hg19 genome assembly using MapSplice 0.7.4 (Wang et al., 2010). Gene expression was quantified for the transcript models corresponding to the TCGA GAF2.1 (http://tcga-data.nci.nih.gov/docs/GAF/GAF.hg19.June2011.bundle/outputs/TCGA.hg19.June2011.gaf), using RSEM (Li and Dewey, 2011) and normalized within-sample to a fixed upper quartile. For further details on this processing, refer to Description file at the DCC data portal under the V2_MapSpliceRSEM workflow (https://tcga-data.nci.nih.gov/tcgafiles/ftp_auth/distro_ftpusers/anonymous/tumor/tgct/cgcc/unc.edu/illuminahiseq_rnaseqv2/rnaseqv2/unc.edu_TGCT.IlluminaHiSeq_RNASeqV2.mage-tab.1.0.0/DESCRIPTION.txt) or our alignment pipeline summary at CGHUB (https://cghub.ucsc.edu/docs/tcga/UNC_mRNAseq_summary.pdf).
Quantification of genes, transcripts, exons and junctions can be found at the TCGA Data Portal (https://tcga-data.nci.nih.gov/tcga/).

Expression data processing

Gene expression data was median centered and log2 transformed using the cluster 3.0 software(de Hoon et al., 2004). Missing data was imputed using Euclidean distance to find the nearest neighbor and calculate the missing value using the impute.knn R function(Hastie et al., 1999).

Subtype analysis

Seminoma subtypes were identified using consensus cluster plus(Wilkerson and Hayes, 2010). Gene selection criteria included genes with a standard deviation and overall log2 expression level greater than or equal to 1.5; yielding 1,504 genes for consensus clustering. Average silhouette widths were calculated using the R package 'cluster'(Rousseeuw, 1987). Principle components analysis was conducted and visualized in Matlab using the source code available(Hollern and Andrechek, 2014).

Pathway and Immune Module Analysis

Gene expression analysis of pathway and immune signatures was conducted by median centering samples to the median expression of each signature in R(Fan et al., 2011). Within each histology, samples were clustered based on expression of the immune signatures using the centroid linkage setting in Cluster 3.0(de Hoon et al., 2004). Gene signatures were compiled from individual manuscripts (Bindea et al., 2013; Fan et al., 2011; Gatza et al., 2010; Ghassabeh et al., 2006; Iglesia et al., 2014; Rody et al., 2009)and the broad institute's molecular signature database.

Testes Specific Antigens
The list of antigens used for analyses was derived from the CTdatabase(Almeida et al., 2009).

T-Cell Receptor and B-Cell Receptor Diversity
T-cell receptor diversity was calculated using MixCr(Bolotin et al., 2015; Bolotin et al., 2013). MiXCR is available at http://mixcr.milaboratory.com/ and https://github.com/milaboratory/mixcr/.

B-cell receptor diversity was calculated using V'DJer(Mose et al., 2016). V'DJer is implemented in C/C ++, free for academic use and can be downloaded from Github: https://github.com/mozack/vdjer.

Box and X-Y Correlation Plots
Correlation and box plots were built in R Studio. The R value was calculated using pearson correlation and the cor.test function in R.

RPPA Analysis
Protein extraction and data processing
Protein was extracted using RPPA lysis buffer (1% Triton X-100, 50 mmol/L Hepes (pH 7.4), 150 mmol/L NaCl, 1.5 mmol/L MgCl2, 1 mmol/L EGTA, 100 mmol/L NaF, 10 mmol/L NaPPi, 10% glycerol, 1 mmol/L phenylmethylsulfonyl fluoride, 1 mmol/L Na3VO4, and aprotinin 10 ug/mL) from human tumors and RPPA was performed as described previously (Coombes et al., 2009; Hennessy et al., 2007; Hu et al., 2007; Liang et al., 2007; Tibes et al., 2006). Lysis buffer was used to lyse frozen tumors by Precellys homogenization. Tumor lysates were adjusted to 1 µg/µL concentration as assessed by bicinchoninic acid assay (BCA) and boiled with 1% SDS. Tumor lysates were manually serial diluted in two-fold of 5 dilutions with lysis buffer. An Aushon Biosystems 2470 arrayer (Burlington, MA) printed 1,056 samples on nitrocellulose-coated slides (Grace Bio-Labs). Slides were probed with 218 validated primary antibodies followed by corresponding secondary antibodies (Goat anti-Rabbit IgG, Goat anti-Mouse IgG or Rabbit anti-Goat IgG). Signal was captured using a DakoCytomation-catalyzed system and DAB colorimetric reaction. Slides were scanned in a CanoScan 9000F. Spot intensities were analyzed and quantified using Array-Pro Analyzer (Media Cybernetics Washington DC) to generate spot signal intensities (Level 1 data). The software SuperCurveGUI (Coombes et al., 2009; Hu et al., 2007), available at http://bioinformatics.mdanderson.org/Software/supercurve/, was used to estimate the EC50 values of the proteins in each dilution series (in log2 scale). Briefly, a fitted curve ("supercurve") was plotted with the signal intensities on the Y-axis and the relative log2 concentration of each protein on the X-axis using the non-parametric, monotone increasing B-spline model(Tibes et al., 2006). During the process, the raw spot intensity data were adjusted to correct spatial bias before model fitting. A QC metric (Coombes et al., 2009) was returned for each slide to help determine the quality of the slide: if the score is less than 0.8 on a 0-1 scale, the slide was dropped. In most cases, the staining was repeated to obtain a high quality score. If more than one slide was stained for an antibody, the slide with the highest QC score was used for analysis (Level 2 data). Protein measurements were corrected for loading as described [3,5,6] using median centering across antibodies (level 3 data). In total, 218 antibodies and 104 Testicular Germ Cell Tumor (TGCT) samples were used for the analysis, including 51 seminoma, 20 embryonal carcinoma, 19 non-seminoma (mixed), 6 yolk sac tumors, 6 mature teratomas, and 2 immature teratomas. Final selection of antibodies was also driven by the availability of high quality antibodies that consistently pass a strict validation process as previously described(Hennessy et al., 2010). These antibodies are assessed for specificity, quantification and sensitivity (dynamic range) in their application for protein extracts from cultured cells or tumor tissue. Antibodies are labeled as validated and use with caution based on degree of validation by criteria previously described (Hennessy et al., 2010).

RPPA arrays were quantitated and processed (including normalization and load controlling) as described previously, using MicroVigene (VigeneTech, Inc., Carlisle, MA) and the R package SuperCurve (version-1.3), available at http://bioinformatics.mdanderson.org/OOMPA (Hu et al., 2007; Tibes et al., 2006). Raw data (level 1), SuperCurve nonparameteric model fitting on a single array (level 2), and loading corrected data (level 3) were deposited at the DCC.

Data normalization
Data was normalized by median centering across all the antibodies for each sample to correct for sample loading differences.

RPPA subtypes
RPPA subtypes were derived using consensus clustering(Monti et al., 2003). Pearson correlation was used as distance metric and Ward was used as a linkage algorithm in the clustering analysis. A total of 104 samples and 218 antibodies were used in the analysis.

Pathway analysis

To illustrate the role of cell signaling network in TGCT, we calculated twelve pathway scores based on a previously described method (Akbani et al., 2014).

Cross Platform analysis

Neoantigen predictions

TCGA Testicular Germ Cell Tumor samples with available mRNA-sequencing data, exome-sequencing data, and tumor-specific mutation annotation data were used as inputs for the neoantigen prediction pipeline. The Neoantigen prediction bioinformatics pipeline was similar to that developed by Rajasagi et al. (Rajasagi et al., 2014). Pysam was used to determine RNA-seq read support of missense mutations. Eight-, Nine-, Ten-, and eleven-mer peptides derived from 3 ORFs with all possible combinations of missense mutations that overlap the genomic location of peptide in the ENCODE reference transcript set were generated by the in-silico peptide generation pipeline. Peptide sequences with internal stop codon(s) were discarded by the in-silico peptide generation pipeline. The expression level of each peptide was inferred by the lowest missense mutation RNA-seq read support. HLA class I (HLA-A, HLA-B, HLA-C) type of each tumor sample was identified using PHLAT (Bai et al., 2014). Binding affinity of peptides to HLA molecules expressed by the tumor was predicted using NetMHCpan (version 2.8). Binding affinity of peptides to null alleles, alternatively expressed alleles, and alleles not supported by NetMHCpan were not predicted. Neoantigens were considered high-affinity for IC50 values of less than or equal to 50nM, moderate-affinity for IC50 values of less than or equal to 150nM, and weak-affinity neoantigen for IC50 values of less than or equal to 500nM. Peptides were filtered by their binding affinities (IC50 nM) to class I alleles in the tumor sample's HLA type and RNA expression level of the predicted source transcript(s). Peptides with an IC50 value of less than or equal to 500 nM for at least 1 class I allele and RNA read support of at least 2 reads were considered predicted neoantigens.

Tumor Map

The Tumor Map is a published method (Newton et al., 2017) and represents a dimensionality reduction and visualization method for high dimensional genomic data (Ceccarelli et al., 2016; Farshidfar et al., 2017). It allows viewing and browsing relationships between high dimensional heterogeneous genomic samples in a two-dimensional map, analogous to exploring geographical maps in the Google Maps web application. Samples are arranged in a two-dimensional space and then associated to hexagons in a regular hexagonal grid. The relative distances in the map approximate the relative similarities between the samples in the original high-dimensional genomic space. Samples with similar genomic profiles should be placed near each other in the map. Samples that are less similar should be farther away from each other. Given such relations are preserved, clusters of samples that appear as "islands" in the map will indicate groups of samples that share genomic and/or epigenomic events.

To build multi-platform map, we combined tumor mRNA expression, copy number variation, and methylation profiles for Testicular Germ Cell Tumors (TGCT). We used mRNA expression data from RNA-seq (n = 137), copy number Gistic calls (n = 137), and methylation profiles from HumanMethylation450 (HM450) platform (n = 137). We computed sample-by-sample pair-wise similarities for each platform, producing three square similarity matrices. We used Spearman rank correlation (Spearman, 1904) as a similarity measure on these continuous-valued datasets (mRNA expression, copy number variation, and methylation). Next, we standardized each similarity matrix using the Context Likelihood of Relatedness (CLR) approach (Faith et al., 2007).Given a similarity matrix as input, this method outputs a set of *relative similarities*, where each similarity pair reflects how similar the two samples are to each other compared to how similar each is to *any sample* in a particular cohort. More formally, we compute the standardized similarity measure $Z_D(i,j)$ between two samples i and j from the dth dataset:

$$Z_d(i,j) = \frac{1}{2}\left(\frac{S_d(i,j) - m_d(i)}{\sqrt{v_d(i)}} + \frac{S_d(i,j) - m_d(j)}{\sqrt{v_d(j)}} \right),$$

where $m_d(k)$ is the mean and $v_d(k)$ is the variance of the similarities to a particular sample k in dataset $\underline{d}$. We compute integrated similarity measures Z^* from the relative Z-scores across D different data platforms as:

$$Z^*(i,j) = \frac{\sum_{d=1}^{D} I(d,i) \cdot I(d,j) \cdot Z_d(i,j)}{\sum_{d=1}^{D} I(d,i) \cdot I(d,j)},$$

where $I(d,k)$ records whether sample k has data in dataset d. Thus, Z^* represents a simple averaging of the relative similarities between two samples across only those datasets for which both of the samples have valid (non-missing) observations. We note that Z^* could incorporate weightings for each dataset w_d indicating the importance of each of the D platforms being combined. However, we did not explore this option for the analysis presented here. The resulting standardized similarity matrix is a square samples-by-samples matrix where the samples are the union of all samples represented in the platforms.

To build the map layout, the closest neighborhood of 10 samples was selected for each sample from the standardized integrated similarity matrix. We represent the local neighborhoods as a graph where the nodes are the samples and an edge links any two samples if one of them is in the top 10 neighbors of the other. The magnitude of the similarity was used as the edge weight. An X-Y position in the two-dimensional plane was calculated from the graph using a spring-embedded graph layout (Golbeck and Mutton, 2005) algorithm implemented in the Distributed Recursive Graph Layout toolbox (Martin et al., 2011).The spring-embedded layout algorithm treats edges as springs and allows the springs to oscillate for a fixed amount of time with the energy inversely proportional to the edge weights. Under these conditions, springs with large weights do not oscillate much, causing those vertices to stay together. However, springs with small weights oscillate more and end up farther away from each other. This method allows constructing a two-dimensional spatial layout of the graph with clusters of samples forming clique-like hub sub-structures. Our method then associates each of the nodes with a fixed location a two-dimensional hexagonal grid. Each hexagon-shaped cell in the grid can be assigned no more than one vertex, and some can be assigned none representing "empty space" in the map. If multiple vertices contest for the same grid cell, a random vertex selection is made and placed into the cell; and the other competing vertices are assigned to neighboring empty cells using a greedy strategy, snapping around the original cell in a spiral-like manner.

PARADIGM Analysis

We applied PARADIGM (Vaske et al., 2010) method to the TGCT RNASeq mRNA expression (n=137) and copy number Gistic calls (n=137) data. PARADIGM is a computational inference method for activity levels of molecular entities (e.g. gene products, complexes, etc.) in the cell based on the mRNA levels, copy number, and molecular pathway relationships information. PARADIGM output for this TGCT cohort consists of 21,545 inferred pathway levels (IPLs). We restricted the IPLs to only those IPLs that had more than 3% of samples in the cohort with significant levels over the randomized background model, resulting in 7,617 significant IPLs.

For unsupervised analysis of TGCT cohort based on PARADIGM IPLs, we selected the top 3,000 most varying IPLs and performed unsupervised consensus k-means clustering, using complete linkage. We identified, using silhouette method, that the best solution contains three (k=3) clusters. These clusters are driven by tumor histology, consisting of seminoma tumors, embryonal non-seminoma tumors, and other non-seminoma tumors respectively.

For IPL enrichment analysis, we further restricted the 7,617 IPLs to only protein-coding IPLs, resulting in 1,477 IPLs. We ordered the samples (n=137) in the cohort by the histology groups and applied k-means clustering to the final set of IPLs, resulting in a solution of seven (k=7) major clusters. We annotated these clusters by performing gene set enrichment analysis on the IPLs in each cluster using MSigDB (Liberzon et al., 2011)molecular pathway database.

Supplemental References

Adzhubei, I., Jordan, D. M., and Sunyaev, S. R. (2013). Predicting functional effect of human missense mutations using PolyPhen-2. Curr Protoc Hum Genet *Chapter 7*, Unit7 20.

Akbani, R., Ng, P. K. S., Werner, H. M., Shahmoradgoli, M., Zhang, F., Ju, Z., Liu, W., Yang, J.-Y., Yoshihara, K., and Li, J. (2014). A pan-cancer proteomic perspective on The Cancer Genome Atlas. Nature communications *5*.

Almeida, L. G., Sakabe, N. J., Silva, M. C. C., Mundstein, A. S., Cohen, T., Chen, Y.-T., Chua, R., Gurung, S., Gnjatic, S., and Jungbluth, A. A. (2009). CTdatabase: a knowledge-base of high-throughput and curated data on cancer-testis antigens. Nucleic acids research *37*, D816-D819.

Bai, Y., Ni, M., Cooper, B., Wei, Y., and Fury, W. (2014). Inference of high resolution HLA types using genome-wide RNA or DNA sequencing reads. BMC genomics *15*, 325.

Bainbridge, M. N., Wang, M., Wu, Y., Newsham, I., Muzny, D. M., Jefferies, J. L., Albert, T. J., Burgess, D. L., and Gibbs, R. A. (2011). Targeted enrichment beyond the consensus coding DNA sequence exome reveals exons with higher variant densities. Genome Biol *12*, R68.

Bindea, G., Mlecnik, B., Tosolini, M., Kirilovsky, A., Waldner, M., Obenauf, A. C., Angell, H., Fredriksen, T., Lafontaine, L., and Berger, A. (2013). Spatiotemporal dynamics of intratumoral immune cells reveal the immune landscape in human cancer. Immunity *39*, 782-795.

Bolotin, D. A., Poslavsky, S., Mitrophanov, I., Shugay, M., Mamedov, I. Z., Putintseva, E. V., and Chudakov, D. M. (2015). MiXCR: software for comprehensive adaptive immunity profiling. Nature methods *12*, 380-381.

Bolotin, D. A., Shugay, M., Mamedov, I. Z., Putintseva, E. V., Turchaninova, M. A., Zvyagin, I. V., Britanova, O. V., and Chudakov, D. M. (2013). MiTCR: software for T-cell receptor sequencing data analysis. Nature methods *10*, 813-814.

Campan, M., Weisenberger, D. J., Trinh, B., and Laird, P. W. (2009). MethyLight. Methods Mol Biol *507*, 325-337.

Carter, H., Douville, C., Stenson, P. D., Cooper, D. N., and Karchin, R. (2013). Identifying Mendelian disease genes with the variant effect scoring tool. BMC Genomics *14 Suppl 3*, S3.

Carter, S. L., Cibulskis, K., Helman, E., McKenna, A., Shen, H., Zack, T., Laird, P. W., Onofrio, R. C., Winckler, W., and Weir, B. A. (2012). Absolute quantification of somatic DNA alterations in human cancer. Nature biotechnology *30*, 413-421.

Ceccarelli, M., Barthel, F. P., Malta, T. M., Sabedot, T. S., Salama, S. R., Murray, B. A., Morozova, O., Newton, Y., Radenbaugh, A., Pagnotta, S. M., *et al.* (2016). Molecular Profiling Reveals Biologically Discrete Subsets and Pathways of Progression in Diffuse Glioma. Cell *164*, 550-563.

Choi, Y., Sims, G. E., Murphy, S., Miller, J. R., and Chan, A. P. (2012). Predicting the functional effect of amino acid substitutions and indels. PLoS One *7*, e46688.

Chu, A., Robertson, G., Brooks, D., Mungall, A. J., Birol, I., Coope, R., Ma, Y., Jones, S., and Marra, M. A. (2015). Large-scale profiling of microRNAs for The Cancer Genome Atlas. Nucleic Acids Res.

Chun, S., and Fay, J. C. (2009). Identification of deleterious mutations within three human genomes. Genome Res *19*, 1553-1561.

Cingolani, P., Platts, A., Wang, L. L., Coon, M., Nguyen, T., Wang, L., Land, S. J., Lu, X., and Ruden, D. M. (2012). A program for annotating and predicting the effects of single nucleotide polymorphisms, SnpEff: SNPs in the genome of Drosophila melanogaster strain w1118; iso-2; iso-3. Fly *6*, 80-92.

Coombes, K., Neeley, E., and Joy, C. (2009). SuperCurve: SuperCurve Package. R package version *1*.

Court, F., Tayama, C., Romanelli, V., Martin-Trujillo, A., Iglesias-Platas, I., Okamura, K., Sugahara, N., Simon, C., Moore, H., Harness, J. V., *et al.* (2014). Genome-wide parent-of-origin DNA methylation analysis reveals the intricacies of human imprinting and suggests a germline methylation-independent mechanism of establishment. Genome research *24*, 554-569.

Davis, C. F., Ricketts, C. J., Wang, M., Yang, L., Cherniack, A. D., Shen, H., Buhay, C., Kang, H., Kim, S. C., Fahey, C. C., *et al.* (2014). The somatic genomic landscape of chromophobe renal cell carcinoma. Cancer Cell *26*, 319-330.

Davydov, E. V., Goode, D. L., Sirota, M., Cooper, G. M., Sidow, A., and Batzoglou, S. (2010). Identifying a high fraction of the human genome to be under selective constraint using GERP++. PLoS computational biology *6*, e1001025.

de Hoon, M. J., Imoto, S., Nolan, J., and Miyano, S. (2004). Open source clustering software. Bioinformatics *20*, 1453-1454.

DePristo, M. A., Banks, E., Poplin, R., Garimella, K. V., Maguire, J. R., Hartl, C., Philippakis, A. A., del Angel, G., Rivas, M. A., Hanna, M., *et al.* (2011). A framework for variation discovery and genotyping using next-generation DNA sequencing data. Nat Genet *43*, 491-498.

Dong, C., Wei, P., Jian, X., Gibbs, R., Boerwinkle, E., Wang, K., and Liu, X. (2015). Comparison and integration of deleteriousness prediction methods for nonsynonymous SNVs in whole exome sequencing studies. Hum Mol Genet *24*, 2125-2137.

Faith, J. J., Hayete, B., Thaden, J. T., Mogno, I., Wierzbowski, J., Cottarel, G., Kasif, S., Collins, J. J., and Gardner, T. S. (2007). Large-scale mapping and validation of Escherichia coli transcriptional regulation from a compendium of expression profiles. PLoS biol *5*, e8.

Fan, C., Prat, A., Parker, J. S., Liu, Y., Carey, L. A., Troester, M. A., and Perou, C. M. (2011). Building prognostic models for breast cancer patients using clinical variables and hundreds of gene expression signatures. BMC medical genomics *4*, 1.

Farshidfar, F., Zheng, S., Gingras, M. C., Newton, Y., Shih, J., Robertson, A. G., Hinoue, T., Hoadley, K. A., Gibb, E. A., Roszik, J., *et al.* (2017). Integrative Genomic Analysis of Cholangiocarcinoma Identifies Distinct IDH-Mutant Molecular Profiles. Cell reports *18*, 2780-2794.

Forbes, S. A., Tang, G., Bindal, N., Bamford, S., Dawson, E., Cole, C., Kok, C. Y., Jia, M., Ewing, R., and Menzies, A. (2009). COSMIC (the Catalogue of Somatic Mutations in Cancer): a resource to investigate acquired mutations in human cancer. Nucleic acids research, gkp995.

Frigyesi, A., Gisselsson, D., Hansen, G. B., Soller, M., Mitelman, F., and Höglund, M. (2004). A model for karyotypic evolution in testicular germ cell tumors. Genes, Chromosomes and Cancer *40*, 172-178.

Gatza, M. L., Lucas, J. E., Barry, W. T., Kim, J. W., Wang, Q., Crawford, M. D., Datto, M. B., Kelley, M., Mathey-Prevot, B., and Potti, A. (2010). A pathway-based classification of human breast cancer. Proceedings of the National Academy of Sciences *107*, 6994-6999.

Ghassabeh, G. H., De Baetselier, P., Brys, L., Noël, W., Van Ginderachter, J. A., Meerschaut, S., Beschin, A., Brombacher, F., and Raes, G. (2006). Identification of a common gene signature for type II cytokine–associated myeloid cells elicited in vivo in different pathologic conditions. Blood *108*, 575-583.

Golbeck, J., and Mutton, P. (2005). Spring-Embedded graphs for semantic visualization. Visualizing the Semantic Web, 172-182.

Hastie, T., Tibshirani, R., Sherlock, G., Eisen, M., Brown, P., and Botstein, D. (1999). Imputing missing data for gene expression arrays. In, (Stanford University Statistics Department Technical report).

Hennessy, B. T., Lu, Y., Gonzalez-Angulo, A. M., Carey, M. S., Myhre, S., Ju, Z., Davies, M. A., Liu, W., Coombes, K., and Meric-Bernstam, F. (2010). A technical assessment of the utility of reverse phase protein arrays for the study of the functional proteome in non-microdissected human breast cancers. Clinical proteomics *6*, 129.

Hennessy, B. T., Lu, Y., Poradosu, E., Yu, Q., Yu, S., Hall, H., Carey, M. S., Ravoori, M., Gonzalez-Angulo, A. M., and Birch, R. (2007). Pharmacodynamic markers of perifosine efficacy. Clinical cancer research *13*, 7421-7431.

Hollern, D. P., and Andrechek, E. R. (2014). A genomic analysis of mouse models of breast cancer reveals molecular features of mouse models and relationships to human breast cancer. Breast Cancer Res *16*.

Hu, J., He, X., Baggerly, K. A., Coombes, K. R., Hennessy, B. T., and Mills, G. B. (2007). Non-parametric quantification of protein lysate arrays. Bioinformatics *23*, 1986-1994.

Iglesia, M. D., Vincent, B. G., Parker, J. S., Hoadley, K. A., Carey, L. A., Perou, C. M., and Serody, J. S. (2014). Prognostic B-cell signatures using mRNA-seq in patients with subtype-specific breast and ovarian cancer. Clinical Cancer Research *20*, 3818-3829.

Kircher, M., Witten, D. M., Jain, P., O'Roak, B. J., Cooper, G. M., and Shendure, J. (2014). A general framework for estimating the relative pathogenicity of human genetic variants. Nat Genet *46*, 310-315.

Korn, J. M., Kuruvilla, F. G., McCarroll, S. A., Wysoker, A., Nemesh, J., Cawley, S., Hubbell, E., Veitch, J., Collins, P. J., and Darvishi, K. (2008). Integrated genotype calling and association analysis of SNPs, common copy number polymorphisms and rare CNVs. Nature genetics *40*, 1253-1260.

Kumar, P., Henikoff, S., and Ng, P. C. (2009). Predicting the effects of coding non-synonymous variants on protein function using the SIFT algorithm. Nat Protoc *4*, 1073-1081.

Lawrence, M. S., Stojanov, P., Polak, P., Kryukov, G. V., Cibulskis, K., Sivachenko, A., Carter, S. L., Stewart, C., Mermel, C. H., Roberts, S. A., *et al.* (2013). Mutational heterogeneity in cancer and the search for new cancer-associated genes. Nature *499*, 214-218.

Li, B., and Dewey, C. N. (2011). RSEM: accurate transcript quantification from RNA-Seq data with or without a reference genome. BMC bioinformatics *12*, 323.

Li, H., and Durbin, R. (2009). Fast and accurate short read alignment with Burrows-Wheeler transform. Bioinformatics *25*, 1754-1760.

Li, H., Handsaker, B., Wysoker, A., Fennell, T., Ruan, J., Homer, N., Marth, G., Abecasis, G., Durbin, R., and Genome Project Data Processing, S. (2009). The Sequence Alignment/Map format and SAMtools. Bioinformatics *25*, 2078-2079.

Li, J., and Tibshirani, R. (2013). Finding consistent patterns: a nonparametric approach for identifying differential expression in RNA-Seq data. Stat Methods Med Res *22*, 519-536.

Liang, J., Shao, S. H., Xu, Z.-X., Hennessy, B., Ding, Z., Larrea, M., Kondo, S., Dumont, D. J., Gutterman, J. U., and Walker, C. L. (2007). The energy sensing LKB1–AMPK pathway regulates p27kip1 phosphorylation mediating the decision to enter autophagy or apoptosis. Nature cell biology *9*, 218-224.

Liberzon, A., Subramanian, A., Pinchback, R., Thorvaldsdottir, H., Tamayo, P., and Mesirov, J. P. (2011). Molecular signatures database (MSigDB) 3.0. Bioinformatics *27*, 1739-1740.

Liu, X., Wu, C., Li, C., and Boerwinkle, E. (2016). dbNSFP v3.0: A One-Stop Database of Functional Predictions and Annotations for Human Nonsynonymous and Splice-Site SNVs. Hum Mutat *37*, 235-241.

Martin, S., Brown, W. M., Klavans, R., and Boyack, K. W. (2011). OpenOrd: an open-source toolbox for large graph layout. Paper presented at: IS&T/SPIE Electronic Imaging (International Society for Optics and Photonics).

McCarroll, S. A., Kuruvilla, F. G., Korn, J. M., Cawley, S., Nemesh, J., Wysoker, A., Shapero, M. H., de Bakker, P. I., Maller, J. B., and Kirby, A. (2008). Integrated detection and population-genetic analysis of SNPs and copy number variation. Nature genetics *40*, 1166-1174.

Mermel, C. H., Schumacher, S. E., Hill, B., Meyerson, M. L., Beroukhim, R., and Getz, G. (2011). GISTIC2. 0 facilitates sensitive and confident localization of the targets of focal somatic copy-number alteration in human cancers. Genome biology *12*, 1.

Monti, S., Tamayo, P., Mesirov, J., and Golub, T. (2003). Consensus clustering: a resampling-based method for class discovery and visualization of gene expression microarray data. Machine learning *52*, 91-118.

Mose, L. E., Selitsky, S. R., Bixby, L. M., Marron, D. L., Iglesia, M. D., Serody, J. S., Perou, C. M., Vincent, B. G., and Parker, J. S. (2016). Assembly-based inference of B-cell receptor repertoires from short read RNA sequencing data with V'DJer. Bioinformatics, btw526.

Network, C. G. A. R. (2011). Integrated genomic analyses of ovarian carcinoma. Nature *474*, 609-615.
Newton, Y., Novak, A. M., Swatloski, T., McColl, D. C., Chopra, S., Graim, K., Weinstein, A. S., Baertsch, R., Salama, S. R., and Ellrott, K. (2017). TumorMap: Exploring the Molecular Similarities of Cancer Samples in an Interactive Portal. Cancer research *77*, e111-e114.
Olshen, A. B., Venkatraman, E., Lucito, R., and Wigler, M. (2004). Circular binary segmentation for the analysis of array-based DNA copy number data. Biostatistics *5*, 557-572.
Quang, D., Chen, Y., and Xie, X. (2015). DANN: a deep learning approach for annotating the pathogenicity of genetic variants. Bioinformatics *31*, 761-763.
Radenbaugh, A. J., Ma, S., Ewing, A., Stuart, J. M., Collisson, E. A., Zhu, J., and Haussler, D. (2014). RADIA: RNA and DNA integrated analysis for somatic mutation detection. PloS one *9*, e111516.
Rajasagi, M., Shukla, S. A., Fritsch, E. F., Keskin, D. B., DeLuca, D., Carmona, E., Zhang, W., Sougnez, C., Cibulskis, K., Sidney, J., *et al.* (2014). Systematic identification of personal tumor-specific neoantigens in chronic lymphocytic leukemia. Blood *124*, 453-462.
Reid, J. G., Carroll, A., Veeraraghavan, N., Dahdouli, M., Sundquist, A., English, A., Bainbridge, M., White, S., Salerno, W., Buhay, C., *et al.* (2014). Launching genomics into the cloud: deployment of Mercury, a next generation sequence analysis pipeline. BMC Bioinformatics *15*, 30.
Reva, B., Antipin, Y., and Sander, C. (2011). Predicting the functional impact of protein mutations: application to cancer genomics. Nucleic Acids Res *39*, e118.
Rody, A., Holtrich, U., Pusztai, L., Liedtke, C., Gaetje, R., Ruckhaeberle, E., Solbach, C., Hanker, L., Ahr, A., and Metzler, D. (2009). T-cell metagene predicts a favorable prognosis in estrogen receptor-negative and HER2-positive breast cancers. Breast Cancer Research *11*, 1.
Rousseeuw, P. J. (1987). Silhouettes: a graphical aid to the interpretation and validation of cluster analysis. Journal of computational and applied mathematics *20*, 53-65.
Saunders, C. T., Wong, W. S., Swamy, S., Becq, J., Murray, L. J., and Cheetham, R. K. (2012). Strelka: accurate somatic small-variant calling from sequenced tumor–normal sample pairs. Bioinformatics *28*, 1811-1817.
Schwarz, J. M., Cooper, D. N., Schuelke, M., and Seelow, D. (2014). MutationTaster2: mutation prediction for the deep-sequencing age. Nat Methods *11*, 361-362.
Shihab, H. A., Rogers, M. F., Gough, J., Mort, M., Cooper, D. N., Day, I. N., Gaunt, T. R., and Campbell, C. (2015). An integrative approach to predicting the functional effects of non-coding and coding sequence variation. Bioinformatics *31*, 1536-1543.
Smigielski, E. M., Sirotkin, K., Ward, M., and Sherry, S. T. (2000). dbSNP: a database of single nucleotide polymorphisms. Nucleic acids research *28*, 352-355.
Spearman, C. (1904). The proof and measurement of association between two things. The American journal of psychology *15*, 72-101.
Tibes, R., Qiu, Y., Lu, Y., Hennessy, B., Andreeff, M., Mills, G. B., and Kornblau, S. M. (2006). Reverse phase protein array: validation of a novel proteomic technology and utility for analysis of primary leukemia specimens and hematopoietic stem cells. Molecular cancer therapeutics *5*, 2512-2521.
Triche, T. J., Jr., Weisenberger, D. J., Van Den Berg, D., Laird, P. W., and Siegmund, K. D. (2013). Low-level processing of Illumina Infinium DNA Methylation BeadArrays. Nucleic Acids Res *41*, e90.
Vaske, C. J., Benz, S. C., Sanborn, J. Z., Earl, D., Szeto, C., Zhu, J., Haussler, D., and Stuart, J. M. (2010). Inference of patient-specific pathway activities from multi-dimensional cancer genomics data using PARADIGM. Bioinformatics *26*, i237-i245.
Wang, K., Singh, D., Zeng, Z., Coleman, S. J., Huang, Y., Savich, G. L., He, X., Mieczkowski, P., Grimm, S. A., Perou, C. M., *et al.* (2010). MapSplice: accurate mapping of RNA-seq reads for splice junction discovery. Nucleic acids research *38*, e178.
Wang, L., Ni, X., Covington, K. R., Yang, B. Y., Shiu, J., Zhang, X., Xi, L., Meng, Q., Langridge, T., Drummond, J., *et al.* (2015). Genomic profiling of Sezary syndrome identifies alterations of key T cell signaling and differentiation genes. Nat Genet *47*, 1426-1434.
Wilkerson, M. D., and Hayes, D. N. (2010). ConsensusClusterPlus: a class discovery tool with confidence assessments and item tracking. Bioinformatics *26*, 1572-1573.
Wilks, C., Cline, M. S., Weiler, E., Diehkans, M., Craft, B., Martin, C., Murphy, D., Pierce, H., Black, J., Nelson, D., *et al.* (2014). The Cancer Genomics Hub (CGHub): overcoming cancer through the power of torrential data. Database (Oxford) *2014*.
Zack, T. I., Schumacher, S. E., Carter, S. L., Cherniack, A. D., Saksena, G., Tabak, B., Lawrence, M. S., Zhang, C.-Z., Wala, J., and Mermel, C. H. (2013). Pan-cancer patterns of somatic copy number alteration. Nature genetics *45*, 1134-1140.
