# Supplementary material for: Integrated Molecular Characterization of Testicular Germ Cell Tumors
Source: Cell Rep. Author manuscript; Available in PMC 2018 Aug 3. (PMC6075738; doi:10.1016/j.celrep.2018.05.039)
Supplement: 8 [file NIHMS977848-supplement-8.pdf]

Cell Reports

Resource

Integrated Molecular Characterization of Testicular Germ Cell Tumors

Graphical Abstract

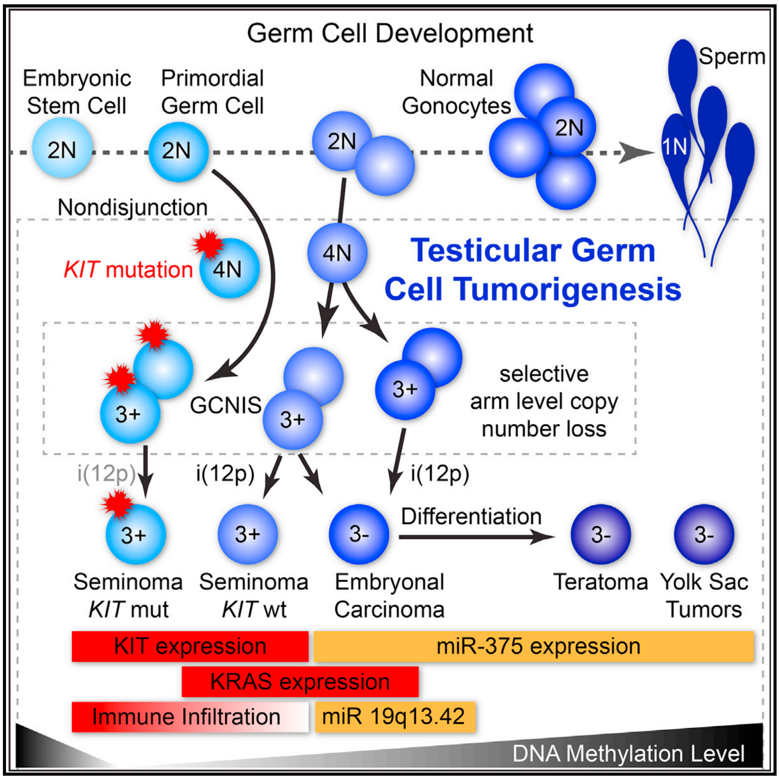

Highlights

- *KIT*-mutated seminoma has distinct DNA methylation and immune infiltration profiles
- DNA methylation and miRNA expression differ greatly between histology types
- Significant somatic mutations are present only in TGCTs with seminoma components
- All histology types exhibit extensive aneuploidy and low mutation frequency

Authors

Hui Shen, Juliann Shih, Daniel P. Hollern, ..., Katherine L. Nathanson, Victoria K. Cortessis, Katherine A. Hoadley

Correspondence

cortessis_v@med.usc.edu (V.K.C.), hoadley@med.unc.edu (K.A.H.)

In Brief

Shen et al. identify molecular characteristics that classify testicular germ cell tumor types, including a separate subset of seminomas defined by *KIT* mutations. This provides a set of candidate biomarkers for risk stratification and potential therapeutic targeting.

Shen et al., 2018, Cell Reports *23*, 3392–3406
June 12, 2018 © 2018 The Author(s).
https://doi.org/10.1016/j.celrep.2018.05.039

CellPress

OPEN ACCESS
CellPress

Cell Reports
Resource

Integrated Molecular Characterization of Testicular Germ Cell Tumors

Hui Shen,[1,32] Juliann Shih,[2,3,4,32] Daniel P. Hollern,[5,32] Linghua Wang,[6,7,32] Reanne Bowlby,[8,32] Satish K. Tickoo,[9,32] Vésteinn Thorsson,[10] Andrew J. Mungall,[8] Yulia Newton,[11] Apurva M. Hegde,[12] Joshua Armenia,[13] Francisco Sánchez-Vega,[13] John Pluta,[14] Louise C. Pyle,[14,15] Rohit Mehra,[16] Victor E. Reuter,[9] Guilherme Godoy,[17] Jeffrey Jones,[17] Carl S. Shelley,[18] Darren R. Feldman,[19] Daniel O. Vidal,[20] Davor Lessel,[21,22] Tomislav Kulis,[23] Flavio M. Cárcano,[24] Kristen M. Leraas,[25] Tara M. Lichtenberg,[25] Denise Brooks,[8] Andrew D. Cherniack,[2,3] Juok Cho,[2]

(Author list continued on next page)

[1]Van Andel Research Institute, Grand Rapids, MI 49503, USA
[2]The Eli and Edythe L. Broad Institute of Massachusetts Institute of Technology and Harvard University, Cambridge, MA 02142, USA
[3]Department of Medical Oncology, Dana-Farber Cancer Institute, Boston, MA 02215, USA
[4]Tufts University School of Medicine, 136 Harrison Avenue, Boston, MA 02111, USA
[5]Department of Genetics, Lineberger Comprehensive Cancer Center, University of North Carolina at Chapel Hill, Chapel Hill, NC 27599, USA
[6]Human Genome Sequencing Center, Baylor College of Medicine, Houston, TX 77030, USA
[7]Department of Genomic Medicine, Division of Cancer Medicine, The University of Texas MD Anderson Cancer Center, Houston, TX 77054, USA
[8]Canada's Michael Smith Genome Sciences Centre, BC Cancer Agency, Vancouver, BC V5Z 4S6, Canada
[9]Department of Pathology, Memorial Sloan Kettering Cancer Center, New York, NY 10065, USA
[10]Institute for Systems Biology, Seattle, WA 98109, USA
[11]Department of Biomolecular Engineering and Center for Biomolecular Science and Engineering, University of California, Santa Cruz, Santa Cruz, CA 95064, USA
[12]Department of Bioinformatics and Computational Biology, The University of Texas MD Anderson Cancer Center, Houston, TX 77030, USA
[13]Center for Molecular Oncology, Memorial Sloan Kettering Cancer Center, 1275 York Avenue, New York, NY 10065, USA
[14]Division of Translational Medicine and Human Genetics, Department of Medicine, Perelman School of Medicine, University of Pennsylvania, Philadelphia, PA 19105, USA
[15]Division of Genetics and Metabolism, Department of Pediatrics, Children's Hospital of Philadelphia, Philadelphia, PA 19104, USA
[16]University of Michigan Hospital and Health Systems, 2G332 UH, 1500 East Medical Center Drive, Ann Arbor, MI 48109, USA
[17]Scott Department of Urology, Baylor College of Medicine, Houston, TX 77030, USA
[18]University of Wisconsin School of Medicine and Public Health, Madison, WI 53726, USA
[19]Genitourinary Oncology Service, Department of Medicine, Memorial Sloan Kettering Cancer Center, New York, NY 10065, USA
[20]Molecular Oncology Research Center, Barretos Cancer Hospital, Rua Antenor Duarte Villela, 1331, Barretos, Sao Paolo-SP, 14784-400, Brazil
[21]Institute of Human Genetics, University of Ulm, 89081 Ulm, Germany
[22]Institute of Human Genetics, University Medical Center Hamburg-Eppendorf, 20246 Hamburg, Germany

(Affiliations continued on next page)

SUMMARY

We studied 137 primary testicular germ cell tumors (TGCTs) using high-dimensional assays of genomic, epigenomic, transcriptomic, and proteomic features. These tumors exhibited high aneuploidy and a paucity of somatic mutations. Somatic mutation of only three genes achieved significance—*KIT*, *KRAS*, and *NRAS*—exclusively in samples with seminoma components. Integrated analyses identified distinct molecular patterns that characterized the major recognized histologic subtypes of TGCT: seminoma, embryonal carcinoma, yolk sac tumor, and teratoma. Striking differences in global DNA methylation and microRNA expression between histology subtypes highlight a likely role of epigenomic processes in determining histologic fates in TGCTs. We also identified a subset of pure seminomas defined by *KIT* mutations, increased immune infiltration, globally demethylated DNA, and decreased *KRAS* copy number. We report potential biomarkers for risk stratification, such as miRNA specifically expressed in teratoma, and others with molecular diagnostic potential, such as CpH (CpA/CpC/CpT) methylation identifying embryonal carcinomas.

INTRODUCTION

The most common malignancy of young adult males of European descent are testicular germ cell tumors (TGCTs) of the type derived from germ cell neoplasia *in situ* (GCNIS) (Moch et al., 2016). There are two major histologic types: pure classic seminoma and nonseminomatous germ cell tumors (NSGCTs). The latter, comprising embryonal carcinoma (EC), choriocarcinoma,

This is an open access article under the CC BY license (http://creativecommons.org/licenses/by/4.0/).

OPEN ACCESS
CellPress

David I. Heiman,[2] Katayoon Kasaian,[8] Minwei Liu,[26] Michael S. Noble,[2] Liu Xi,[6] Hailei Zhang,[2] Wanding Zhou,[1] Jean C. ZenKlusen,[27] Carolyn M. Hutter,[28] Ina Felau,[27] Jiashan Zhang,[27] Nikolaus Schultz,[13] Gad Getz,[2,29] Matthew Meyerson,[2,3] Joshua M. Stuart,[11] The Cancer Genome Atlas Research Network, Rehan Akbani,[12] David A. Wheeler,[6] Peter W. Laird,[1] Katherine L. Nathanson,[14,30] Victoria K. Cortessis,[31,*] and Katherine A. Hoadley[5,33,*]
[23]Department of Urology, University Hospital Center Zagreb, University of Zagreb School of Medicine, 10000 Zagreb, Croatia
[24]Department of Clinical Oncology, Barretos Cancer Hospital, Rua Antenor Duarte Villela, 1331, Barretos, Sao Paolo-SP, 14784-400, Brazil
[25]The Research Institute at Nationwide Children's Hospital, Columbus, OH 43205, USA
[26]Computational Biology Center, Memorial Sloan Kettering Cancer Center, New York, NY 10065, USA
[27]National Cancer Institute, NIH, Bethesda, MD 20892, USA
[28]National Human Genome Research Institute, NIH, Bethesda, MD 20892, USA
[29]Massachusetts General Hospital Cancer Center and Department of Pathology, Massachusetts General Hospital and Harvard Medical School, Boston, MA 02114, USA
[30]Abramson Cancer Center, Perelman School of Medicine at the University of Pennsylvania, Philadelphia, PA 19104, USA
[31]Departments of Preventive Medicine and Obstetrics and Gynecology, Norris Comprehensive Cancer Center, Keck School of Medicine, University of Southern California, Los Angeles, CA 90033, USA
[32]These authors contributed equally
[33]Lead Contact
*Correspondence: cortessis_v@med.usc.edu (V.K.C.), hoadley@med.unc.edu (K.A.H.)
https://doi.org/10.1016/j.celrep.2018.05.039

yolk sac tumor, and teratoma, can contain a mix of both seminomatous and nonseminomatous components. Seminoma often has more indolent behavior, while NSGCT tends to occur at younger ages and confer higher mortality (Cortessis, 2003). TGCTs are now highly treatable, and overall relative survival of men with TGCTs exceeds 95% (Stang et al., 2013). However, survivors can experience devastating late effects of treatment, and a pressing research goal is the discovery of rational means of risk stratification that could spare some patients unnecessary chemotherapy, radiation, and surgery.

GCNIS is postulated to arise from incompletely differentiated fetal germ cells (primordial germ cells [PGCs]), based on shared morphology and immunohistochemical expression (Jørgensen et al., 1995). Both TGCTs and GCNIS cells are typically aneuploid, with hypertriploid to subtetraploid karyotypes (Summersgill et al., 2001), but GCNIS rarely exhibits 12p gains, which are pathognomonic for TGCTs (Ottesen et al., 2003). A model of tumor evolution postulates that nondisjunction creates tetraploid precursor cells, followed by a gain of isochromosome 12p during the transition from GCNIS to malignant NSGCTs (Frigyesi et al., 2004). A shared biological basis of seminoma and NSGCTs is supported by karyotypic similarities, TGCT risk alleles (Litchfield et al., 2016; Wang et al., 2017), and a report that tumor histology is unassociated in men with two primary TGCTs after adjustment for age (Thomas et al., 2013). DNA exome sequencing of several small cohorts of TGCTs have identified few significantly mutated somatic genes, primarily *KIT* and *KRAS* (Cutcutache et al., 2015; Litchfield et al., 2015; Taylor-Weiner et al., 2016). Lack of DNA methylation at CpG islands as determined by microarrays has been observed in seminomas (Smiraglia et al., 2002), and a global lack of methylated cytosines by immunohistochemistry staining has been described for GCNIS but not NSGCTs (Netto et al., 2008). Here, we characterize 137 TGCTs by DNA exome sequencing, RNA and microRNA (miRNA) sequencing, DNA SNP arrays, DNA methylation arrays, and reverse phase protein arrays.

RESULTS

Histologic Classification

Four pathologists reviewed fresh-frozen sections immediately adjacent to the tissue used for molecular analysis to confirm TGCT histology (Figure 1A). A consensus diagnosis was determined when at least three of four pathologists agreed on the tumor components and their percentage (within 10%) in the tissue block. Frozen sections of less than ideal quality were re-evaluated along with formalin-fixed, paraffin-embedded tissue sections to arrive at a final diagnosis. We used the final consensus histology from our pathology review for all of the analyses.

Samples were classified as ''pure'' for 100% and ''dominant'' for >60% presence of a given histology. The set of 137 tumors consisted of 72 seminoma, 18 EC, 9 EC dominant, 3 mature teratoma, 10 mature teratoma dominant, 3 immature teratoma dominant, 5 yolk sac tumor, 8 yolk sac tumor dominant, and 9 mixed tumors with no dominant component (Table S1). Two-class analyses compared pure seminoma (n = 72) with NSGCTs (n = 65). For patient-level analyses, we used the histology of the first or the only primary tumor (Table S2).

Sample Characteristics

We studied 137 primary TGCTs from 133 patients, including 2 tumors from 4 patients with metachronous diagnoses. NSGCTs tended to be diagnosed at younger ages than were seminomas (median 30 versus 34 years, t test p value = 0.02). A personal history of cryptorchidism was more common among men diagnosed as having seminoma (17 of 68) rather than NSGCTs (5 of 65, χ^2 p value = 0.008), but prevalence of a positive family history did not differ between these groups (χ^2 p value = 0.3). Clinical characteristics were consistent with prior reports (Table S2).

Unsupervised Classification of TGCTs

Unsupervised clustering analyses were performed to stratify tumor samples by each molecular platform (Figure S1). Seminomas were clearly distinguished from NSGCTs by DNA

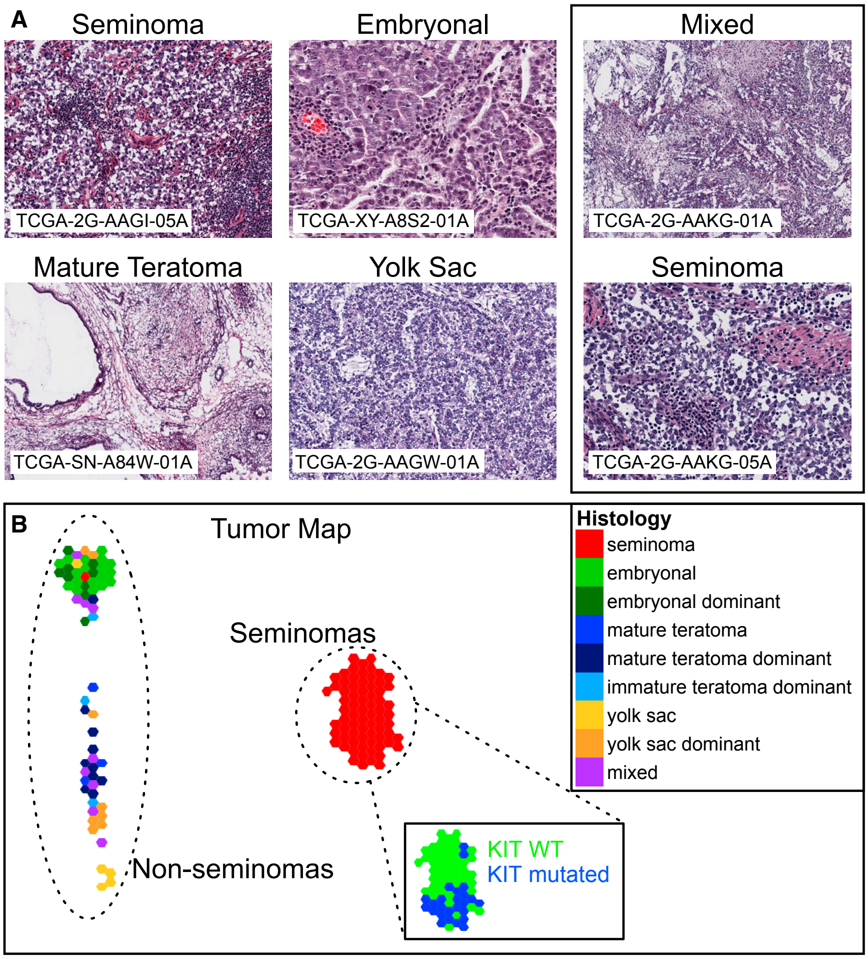

Figure 1. Histologic and Molecular Classification of TGCTs

(A) Representative images of H&E-stained slides of frozen sections are shown for seminomas, EC, mature teratomas, and yolk sac tumors. Box at right shows two asynchronous primaries from the same patient. All images 100× magnification.

(B) Tumor Map visual representation of molecular heterogeneity separating seminomas and NSGCTs. Samples are displayed as hexagons, and the spatial layout reflects sample groupings and molecular relations between samples. Samples are colored based on their histological classification. In the seminoma inset, samples are colored by KIT mutation status. *KIT* wild-type, green; *KIT* mutant, blue.

See also Figures S1 and S5 and Tables S4, S5, S6, and S7.

methylation, mRNA, miRNA, and protein. DNA copy number also distinguished seminoma from NSGCTs, although less completely.

We used Tumor Map to integrate mRNA expression, somatic copy number, and DNA methylation to visualize and spatially project relations among the samples (Newton et al., 2017). The resulting Tumor Map view (Figure 1B) completely distinguishes seminomas from NSGCTs in the molecular space, with EC-containing tumors positioned further apart from other NSGCTs. *KIT* mutation status further separates seminomas into two groups. The strong discrimination of histological types by unsupervised analysis leads us to focus subsequent analyses using the histological classification.

DNA Sequence and Content

Somatic mutation frequency varied by histology (Figure S2A). Overall median frequency, 0.5 mutations/Mb of targeted DNA (Figure 2A), was higher than that reported in pediatric tumors, but lower than most adult tumors (Lawrence et al., 2014) studied in The Cancer Genome Atlas (TCGA) (Figure S2B). The frequency of nonsynonymous mutations, 0.3 mutations/Mb, was similar to estimates from other TGCT exome-sequencing efforts (Cutcutache et al., 2015; Litchfield et al., 2015; Taylor-Weiner et al., 2016).

The most frequent type of mutation was the cytosine to thymine (C > T) transition, accounting for 40% of mutations (Table S1). Using mutational signature analysis as described by Covington et al. (2016), levels of C > T transition at CpG dinucleotides was significantly lower in seminoma with somatic *KIT* mutations than in either seminoma with wild-type *KIT* or NSGCTs ($p = 0.002$; Figure S2C). This signature, which correlates with Catalogue of Somatic Mutations in Cancer (COSMIC) mutation signature 1, is observed in most human tumors and postulated to result from the accumulation of 5-methylcytosine deamination events (Alexandrov et al., 2013).

Three genes were significantly somatically mutated: *KIT* (18%), *KRAS* (14%), and *NRAS* (4%) (Figure 2C), all described previously in TGCTs (Litchfield et al., 2015; Tian et al., 1999). These genes were exclusive to seminomas except for one *KRAS* mutation in an NSGCT with 30% seminoma. The *KIT* mutations were located in the activation loop of the KIT protein tyrosine kinase 2 (n = 19), the juxtamembrane domain (n = 6), and the protein tyrosine kinase 1 domain (n = 1), resembling those previously described in TGCTs (Litchfield et al., 2015) and intracranial germ cell tumors (Wang et al., 2014) (Figure S2D). *RAS* mutations clustered at known mutation hotspots (Figure S2D) and mutations in *KRAS* and *NRAS* co-existed in only one seminoma (Figure 2C). These mutations were particularly prevalent in seminomas diagnosed in men with a history of cryptorchidism (13 of 17). Of the six seminomas with mutations in both *KIT* and *KRAS*/*NRAS*, four were in men with a history of cryptorchidism (odds ratio = 7.3 [95% confidence interval 1.2–45.0]), all in the ipsilateral testicle. The PI3-kinase pathway influences germ cell proliferation in a Kit/Kit ligand-dependent fashion (Cardoso et al., 2014). Of note, three seminomas contained *PIK3CA* mutations, two at E545K and one at N345K (Figure S2D). Somatic *PIK3CA*

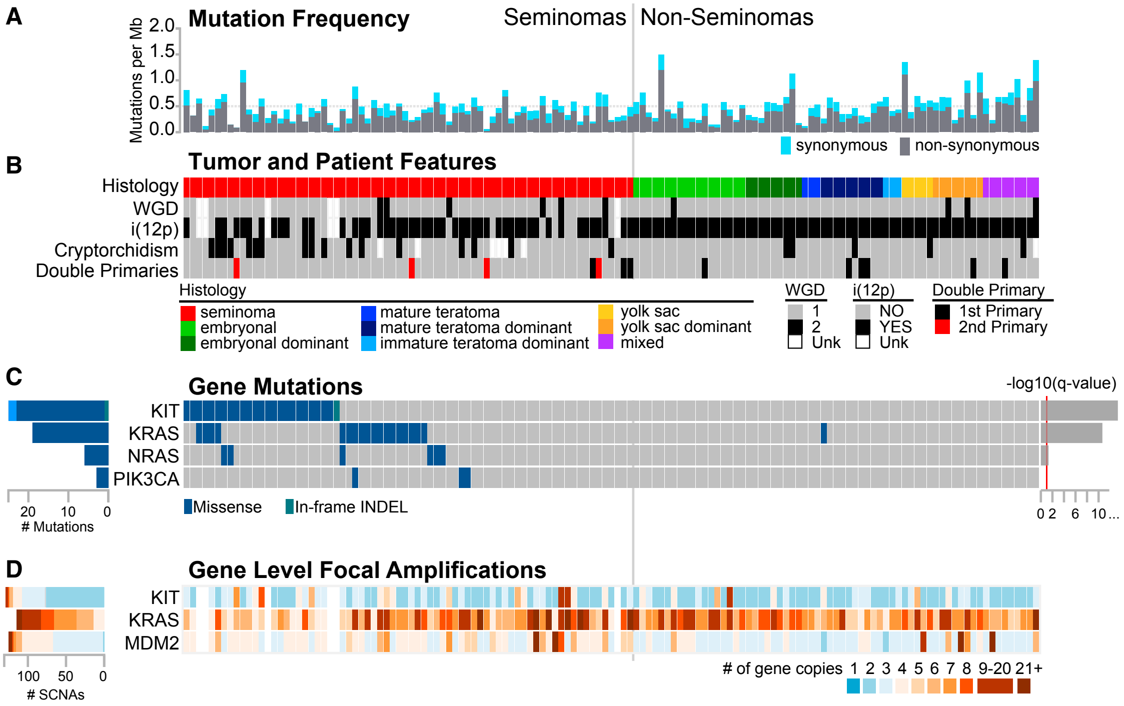

Figure 2. Molecular Alterations and Features across 137 TGCT Samples

(A) Somatic mutation frequency (mutations/Mb) from exome sequencing. The horizontal gray dashed line marks the median mutation rate of 0.5 mutations/Mb. The vertical gray line divides pure seminomas from NSGCTs.

(B) Tumor and patient features per sample. Whole genome doubling (WGD) and i(12p) status are using the ABSOLUTE algorithm. Calls for WGD or inferred i(12p) status could not be made for six low-purity samples. Cryptorchidism status, family history of testicular germ cell tumor (TGCT) or other cancer, and presence of double primaries are displayed. Unk, unknown.

(C) Significant recurrent mutations (*KIT*, *KRAS*, and *NRAS*) or curated based on frequency or biological relevance.

(D) Three known oncogenes were significantly focally amplified. Values represent the number of gene copies detected using the ABSOLUTE integer copy number.

See also Figures S2 and S3 and Table S3.

mutations have been reported previously in two platinum-resistant TGCTs (Feldman et al., 2014). Only five other recurrently mutated genes were observed in our cohort, most with likely non-pathogenic mutations.

SCNAs

All TGCTs had ploidy exceeding two, but NSGCTs demonstrated significantly lower ploidy than seminomas (median 2.8 versus 3.1, $p = 1.3 \times 10^{-8}$, Mann-Whitney *U* test) with variability across histology types (Figure S3A). Increased chromosomal content above a ploidy of two suggests that whole-genome duplication (WGD) occurred in all of the samples, and ten samples had evidence of two WGD events (Figure 2B), which is consistent with the proposed model of WGD followed by the deletion of chromosome arms (Frigyesi et al., 2004). Chromosome arm loss after WGD was specific to histological subtypes. NSGCTs had fewer copies of chromosomes (Chr) 19q, 15, 22, 19p, 10q, 8p, 2q, and 8q, whereas seminomas had fewer copies of 11q (Figure S1E). Even for arm-level somatic copy number alterations (SCNAs) shared between histologies, the timing of alterations differed between seminomas and NSGCTs, as inferred from the frequency of each event and the level of aneuploidy (Table S3; Figure S3C). For example, the deletion of Chr 4 was inferred to be an early event and 1p to be moderately early in all of the samples, whereas the deletion of 11q was inferred to be early only in seminomas and the deletion of Chr 15 to be early only in NSGCTs. We could not assess the copy number for six samples because of the low tumor purity.

We observed allelic copy number profiles consistent with the presence of at least one isochromosome 12p (i[12p]) in 114 of 131 (87%) tumors. All 17 tumors inferred lacking the i(12p) event were seminomas (Figure 2B) and retained at least 4 copies of 12p (Figure S1E). Only 2 of 131 samples exhibited loss of 12q heterozygosity, suggesting that most tumors had undergone a second WGD or a Chr 12 duplication event before i(12p) formation, as previously described (Geurts van Kessel et al., 1989).

We observed significantly recurrent focal amplifications of *KIT*, *KRAS*, and *MDM2* (Figures 2D and S3D)(McIntyre et al., 2004, 2005; Mostert et al., 2000). These amplifications contained entire genes and occurred with similar frequency in seminomas and NSGCTs. Seminomas with increased copies of *KRAS* (Chr 12) were more likely to have wild-type *KIT* (Figure 2D; t test $p = 0.0007$). Significantly reccurring focal deletions in chromosomal fragile sites *GRID2/ATOH1*, *JARID2*, *WWOX*, *NEGR1*, *PDE4D*, and *PARK2* occurred almost exclusively in NSGCTs and were shorter than the genes that they affected (Figure S3D).

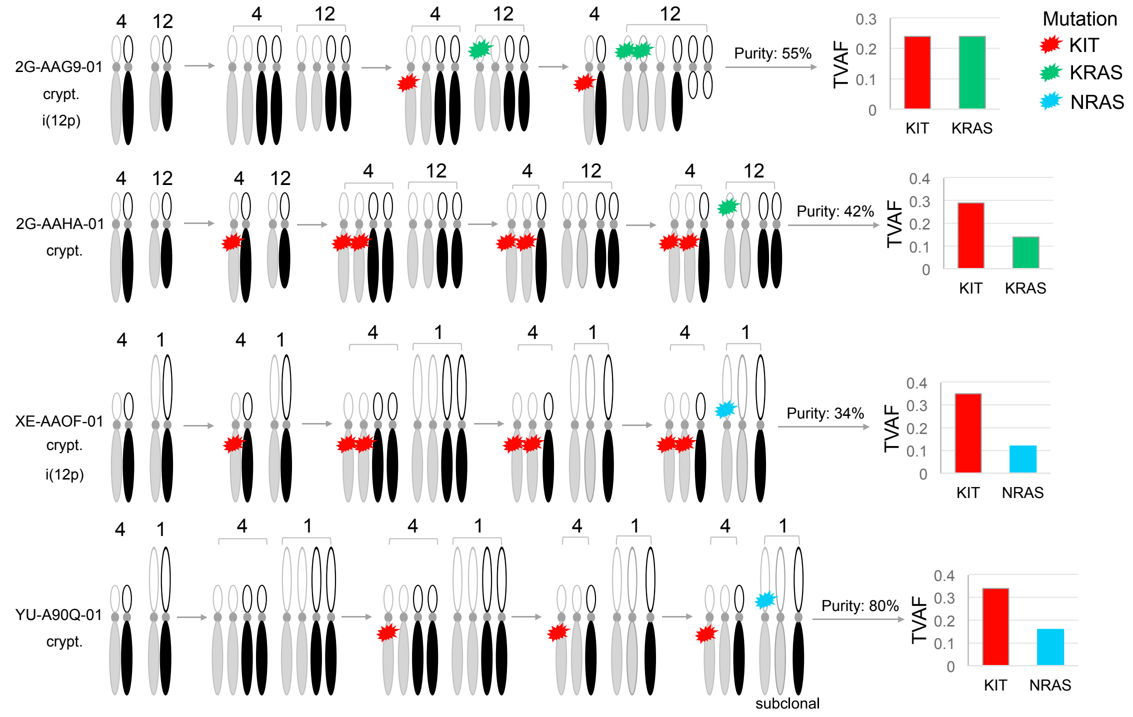

Figure 3. Inferred Order of Somatic Mutations and DNA Copy Number Alterations in TGCTs

Four seminomas with co-existing somatic mutations in *KIT*, *KRAS*, and *NRAS* were selected. The timing of somatic events within each sample was inferred by integrated analysis of mutation multiplicity, allelic integer copy number, and whole-genome doubling status. Mutation multiplicity (s_q) was calculated from purity, total copy number (CN), and tumor variant allele fraction (TVAF) as follows: s_q = TVAF[(CN*purity)+(2*(1−purity))]/purity. Integer copy number, whole-genome doubling status, and purity of tumor genomes were calculated using the ABSOLUTE algorithm. Cryptorchidism (crypt), isochromosome 12p [i(12p)]. Gray and black identify homologous chromosomes.

Inferred Order of Major Genetic Alterations

We inferred the relative order of alterations in tumors with mutations and sufficient tumor purity for estimating copy number. We used the variant allele fraction, allelic integer copy number, WGD, and purity estimates to calculate the mutation multiplicity, an inferred measurement of the number of alleles with a mutation. Four examples with mutations in both *KIT* and *KRAS/NRAS* are illustrated in Figure 3. Somatic *KIT* mutations were inferred to occur before WGD in two samples. *KIT* mutant multiplicities of an additional eight seminomas present a similar pattern, with variant allele fractions from DNA and RNA indicating a clonal nature. Genetic activation of *KIT* may arise early in TGCT tumorigenesis. In contrast, *RAS* mutations were inferred to be later events, all occurring after WGD. With the *KRAS* locus on 12p, we were able to infer the relative order between *KRAS* mutations and inferred i(12p) formation for 10 samples. Six samples had low mutation multiplicities, suggesting that they arose after i(12p) on either allele, while four samples had increased mutation multiplicities, suggesting that mutations arose before or during i(12p) formation. The other samples lacked i(12p) (n = 3), had low purity (n = 2), or we were unable to infer the order of events (n = 4). The number of wild-type (WT) *KRAS* copies was correlated with expression, but the number of mutant *KRAS* copies was not (Figure S3E).

DNA Methylation

Histological subtypes exhibited dramatically different global DNA methylation patterns. DNA methylation level as methylation fraction at a single locus is measured by the beta value, ranging from 0 to 1. For NSGCTs, the overall distribution of beta values at canonical CpG sites (Figure 4A) followed the bimodal pattern that is characteristic of most primary human tissue samples, with peaks for unmethylated and methylated CpGs. However, the methylated peak was not observed in seminomas, which instead demonstrated intermediate DNA methylation peaks in addition to the unmethylated peak, suggesting that seminoma samples contained two major cell types, one completely unmethylated and the other with full methylation at a subset of the loci. Using cell-type DNA methylation signatures, we identified infiltrating lymphocytes as the contaminant (Figure S4A), which is consistent with prior reports of extensive lymphocytic infiltration in seminomas (Hvarness et al., 2013; Parker et al., 2002). We estimated the percentage of lymphocytes in each tumor using cell-specific DNA methylation patterns. We also estimated tumor purity with ABSOLUTE (Carter et al., 2012), using copy number and mutation data (Figure S3B). A near-perfect anti-correlation ($R = -0.93$, $p < 0.0001$; Figure 4B) was observed between estimated lymphocyte fraction and tumor purity, validating both methods. Subtraction of lymphocyte DNA methylation

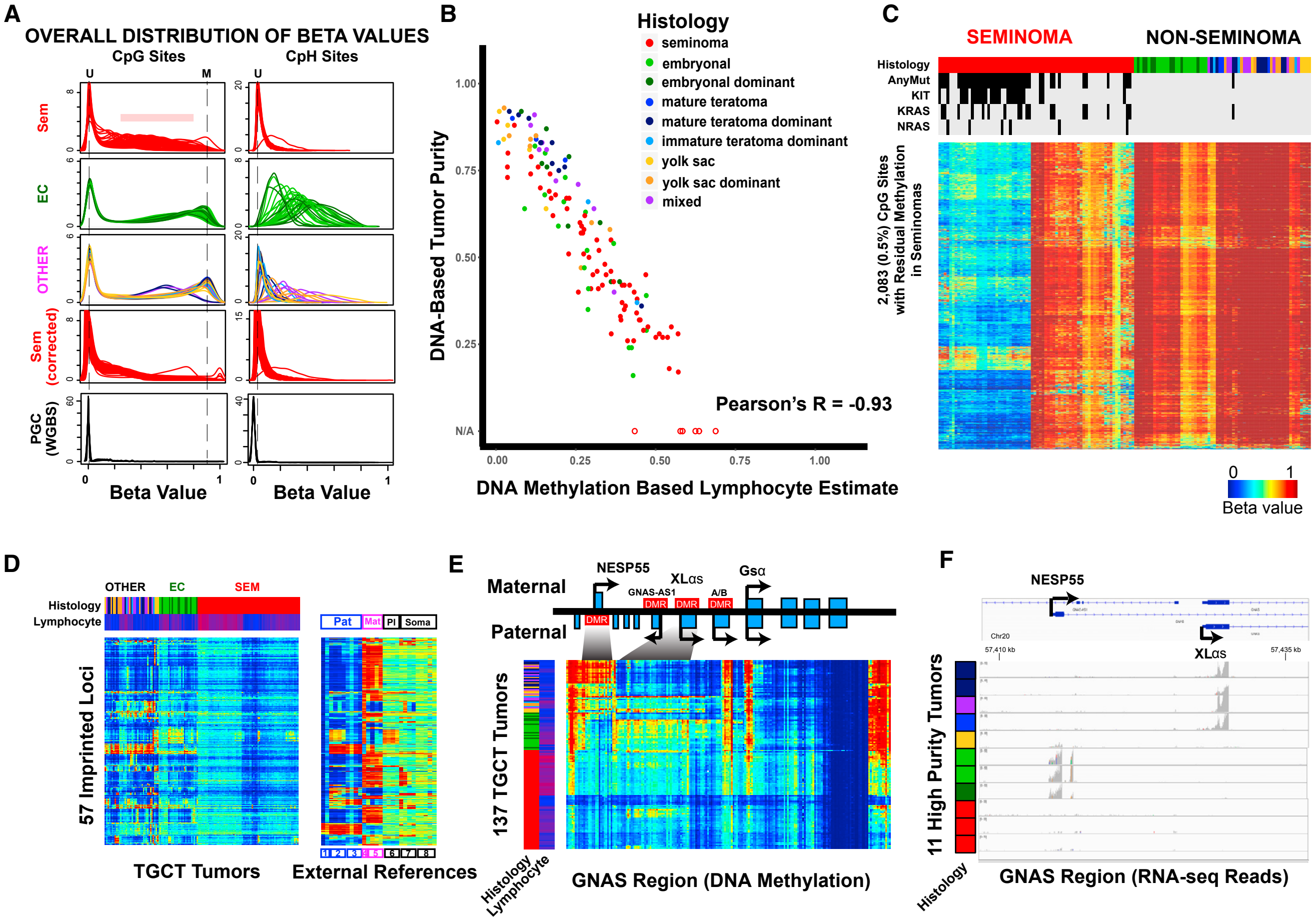

Figure 4. Dramatic DNA Methylation Differences Observed between TGCT Histology Types

(A) Smoothed density plots show overall distributions of beta values at CpG (left) and CpH sites (right) grouped by seminoma, EC and EC dominant, and other (OTHER) tumors. Vertical dashed lines indicate locations corresponding to unmethylated (U) and methylated (M) sites. Four primordial germ cell (PGC) samples from external whole-genome bisulfite sequencing (WGBS) studies are plotted for the same sites included on the HM450 arrays.

(B) Overall correlation between the DNA methylation signature-based lymphocyte estimates (x axis) versus mutation and SNP array-based (ABSOLUTE) tumor purity estimates (y axis) for 131 tumors. Six additional tumors without ABSOLUTE estimates as a result of extremely low purity are plotted with hollow circles.

(C) DNA methylation at 2,083 (0.5%) loci (rows) with residual methylation in seminomas (columns) differs based on *KIT*/*RAS* mutation status. Data are corrected for lymphocyte infiltration (uncorrected data shown in Figure S1D). Blue to red indicates 0% to 100% methylation. Top color bars annotate the histology of each tumor and mutation status in *KIT*/*KRAS*/*NRAS* (black, mutants; gray, wild-type).

(D) DNA methylation patterns at 57 imprinted loci. Inferred lymphocyte fraction is included as the second column color bar (blue to red: low to high level of contamination). External reference data (right) are plotted for the same set of loci representing paternal (Pat; 1, sperm; 2, hydatidiform mole; 3, paternal Unipaternal Disomy [pUPD] leukocyte), maternal (Mat; 4, maternal Unipaternal Disomy [mUPD] leukocyte; 5, parthenogenetically derived oocytes), and placental (Pl; 6, placenta)-imprinting patterns, in addition to ESCs (7, ESCs from 2, oocyte) and somatic tissues (8, somatic tissues).

(E) The *GNAS* complex locus demonstrates contrasting DNA methylation patterns in different subtypes. Seminomas show an overall lack of methylation (observed methylation explainable by lymphocytic infiltration); EC and EC-dominant tumors show extensive methylation at the paternal DMR at the NESP55 promoter, and other tumors tend to have methylation at the maternal DMR near the XLαs promoter.

(F) RNA-seq reads for different *GNAS* transcripts are consistent with DNA methylation patterns. Eleven tumors with relatively high purity of different histologies are shown.

See also Figure S4.

contribution from all tumors led to the disappearance of intermediate methylation peaks in seminomas (Figure 4A), whereas the methylated peak remained in NSGCTs (Figure S4B). Consistent with a PGC origin for seminoma, the corrected density plot shows the majority of CpGs to be completely unmethylated in seminomas, similar to public PGC data (Figure 4A).

EC exhibited extensive methylation at non-canonical cytosine sites (e.g., CpA, CpT, CpC), collectively termed CpH sites (Figure 4A). CpH methylation was observed in tumors with an EC component, which is highly correlated with the pathological quantification of EC content (R = 0.86; Figure S4E) and associated with a high mRNA level of *de novo* DNA methyltransferases, DNMT3A/3B (Figure S4G). CpH methylation was first described

in embryonic stem cells (ESCs) (Lister et al., 2009). Analysis of external PGC data (Figure 4A) revealed a lack of CpH methylation, indicating that this epigenetic similarity between EC and ESCs is not shared with PGCs (Figure S4D).

Global methylation is low in seminomas, with recurrent methylation observed at only <1% of all of the sites included on the HM450 array (Figure 4C). After correction for lymphocytes, the remnant methylation is absent in a subgroup of seminomas that are highly enriched for *KIT*/*KRAS* mutations (Figures 4C and S4C; $p < 0.0001$), suggesting an essentially complete lack of DNA methylation in this subset.

We comprehensively surveyed imprinted loci identified on the HM450 platform (Court et al., 2014). In seminomas, the degree of observed DNA methylation at these imprinted loci was in general lower than the levels that are characteristic of the biparental imprinting of soma (~0.5) and consistent with lymphocytic infiltration (Figure 4D). This pattern is expected in seminomas free of methylation at imprinted loci but contaminated by lymphocytes. The methylated CpG signal at these imprinted sites disappeared after correction for lymphocyte methylation in seminomas (data not shown), confirming the general lack of methylation at these sites. DNA methylation at imprinted loci was largely erased in NSGCTs as well, although some NSGCTs exhibited methylation at certain imprinted loci (Figure 4D), notably the *GNAS* complex imprinted locus, for which paternally, maternally, and biallelically expressed transcripts have been reported. Non-EC NSGCTs tended to be methylated at the paternal differentially methylated region (DMR), while EC had intermediate to high methylation at the maternal DMR (Figure 4E). RNA sequencing (RNA-seq) data from this region confirmed alternative usage of the promoters (Figure 4F) consistent with the observed DNA methylation pattern.

We investigated whether the presence of global DNA methylation in NSGCTs was random or followed certain patterns by examining the distribution of methylation by chromatin states in H1 ESCs (Figure S4F). We observed that active promoters in H1 ESCs, usually CpG islands, are generally unmethylated, whereas heterochromatin regions are extensively methylated. Thus, if this methylation was re-established after the DNA methylation nadir of PGCs, it largely followed preset rules similar to those in normal development, despite a failure to correctly establish imprinting methylation. EC exhibited overall DNA methylation similar to H1 ESCs (Figure S4D). However, poised (bivalent) promoters, which are prone to cancer-specific gain of methylation (Widschwendter et al., 2007), exhibited gain of methylation in NSGCTs. These sites include tumor suppressors, for which epigenetic silencing could contribute to tumorigenesis.

We observed epigenetic silencing of important tumor suppressors, including *BRCA1* (Koul et al., 2002), *MGMT* (Martinelli et al., 2016), and *RASSF1A* (Honorio et al., 2003) exclusively in NSGCTs (Figure S4H). We found epigenetic silencing of *RAD51C* in 16 NSGCTs (Figure S4I). *BRCA1* and *RAD51C* both are involved in the homologous recombination (HR) DNA repair pathway. Epigenetic silencing of *RAD51C* has been described in ovarian cancer with *BRCA1* deficiency (The Cancer Genome Atlas Research Network, 2011) but not in TGCTs. A locus containing *RAD51C* has been associated with TGCT susceptibility (Chung et al., 2013), highlighting the potential importance of homologous repair deficiency in TGCTs. We also found epigenetic silencing in *DNAJC15*/*MCJ* (Figure S4H), which in breast and uterine cancer cells has been associated with drug resistance (Fernández-Cabezudo et al., 2016).

Expression of miRNA, mRNA, and Protein in TGCTs

Profiles of miRNA, mRNA, and protein differed between seminomas and NSGCTs. We noted several associations and confirmed previously reported characteristics, such as high *KIT* gene and protein expression in seminoma (Tables S4, S6, and S7).

EC tumors were distinguished by the high expression of numerous miRNAs. Expression of the *miR-519* genomic cluster on 19q13.42 was 25- to 50-fold higher in EC than in seminoma and 300- to 600-fold higher than in other types of NSGCTs, but it was not associated with copy number gain (Figure S5A). The miRNAs in this cluster have been shown to also be expressed in ESCs (Wilson et al., 2009). These miRNAs are likely to negatively regulate the expression of mRNA in EC because many of their targets have lower expression in EC (Table S6).

The miRNAs *miR-371*, *miR-372*, and *miR-373* have been proposed as serum biomarkers for monitoring patients with TGCTs for active disease as a strategy to minimize systemic therapy and attendant late effects (Syring et al., 2015). Highest sensitivity and specificity were reported for *miR-371a-3p* (Dieckmann et al., 2017). We interrogated 30 other TCGA tumor types and found *miR-371a-3p* to be dramatically overexpressed in TGCTs (Figure S5B), specifically seminoma, EC, and mixed NSGCTs, but minimally expressed in teratomas (Figure S5C). Conversely, *miR-375* was highly expressed in teratomas, yolk sac tumors, and mixed tumors containing these elements, but not in seminoma or EC (Figure S5C). Using a random forest classification, we defined a ranked series of miRNAs that distinguish seminoma, EC, and other NSGCTs (Table S5).

Using Paradigm to infer the activity of proteins, complexes, and general processes based on copy number and gene expression data, we identified seven major pathway activity clusters (Figure S1G). Three clusters, including *KRAS* signaling and immune infiltration, showed enriched activity in seminomas. All NSGCTs had enriched pathway activity for Wnt and MYC signaling. Samples with teratoma components had high pathway activities for the mammalian target of rapamycin (mTOR) and myogenesis, which is consistent with their differentiated nature.

Immune Infiltration in Seminomas

Extensive immune infiltration was noted in many of our seminoma samples during pathologic review and in the DNA methylation analysis (Figure S4A). Expression of 78 published immune gene expression signatures correlated with our DNA methylation-based lymphocyte content estimates (Figure 5A) and were highest in seminoma with *KIT* mutations compared to other samples (Figures 5B and 5C). The gene signatures suggest infiltration of several specific types of T cells (cytotoxic, $CD8^{+}$, T central memory, T effector memory, and regulatory T cells), B cells, and activated dendritic cells.

We further analyzed T cell receptor (TCR; Figure S6A) and B cell receptor (BCR; Figure S6B) diversity across the sample set. Seminoma samples had both higher levels of TCRs and higher diversity of BCRs and TCRs. Examining total mutation

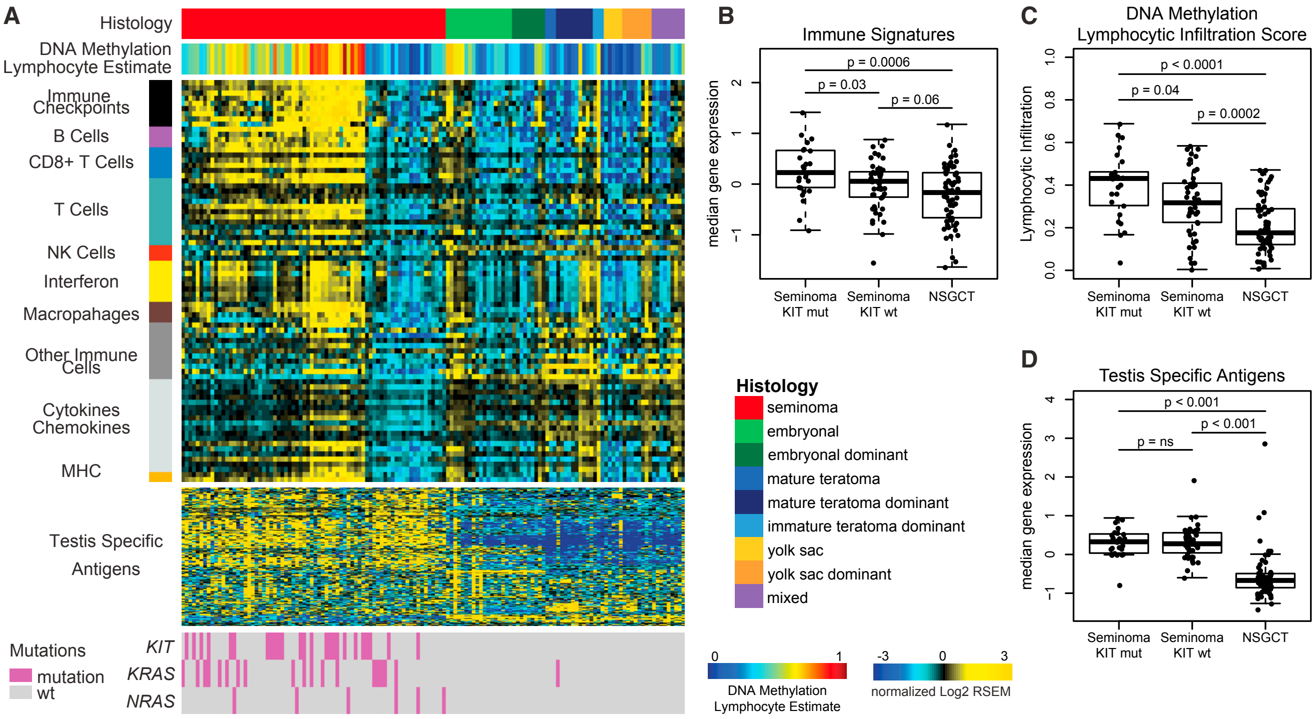

Figure 5. Immune Signatures Are High in Seminomas

(A) Gene expression data (log2 median-centered RNA-seq by expectation maximization [RSEM] values) are displayed for 78 published gene expression signatures and ordered by immune category (left vertical bar). Tumors are ordered by histology and clustered by gene expression. Annotation tracks for DNA methylation lymphocyte infiltration score and mutation status are displayed.

(B–D) Boxplots of immune features comparing seminoma *KIT* mutant, seminoma *KIT* WT, and NSGCTs. (B) Median expression of immune signatures, (C) DNA methylation lymphocytic infiltration scores, and (D) median cancer-testis-specific antigen gene expression. Boxplots display the median value, upper and lower quartiles, and the whiskers represent the interquartile range. Each dot represents the value of a single sample.

See also Figure S6.

load and predicted neoantigens, we identified high-affinity peptides but no specific antigen. Neither neoantigen signal nor total mutation load correlated with immune signatures (data not shown). We also did not find any association between immune cell signatures and either total or individual viral loads by mapping RNA-seq data to viral genomes (data not shown). Seminomas had higher expression of T effector memory cell signatures, suggesting the presence of antigen-experienced T cells. We interrogated mRNA levels of established cancer-testis-specific antigen (CTA) genes (Almeida et al., 2009) and noted higher levels in seminomas (Figure 5A), which may explain a polyclonal antigen-driven immune response around the tumor. However, although immune infiltration was increased in seminomas with *KIT* mutations, CTA genes expression levels did not differ by KIT status within seminomas (Figure 5D).

KIT Pathway Alterations

Spermatogenesis requires coordinated germ cell proliferation and apoptosis, partly governed by KITLG-mediated KIT signaling via the PI3K pathway in mammals. All of the tumors had at least one risk allele as defined by each of two *KITLG* polymorphisms, which is consistent with prior germline data (Kanetsky et al., 2009). We calculated the percentage of alterations from mutations, copy number, and gene expression, and the KIT-PI3K pathway was the only enriched pathway, predominantly in seminomas (Figure 6A). This pathway includes five recurrently mutated genes: *KIT*, *KRAS*, *NRAS*, *PIK3CA*, and *PIK3CD*. Not only were *KIT* mutations enriched in seminomas but also *KIT* mRNA and protein were highly expressed in seminomas (Figure 6B). Within seminomas, *KIT* gene expression was higher in *KIT*-mutated tumors than in *KIT*-WT tumors, confirming the gain-of-function nature of these mutations ($p = 0.001$; Figure S7A). *KIT* focal amplifications were rare and did not, in general, amplify mutated copies of *KIT* (Table S1). Compared to NSGCTs, even seminomas without a *KIT* mutation or *KIT* focal amplification had higher expression of *KIT* mRNA and protein. *KIT* mutant seminomas had lower-level copy number levels and gene expression of *KRAS* than either *KIT* WT seminomas or NSGCTs. Gene signatures downstream of KIT signaling such as AKT, PI3K, KRAS, and JAK/STAT were high in seminomas, regardless of *KIT* or *KRAS* mutation status.

CBL, which regulates ubiquitin-mediated degradation of KIT, was deleted in 48% of KIT-WT seminomas, leaving just one copy (Figure 6B). *CBL* copy number negatively correlated with KIT protein expression for most tumors; however, seminomas with *KIT* or *KRAS* mutations maintained high protein levels of KIT regardless of *CBL* copies, apparently escaping *CBL* regulation (Figure S7C). In NSGCTs, we also observed high expression

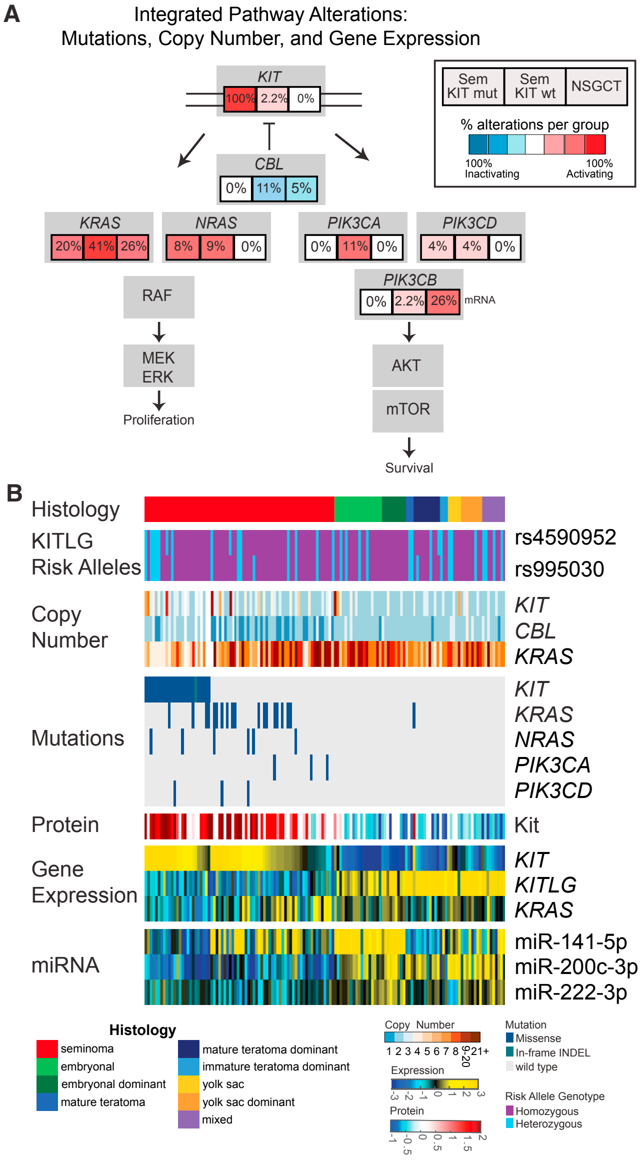

Figure 6. Analysis of *KIT* and *KIT* Ligand in TGCTs

(A) Integrated analysis of the Kit pathway indicating the frequency of multiple genomic alterations within the Kit pathway.

(B) A multiple platform characterization of *KIT* and *KIT* ligand across testicular germ cell tumors. Samples are first ordered by tumor histology. Within histology, tumors are ordered by *KIT* mutation status and then by *KIT* mRNA expression from high to low. Missing values are depicted as blank or white space within each heatmap.

See also Figure S7.

of *miR-222-3p*, a validated miRNA regulator of KIT. In tumors expressing the miR, *KIT* gene expression levels were low (Spearman's rho = −0.55; Figure S7B; Gits et al., 2013).

Double Primary TGCTs

Approximately 2%–4% of men diagnosed as having TGCTs develop a second primary TGCT in the contralateral testicle (Fosså et al., 2005). We molecularly profiled both tumors from four men. One first primary was seminomas and three were NSGCTs; all second primaries were seminomas. The three histologically discordant pairs exhibited notably different profiles of all molecular features. Among all of the data types, the miRNA data were most highly correlated within all of the pairs (Figure 7). No mutation was shared between paired primaries (Figure 7A), as reported in three other pairs (Brabrand et al., 2015). Data from the two seminoma primaries of TCGA-2G-AAHP were the most similar across all of the platforms, even though this patient had received radiation between primaries. The apparent difference in ploidy and DNA methylation was the result of different amounts of lymphocytic contamination. Somatic mutation profiles yet again diverged between the two tumors, suggesting that genetic mutations are likely later events in these patients, and early copy number, epigenetic alterations, or both produce cells that are prone to transformation. The primaries from TCGA-2G-AAKG were divergent in histology, with the first tumor being a mixed tumor with 40% EC and the second being a pure seminoma. However, their DNA methylation profiles, both at CpG and CpH sites, were highly similar. The presence of CpH methylation in the first tumor is explained by the EC component, but CpH methylation in the second was unexpected, considering this sample had molecular and histologic appearances consistent with seminoma. This patient was the only one of the four with documented chemotherapy delivered between the two primary tumors.

DISCUSSION

Integration of tumor characteristics and genomic and epigenomic data revealed distinctive molecular landscapes of TGCT histologic types and identified previously unappreciated diversity within seminomas (Table 1). All of the samples evinced WGD and a low mutation density. Only a few driver mutations were identified, exclusively in seminomas or samples with seminoma components. *KIT*-mutated seminomas separated from the KIT-WT seminomas on the Tumor Map and exhibited unique characteristics, including the highest levels of lymphocyte infiltration, the absence of global DNA methylation, reduced *KRAS* mutation frequency and copy number alterations, reduced frequency of estimated presence of inferred i(12p) events, and a more prevalent history of cryptorchidism (Table 1). Because *KIT* mutation was never observed in tumors lacking seminoma components, we postulate that this subset of seminomas is locked in a PGC-like status and remain pure seminomas, while those lacking *KIT* mutations may have the potential to differentiate into other histologies. We showed that cryptorchidism was enriched in seminomas, especially in men with *KIT*-mutated seminomas, shedding new light on established cryptorchidism-TGCT associations that warrant further investigation (Banks et al., 2013). All of the subtypes of NSGCTs shared genomic characteristics, including lower ploidy and higher purity than seminoma, and universal i(12)p. Recurrent somatic mutations were rarely present in NSGCTs, even though the overall mutation density was not dramatically different from seminomas (Table 1).

Previous studies noted both extensive lymphocytic infiltration and lack of DNA methylation in seminomas, features that we show for the first time to be more extreme in *KIT*-mutated

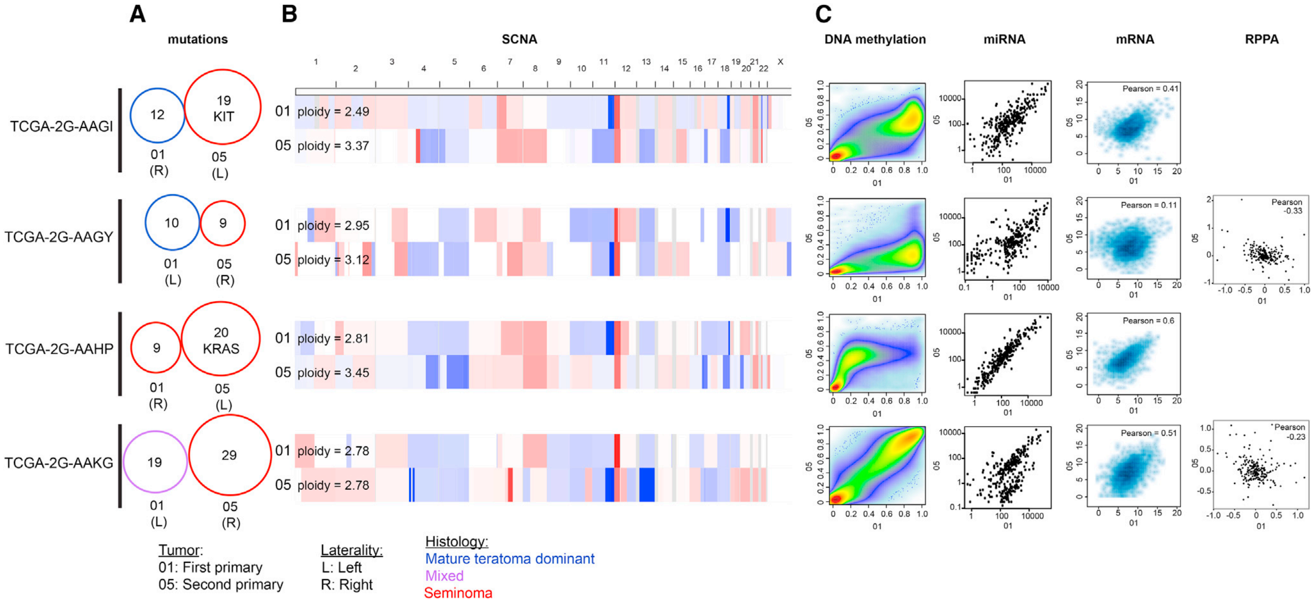

Figure 7. Genomic Alterations and Features in Four Patients with Asynchronous Double Primaries

(A) Venn diagrams showing no overlap between somatic mutations identified in the first and second primary tumors, with number of significantly mutated genes shown. 01, first primary; 05, second primary; R, right; L, left.

(B) SCNAs across the genome relative to the tumor ploidy (also shown). Red, amplification; blue, deletion.

(C) For each platform, the first primary tumor is on the x axis and the second primary tumor is on the y axis. From left to right: beta values as a measure of DNA methylation across probes, with color representing the smoothed density of the probes; the reads per million (RPM) abundance of 303 miRNAs used in unsupervised clustering analysis on a log scale; log2 mRNA expression (RSEM) of 2,878 variably and highly expressed genes used for unsupervised clustering; RPPA expression values for 218 antibodies assayed. RPPA was not assessed for at least one primary tumor for patients TCGA-2G-AAGI and TCGA-2G-AAHP.

seminomas. Signals from infiltrating cells influence genomic readout from the mixture of cellular components in the bulk tissue analyzed and need to be distinguished from tumor-specific signals. In our study, almost all of the DNA methylation signal in seminomas came from lymphocytes. Only by removing it were we able to reveal that seminomas lacked methylation genome wide and that those with *KIT* mutations had more complete lack of methylation. Although the recruitment and role of lymphocytes in TGCTs remain unclear, this immune response is likely multiclonal in nature because we did not observe clonal restriction of BCRs or TCRs. Demethylating agents were shown to elicit an immune reaction via "viral mimicry" caused by demethylation and consequent expression of endogenous retroviral elements (Chiappinelli et al., 2015; Roulois et al., 2015). Globally demethylated genomes of *KIT*-mutated seminoma cells could provoke a similar immune response. In line with this hypothesis, overexpression of human endogenous retroviral loci was reported in several seminoma samples (Gimenez et al., 2010). Global demethylation in *KIT*-mutated seminomas also may explain their significantly lower COSMIC mutational signature 1, because 5-methylcytosine, which occurs primarily in the CpG context, is 10 times more likely to mutate than a regular cytosine (C → T in the CpG context, explaining the majority of mutations that are observed in human cancers) (Shen and Laird, 2013).

TGCT models acknowledge that seminomas most closely resemble PGCs and GCNIS based on histologic appearance, gene expression, and lower levels of DNA methylation. GCNIS or seminomas are proposed precursors of EC, which is in turn the proposed precursor for extraembryonic (yolk sac tumor and choriocarcinoma) and somatic (teratoma) lineages (Honecker et al., 2006). We postulate that only seminomas without *KIT* mutations may be capable of acquiring nonseminomatous histology because all NSGCTs, including mixed TGCTs with seminoma components, lacked *KIT* mutations. Activating *KIT* mutations may lock KIT mutant seminoma cells into a PGC-like state in which *UHRF1* and *DNMT1* expression are suppressed, preventing the development of NSGCT components, which appear to require DNA methylation capacity. This may explain why seminomas have proven difficult to propagate *in vitro*, because DNA methylation at certain sites is essential for the survival of cancer cell lines (De Carvalho et al., 2012). Analysis of an external dataset (GSE60787) shows that TCAM-2, the sole seminoma cell line derived to date, has substantial DNA methylation.

Likely starting from an unmethylated precursor (PGC/GCNIS), NGSCTs re-establish methylation patterns corresponding to their cellular phenotypes: the epigenetic profile of EC resembles that of ESC, with extensive non-canonical CpH methylation (Lister et al., 2009), while non-EC NSGSTs adopt DNA methylation patterns resembling soma and extraembryonal lineages. Frequent promoter DNA methylation inactivating genes in the HR pathway also occurs in non-EC NSGSTs, so acquisition of DNA methylation capacity could be a key step for NSGCT precursors to embark on the path toward EC and its differentiated lineages. However, because imprinting methylation is never properly re-established, early genetic, epigenetic, or genetic

OPEN ACCESS CellPress

Table 1. Summary of Clinical, Epigenetic, and Molecular Alterations in TGCTs

		Seminoma		NSGCT-EC	NSGCT-Other		
		Seminoma *KIT* Mutant	Seminoma *KIT* Wild-Type	EC	Teratoma	Yolk Sac	Mixed NSGCTs
	Samples, no.	25	47	27	16	13	9
	Cryptorchidism, no. (%)	10 (40)	7 (14.9)[a]	2 (7.4)	1 (6.3)	1 (7.7)	1 (12.5)[a]
	Age at diagnosis, y, median (range)	34 (20–52)	31 (20–47)	26 (14–67)	28 (20–53)	31 (20–66)	28 (19–39)
DNA Methylation	Overall DNA methylation	fully unmethylated	residual methylation	ESC-like CpG + CpH	CpG methylation, CGI hypermethylation		
	Imprinting	fully erased	ully erased	fully erased; methylated *XLαs*	fully erased; methylated *NESP55*		
	Promoter Methylation						
	BRCA1, no. (%)	0	0	2 (7.4)	5 (31.2)	7 (53.8)	2 (22.2)
	RAD51C, no. (%)	0	0	3 (11.1)	5 (31.2)	4 (30.8)	4 (44.4)
	MGMT, no. (%)	0	0	3 (11.1)	9 (56.2)	5 (38.5)	4 (44.4)
	DNAJC15, no. (%)	0	0	0	8 (50)	4 (30.8)	1 (11.1)
	Leukocyte infiltration, median (range)	0.43 (0.04–0.69)	0.32 (0.004–0.58)	0.28 (0.08–0.47)	0.16 (0.01–0.46)	0.11 (0.01–0.36)	0.16 (0.11–0.37)
mRNA	Lymphocyte expression signatures	High	medium–high	low	low	low	low
	CTA expression	High	high	low	low	low	low
Mutations	Mutation rate, total mutations/Mb, median (range)	0.44 (0.12–1.28)	0.44 (0.26–1.07)	0.46 (0.09–1.78)	0.48 (0.20–0.73)	0.65 (0.26–1.41)	0.73 (0.29–1.83)
	C > T mutation at CpG sites	Low	low–medium	medium	medium	medium	medium
Copy Number	Purity (ABSOLUTE), median (range)	0.44 (0.18–0.8)[b]	0.46 (0.17–0.89)[b]	0.59 (0.16–0.92)	0.83 (0.36–0.92)	0.84 (0.47–0.93)	0.81 (0.4–0.91)
	Ploidy (ABSOLUTE), median (range)	3.01 (2.73–3.55)[b]	3.09 (2.67–4.97)[b]	2.84 (2.36–4.54)	2.67 (2.2–2.99)	2.78 (2.44–4.66)	2.8 (2.45–3.86)
	i(12p), no. (%)	12 (60)[b]	37 (80)[b]	27 (100)	16 (100)	13 (100)	9 (100)
	12p copies, median (range)	5 (4–8)[b]	7 (4–14)[b]	8 (5–12)	7 (4–9)	7 (5–17)	7 (5–7)
miRNA	miR 19q13.42 cluster	Low	low	high	low	low	low
	miR-371a-3p	High	high	high	low	moderate	high
	miR-375	Low	low	moderate	high	high	high

(*Continued on next page*)

Table 1. *Continued*

		Seminoma		NSGCT-EC	NSGCT-Other		
		Seminoma *KIT* Mutant	Seminoma *KIT* Wild-Type	EC	Teratoma	Yolk Sac	Mixed NSGCTs
KIT/KRAS Pathway	*KIT* mutations, no. (%)	25 (100)	0	0	0	0	0
	KIT copies, median (range)	3 (2–8)[b]	2 (2–12)[b]	2 (2–16)	2 (2–4)	2 (2–6)	2 (2–3)
	KIT mRNA	High	high	low	low	low	low
	KIT protein	High	high	low	low	low	low
	KITLG mRNA	Low	low	moderate	high	high	high
	KRAS mutations, no. (%)	4 (15.4)	14 (29.8)	0	1 (6.3)[c]	0	0
	KRAS copies, median (range)	5 (4–8)[b]	7 (4–≥60)[b]	7 (5–13)	6.5 (4–9)	8 (5–17)	7 (5–53)[d]
	KRAS mRNA	Low	high	high	low	low	low
	CBL copies, average (range)	2.1 (1–3)[b]	1.6 (1–3)[b]	2.1 (1–3)	1.9 (1–2)	2.4 (2–4)	2.1 (1–3)
	miR-222-3p	low	low	high	low	moderate	high

See also Tables S1 and S2.[a]Missing data for cryptorchidism (seminoma WT n = 6, Mixed Nseminoma n = 1).
[b]Six samples with low purity and inability to estimate copy number (5 KIT mutations, 1 KIT WT). Samples excluded from analysis.
[c]KRAS mutation in mature teratoma-dominant sample with 30% seminoma component.
[d]Sample with 53 copies of KRAS had 55% seminoma component.

and epigenetic defects likely occur before the re-establishment of imprinting methylation (i.e., *in utero*).

Treatment refractory TGCT is rare and mortality is now low, but late effects of chemotherapy and morbidity associated with surgery remain a clinical challenge that several of our results may help to address. Despite considerable effort (Gilbert et al., 2016; Vergouwe et al., 2003), a need remains to identify, among patients with stage I NSGCTs, the 50%–70% of men without occult metastases who could be cured by orchiectomy alone. A panel interrogating circulating *miR-371* plus *miR-375* is envisioned for identifying patients free from residual disease following orchiectomy, who could be spared adjuvant chemotherapy from which they would receive no benefit. *miR-371*, already proposed for this purpose (Syring et al., 2015), was highly expressed in seminomas and EC, but expression was low in yolk sac tumors and minimal in teratomas. We found *miR-375* to be highly expressed in teratomas and yolk sac tumors, for which it is a promising serum marker because circulating levels are reportedly low in healthy young men (Zhang et al., 2015). *miR-375* alone also is a promising marker for identifying, among patients with residual masses ≥1 cm following chemotherapy for stage II tumors, the 55%–60% whose masses contain only scar tissue (Daneshmand et al., 2012). These patients presently undergo extraordinarily invasive surgery because they cannot be distinguished before the procedure from patients whose masses harbor teratoma cells, requiring surgery to achieve cure. Translational studies to validate these miRNAs as predictive serum markers could start to fill these significant unmet needs.

Other results suggest strategies for targeted therapy. DNA methyltransferase inhibitors in NSGCT could reprogram the epigenome into a hypomethylated state and induce immunogenicity. A study showed that cells from refractory TGCTs are highly sensitive to guadecitabine (Albany et al., 2017). *BRCA1* and *RAD51C* promoter methylation in 35% of non-EC NSGCTs makes a significant proportion of these tumors candidates for treatment with PARP inhibitors (Cavallo et al., 2012). Finally, recurrent epigenetic silencing of *DNAJC15/MCJ* in NGSCTs makes these genes candidate predictive markers, because their expression in breast and uterine cancer cells is reportedly associated with drug resistance (Fernández-Cabezudo et al., 2016).

We have provided a rich source of data from multiple platforms that describe a large set of well-characterized TGCTs. Integrative analysis identified numerous molecular features that distinguish each histology and reflect the histological composition of mixed tumors; it also identified molecularly defined subsets of seminomas associated with *KIT* mutations. These data afford a more complete view of previously articulated hypotheses, provide additional insights into mechanisms of TGCT tumorigenesis, and identify possible new approaches to the treatment of TGCTs.

EXPERIMENTAL PROCEDURES

Tumor tissue and normal whole-blood samples were obtained from patients at contributing centers with informed consent, according to their local institutional review boards (IRBs). Biospecimens were centrally processed, and DNA, RNA, and protein were distributed to TCGA analysis centers.

TCGA project management has collected the necessary human subjects documentation to ensure that the project complies with 45 CFR 46 (the "Common Rule"). The program has obtained documentation from every contributing clinical site to verify that IRB approval has been obtained to participate in TCGA. Such documented approval may include one or more of the following items:

- An IRB-approved protocol with informed consent specific to TCGA or a substantially similar program. In the latter case, if the protocol was not TCGA specific, the clinical site's principal investigator (PI) provided a further finding from the IRB that the already-approved protocol was sufficient to participate in TCGA.
- A TCGA-specific IRB waiver has been granted.
- A TCGA-specific letter that the IRB considers one of the exemptions in 45 CFR 46 to be applicable. The two most common exemptions cited were that the research falls under 46.102(f)(2) or 46.101(b)(4). Both exempt requirements for informed consent because the received data and material do not contain directly identifiable private information.
- A TCGA-specific letter that the IRB does not consider the use of these data and materials to be human subjects research. This was most common for collections in which the donors were deceased.

This study included 137 primary TGCTs and matched germline control DNA obtained from 133 male patients. The median age of diagnosis was 31 years, with a range of 14–67. Patient tumor histology was classified according to a consensus of expert pathologists. Molecular and genomic data were collected using reverse phase protein arrays (RPPAs), whole-exome DNA sequencing, RNA-seq, miRNA sequencing, DNA methylation arrays, and SNP arrays for copy number analysis. Detailed methods are provided in the Supplemental Experimental Procedures.

DATA AND SOFTWARE AVAILABILITY

The raw data, processed data, and clinical data reported in this paper can be found at the legacy archive of the Genomic Data Commons (GDC) of the National Cancer Institute (https://portal.gdc.cancer.gov/legacy-archive/search/f) and the publication page (https://gdc.cancer.gov/about-data/publications/). TCGA data also can be explored through the Broad Institute FireBrowse portal (http://gdac.broadinstitute.org) and the Memorial Sloan Kettering Cancer Center cBioPortal (http://www.cbioportal.org).

SUPPLEMENTAL INFORMATION

Supplemental Information includes Supplemental Experimental Procedures, seven figures, and seven tables and can be found with this article online at https://doi.org/10.1016/j.celrep.2018.05.039.

ACKNOWLEDGMENTS

We thank the patients who contributed to this study. This project is supported by the following NIH grants: U54 HG003273, U54 HG003067, U54 HG003079, U24 CA143799, U24 CA143835, U24 CA143840, U24 CA143843, U24 CA143845, U24 CA143848, U24 CA143858, U24 CA143866, U24 CA143867, U24 CA143882, U24 CA143883, U24 CA144025, and P30 CA016672.

AUTHOR CONTRIBUTIONS

Project Leaders, V.K.C. and K.A.H.; Manuscript Coordinators, J.S. and D.P.H.; Data Coordinator, J.Z.; Project Oversight, J.C.Z., C.M.H., I.F., and J.Z.; Disease Experts, F.M.C., V.K.C., D.R.F., G. Godoy, J.J., T.K., D.L., R.M., K.L.N., V.E.R., C.S.S., S.K.T., and D.O.V.; Biospecimen Core Resource, K.M.L. and T.M.L.; DNA Sequence Analysis, L.W., K.K., L.X., and D.A.W.; Copy Number Analysis, J.S., A.D.C., G. Getz, and M.M.; DNA Methylation, H.S., W.Z., and P.W.L.; Protein Analysis, A.M.H. and R.A.; Germline SNP Analysis, V.T., J.P., L.C.P., and K.L.N.; miRNA Analysis, R.B., A.J.M., and D.B.; RNA Analysis, D.P.H. and K.A.H.; Pathway and Integration Analysis, J.A., J.C., G.G., D.I.H., M.L., Y.N., M.S.N., F.S.-V., N.S., J.M.S., and H.Z. Drafting and Reviewing Manuscript, all authors.

DECLARATION OF INTERESTS

A.C. and M.M. receive research funding from Bayer AG. J.M.S. is a founder and part owner of Five3 Genomics LLC and a member of its scientific advisory board, which owns the license to PARADIGM software.

Received: August 18, 2017
Revised: March 9, 2018
Accepted: May 14, 2018
Published: June 12, 2018

REFERENCES

Albany, C., Hever-Jardine, M.P., von Herrmann, K.M., Yim, C.Y., Tam, J., Warzecha, J.M., Shin, L., Bock, S.E., Curran, B.S., Chaudhry, A.S., et al. (2017). Refractory testicular germ cell tumors are highly sensitive to the second generation DNA methylation inhibitor guadecitabine. Oncotarget *8*, 2949–2959.

Alexandrov, L.B., Nik-Zainal, S., Wedge, D.C., Aparicio, S.A., Behjati, S., Biankin, A.V., Bignell, G.R., Bolli, N., Borg, A., Børresen-Dale, A.L., et al.; Australian Pancreatic Cancer Genome Initiative; ICGC Breast Cancer Consortium; ICGC MMML-Seq Consortium; ICGC PedBrain (2013). Signatures of mutational processes in human cancer. Nature *500*, 415–421.

Almeida, L.G., Sakabe, N.J., deOliveira, A.R., Silva, M.C., Mundstein, A.S., Cohen, T., Chen, Y.T., Chua, R., Gurung, S., Gnjatic, S., et al. (2009). CTdatabase: a knowledge-base of high-throughput and curated data on cancer-testis antigens. Nucleic Acids Res. *37*, D816–D819.

Banks, K., Tuazon, E., Berhane, K., Koh, C.J., De Filippo, R.E., Chang, A., Kim, S.S., Daneshmand, S., Davis-Dao, C., Lewinger, J.P., et al. (2013). Cryptorchidism and testicular germ cell tumors: comprehensive meta-analysis reveals that association between these conditions diminished over time and is modified by clinical characteristics. Front. Endocrinol. (Lausanne) *3*, 182.

Brabrand, S., Johannessen, B., Axcrona, U., Kraggerud, S.M., Berg, K.G., Bakken, A.C., Bruun, J., Fosså, S.D., Lothe, R.A., Lehne, G., and Skotheim, R.I. (2015). Exome sequencing of bilateral testicular germ cell tumors suggests independent development lineages. Neoplasia *17*, 167–174.

Cardoso, H.J., Figueira, M.I., Correia, S., Vaz, C.V., and Socorro, S. (2014). The SCF/c-KIT system in the male: survival strategies in fertility and cancer. Mol. Reprod. Dev. *81*, 1064–1079.

Carter, S.L., Cibulskis, K., Helman, E., McKenna, A., Shen, H., Zack, T., Laird, P.W., Onofrio, R.C., Winckler, W., Weir, B.A., et al. (2012). Absolute quantification of somatic DNA alterations in human cancer. Nat. Biotechnol. *30*, 413–421.

Cavallo, F., Graziani, G., Antinozzi, C., Feldman, D.R., Houldsworth, J., Bosl, G.J., Chaganti, R.S., Moynahan, M.E., Jasin, M., and Barchi, M. (2012). Reduced proficiency in homologous recombination underlies the high sensitivity of embryonal carcinoma testicular germ cell tumors to cisplatin and poly (adp-ribose) polymerase inhibition. PLoS One *7*, e51563.

Chiappinelli, K.B., Strissel, P.L., Desrichard, A., Li, H., Henke, C., Akman, B., Hein, A., Rote, N.S., Cope, L.M., Snyder, A., et al. (2015). Inhibiting DNA methylation causes an interferon response in cancer via dsRNA including endogenous retroviruses. Cell *162*, 974–986.

Chung, C.C., Kanetsky, P.A., Wang, Z., Hildebrandt, M.A., Koster, R., Skotheim, R.I., Kratz, C.P., Turnbull, C., Cortessis, V.K., Bakken, A.C., et al. (2013). Meta-analysis identifies four new loci associated with testicular germ cell tumor. Nat. Genet. *45*, 680–685.

Cortessis, V. (2003). Epidemiologic insights into the occurrence and causes of testicular cancer. In American Cancer Society Atlas of Clinical Oncology: Germ Cell Tumors, D. Raghavan, ed. (B. C. Decker).

Court, F., Tayama, C., Romanelli, V., Martin-Trujillo, A., Iglesias-Platas, I., Okamura, K., Sugahara, N., Simón, C., Moore, H., Harness, J.V., et al. (2014). Genome-wide parent-of-origin DNA methylation analysis reveals the intricacies

of human imprinting and suggests a germline methylation-independent mechanism of establishment. Genome Res. *24*, 554–569.

Covington, K., Shinbrot, E., and Wheeler, D.A. (2016). Mutation signatures reveal biological processes in human cancer. bioRxiv. https://doi.org/10.1101/036541.

Cutcutache, I., Suzuki, Y., Tan, I.B., Ramgopal, S., Zhang, S., Ramnarayanan, K., Gan, A., Lee, H.H., Tay, S.T., Ooi, A., et al. (2015). Exome-wide sequencing shows low mutation rates and identifies novel mutated genes in seminomas. Eur. Urol. *68*, 77–83.

Daneshmand, S., Albers, P., Fosså, S.D., Heidenreich, A., Kollmannsberger, C., Krege, S., Nichols, C., Oldenburg, J., and Wood, L. (2012). Contemporary management of postchemotherapy testis cancer. Eur. Urol. *62*, 867–876.

De Carvalho, D.D., Sharma, S., You, J.S., Su, S.F., Taberlay, P.C., Kelly, T.K., Yang, X., Liang, G., and Jones, P.A. (2012). DNA methylation screening identifies driver epigenetic events of cancer cell survival. Cancer Cell *21*, 655–667.

Dieckmann, K.P., Radtke, A., Spiekermann, M., Balks, T., Matthies, C., Becker, P., Ruf, C., Oing, C., Oechsle, K., Bokemeyer, C., et al. (2017). Serum levels of microRNA miR-371a-3p: a sensitive and specific new biomarker for germ cell tumours. Eur. Urol. *71*, 213–220.

Feldman, D.R., Iyer, G., Van Alstine, L., Patil, S., Al-Ahmadie, H., Reuter, V.E., Bosl, G.J., Chaganti, R.S., and Solit, D.B. (2014). Presence of somatic mutations within PIK3CA, AKT, RAS, and FGFR3 but not BRAF in cisplatin-resistant germ cell tumors. Clin. Cancer Res. *20*, 3712–3720.

Fernández-Cabezudo, M.J., Faour, I., Jones, K., Champagne, D.P., Jaloudi, M.A., Mohamed, Y.A., Bashir, G., Almarzooqi, S., Albawardi, A., Hashim, M.J., et al. (2016). Deficiency of mitochondrial modulator MCJ promotes chemoresistance in breast cancer. JCI Insight *1*, e86873.

Fosså, S.D., Chen, J., Schonfeld, S.J., McGlynn, K.A., McMaster, M.L., Gail, M.H., and Travis, L.B. (2005). Risk of contralateral testicular cancer: a population-based study of 29,515 U.S. men. J. Natl. Cancer Inst. *97*, 1056–1066.

Frigyesi, A., Gisselsson, D., Hansen, G.B., Soller, M., Mitelman, F., and Höglund, M. (2004). A model for karyotypic evolution in testicular germ cell tumors. Genes Chromosomes Cancer *40*, 172–178.

Geurts van Kessel, A., van Drunen, E., de Jong, B., Oosterhuis, J.W., Langeveld, A., and Mulder, M.P. (1989). Chromosome 12q heterozygosity is retained in i(12p)-positive testicular germ cell tumor cells. Cancer Genet. Cytogenet. *40*, 129–134.

Gilbert, D.C., Al-Saadi, R., Thway, K., Chandler, I., Berney, D., Gabe, R., Stenning, S.P., Sweet, J., Huddart, R., and Shipley, J.M. (2016). Defining a new prognostic index for stage I nonseminomatous germ cell tumors using CXCL12 expression and proportion of embryonal carcinoma. Clin. Cancer Res. *22*, 1265–1273.

Gimenez, J., Montgiraud, C., Pichon, J.P., Bonnaud, B., Arsac, M., Ruel, K., Bouton, O., and Mallet, F. (2010). Custom human endogenous retroviruses dedicated microarray identifies self-induced HERV-W family elements reactivated in testicular cancer upon methylation control. Nucleic Acids Res. *38*, 2229–2246.

Gits, C.M., van Kuijk, P.F., Jonkers, M.B., Boersma, A.W., van Ijcken, W.F., Wozniak, A., Sciot, R., Rutkowski, P., Schöffski, P., Taguchi, T., et al. (2013). MiR-17-92 and miR-221/222 cluster members target KIT and ETV1 in human gastrointestinal stromal tumours. Br. J. Cancer *109*, 1625–1635.

Honecker, F., Stoop, H., Mayer, F., Bokemeyer, C., Castrillon, D.H., Lau, Y.F., Looijenga, L.H., and Oosterhuis, J.W. (2006). Germ cell lineage differentiation in non-seminomatous germ cell tumours. J. Pathol. *208*, 395–400.

Honorio, S., Agathanggelou, A., Wernert, N., Rothe, M., Maher, E.R., and Latif, F. (2003). Frequent epigenetic inactivation of the RASSF1A tumour suppressor gene in testicular tumours and distinct methylation profiles of seminoma and nonseminoma testicular germ cell tumours. Oncogene *22*, 461–466.

Hvarness, T., Nielsen, J.E., Almstrup, K., Skakkebaek, N.E., Rajpert-De Meyts, E., and Claesson, M.H. (2013). Phenotypic characterisation of immune cell infiltrates in testicular germ cell neoplasia. J. Reprod. Immunol. *100*, 135–145.

Jørgensen, N., Müller, J., Giwercman, A., Visfeldt, J., Møller, H., and Skakkebaek, N.E. (1995). DNA content and expression of tumour markers in germ cells adjacent to germ cell tumours in childhood: probably a different origin for infantile and adolescent germ cell tumours. J. Pathol. *176*, 269–278.

Kanetsky, P.A., Mitra, N., Vardhanabhuti, S., Li, M., Vaughn, D.J., Letrero, R., Ciosek, S.L., Doody, D.R., Smith, L.M., Weaver, J., et al. (2009). Common variation in KITLG and at 5q31.3 predisposes to testicular germ cell cancer. Nat. Genet. *41*, 811–815.

Koul, S., Houldsworth, J., Mansukhani, M.M., Donadio, A., McKiernan, J.M., Reuter, V.E., Bosl, G.J., Chaganti, R.S., and Murty, V.V. (2002). Characteristic promoter hypermethylation signatures in male germ cell tumors. Mol. Cancer *1*, 8.

Lawrence, M.S., Stojanov, P., Mermel, C.H., Robinson, J.T., Garraway, L.A., Golub, T.R., Meyerson, M., Gabriel, S.B., Lander, E.S., and Getz, G. (2014). Discovery and saturation analysis of cancer genes across 21 tumour types. Nature *505*, 495–501.

Lister, R., Pelizzola, M., Dowen, R.H., Hawkins, R.D., Hon, G., Tonti-Filippini, J., Nery, J.R., Lee, L., Ye, Z., Ngo, Q.M., et al. (2009). Human DNA methylomes at base resolution show widespread epigenomic differences. Nature *462*, 315–322.

Litchfield, K., Summersgill, B., Yost, S., Sultana, R., Labreche, K., Dudakia, D., Renwick, A., Seal, S., Al-Saadi, R., Broderick, P., et al. (2015). Whole-exome sequencing reveals the mutational spectrum of testicular germ cell tumours. Nat. Commun. *6*, 5973.

Litchfield, K., Levy, M., Huddart, R.A., Shipley, J., and Turnbull, C. (2016). The genomic landscape of testicular germ cell tumours: from susceptibility to treatment. Nat. Rev. Urol. *13*, 409–419.

Martinelli, C.M.D.S., Lengert, A.V.H., Cárcano, F.M., Silva, E.C.A., Brait, M., Lopes, L.F., and Vidal, D.O. (2016). MGMT and CALCA promoter methylation are associated with poor prognosis in testicular germ cell tumor patients. Oncotarget *8*, 50608–50617.

McIntyre, A., Summersgill, B., Jafer, O., Rodriguez, S., Zafarana, G., Oosterhuis, J.W., Gillis, A.J., Looijenga, L., Cooper, C., Huddart, R., et al. (2004). Defining minimum genomic regions of imbalance involved in testicular germ cell tumors of adolescents and adults through genome wide microarray analysis of cDNA clones. Oncogene *23*, 9142–9147.

McIntyre, A., Summersgill, B., Grygalewicz, B., Gillis, A.J., Stoop, J., van Gurp, R.J., Dennis, N., Fisher, C., Huddart, R., Cooper, C., et al. (2005). Amplification and overexpression of the KIT gene is associated with progression in the seminoma subtype of testicular germ cell tumors of adolescents and adults. Cancer Res. *65*, 8085–8089.

Moch, H., Cubilla, A.L., Humphrey, P.A., Reuter, V.E., and Ulbright, T.M. (2016). The 2016 WHO Classification of Tumours of the Urinary System and Male Genital Organs-Part A: Renal, Penile, and Testicular Tumours. Eur. Urol. *70*, 93–105.

Mostert, M., Rosenberg, C., Stoop, H., Schuyer, M., Timmer, A., Oosterhuis, W., and Looijenga, L. (2000). Comparative genomic and in situ hybridization of germ cell tumors of the infantile testis. Lab. Invest. *80*, 1055–1064.

Netto, G.J., Nakai, Y., Nakayama, M., Jadallah, S., Toubaji, A., Nonomura, N., Albadine, R., Hicks, J.L., Epstein, J.I., Yegnasubramanian, S., et al. (2008). Global DNA hypomethylation in intratubular germ cell neoplasia and seminoma, but not in nonseminomatous male germ cell tumors. Mod. Pathol. *21*, 1337–1344.

Newton, Y., Novak, A.M., Swatloski, T., McColl, D.C., Chopra, S., Graim, K., Weinstein, A.S., Baertsch, R., Salama, S.R., Ellrott, K., et al. (2017). TumorMap: exploring the molecular similarities of cancer samples in an interactive portal. Cancer Res. *77*, e111–e114.

Ottesen, A.M., Skakkebaek, N.E., Lundsteen, C., Leffers, H., Larsen, J., and Rajpert-De Meyts, E. (2003). High-resolution comparative genomic hybridization detects extra chromosome arm 12p material in most cases of carcinoma in situ adjacent to overt germ cell tumors, but not before the invasive tumor development. Genes Chromosomes Cancer *38*, 117–125.

Parker, C., Milosevic, M., Panzarella, T., Banerjee, D., Jewett, M., Catton, C., Tew-George, B., Gospodarowicz, M., and Warde, P. (2002). The prognostic

significance of the tumour infiltrating lymphocyte count in stage I testicular seminoma managed by surveillance. Eur. J. Cancer *38*, 2014–2019.

Roulois, D., Loo Yau, H., Singhania, R., Wang, Y., Danesh, A., Shen, S.Y., Han, H., Liang, G., Jones, P.A., Pugh, T.J., et al. (2015). DNA-demethylating agents target colorectal cancer cells by inducing viral mimicry by endogenous transcripts. Cell *162*, 961–973.

Shen, H., and Laird, P.W. (2013). Interplay between the cancer genome and epigenome. Cell *153*, 38–55.

Smiraglia, D.J., Szymanska, J., Kraggerud, S.M., Lothe, R.A., Peltomäki, P., and Plass, C. (2002). Distinct epigenetic phenotypes in seminomatous and nonseminomatous testicular germ cell tumors. Oncogene *21*, 3909–3916.

Stang, A., Jansen, L., Trabert, B., Rusner, C., Eberle, A., Katalinic, A., Emrich, K., Holleczek, B., and Brenner, H.; GEKID Cancer Survival Working Group (2013). Survival after a diagnosis of testicular germ cell cancers in Germany and the United States, 2002-2006: a high resolution study by histology and age. Cancer Epidemiol. *37*, 492–497.

Summersgill, B., Osin, P., Lu, Y.J., Huddart, R., and Shipley, J. (2001). Chromosomal imbalances associated with carcinoma in situ and associated testicular germ cell tumours of adolescents and adults. Br. J. Cancer *85*, 213–220.

Syring, I., Bartels, J., Holdenrieder, S., Kristiansen, G., Müller, S.C., and Ellinger, J. (2015). Circulating serum miRNA (miR-367-3p, miR-371a-3p, miR-372-3p and miR-373-3p) as biomarkers in patients with testicular germ cell cancer. J. Urol. *193*, 331–337.

Taylor-Weiner, A., Zack, T., O'Donnell, E., Guerriero, J.L., Bernard, B., Reddy, A., Han, G.C., AlDubayan, S., Amin-Mansour, A., Schumacher, S.E., et al. (2016). Genomic evolution and chemoresistance in germ-cell tumours. Nature *540*, 114–118.

The Cancer Genome Atlas Research Network (2011). Integrated genomic analyses of ovarian carcinoma. Nature *474*, 609–615.

Thomas, J.P., Davis-Dao, C., Lewinger, J.P., Siegmund, K.D., Hamilton, A.S., Daneshmand, S., Skinner, E.C., Thomas, D.C., and Cortessis, V.K. (2013). Null association between histology of first and second primary malignancies in men with bilateral testicular germ cell tumors. Am. J. Epidemiol. *178*, 1240–1245.

Tian, Q., Frierson, H.F., Jr., Krystal, G.W., and Moskaluk, C.A. (1999). Activating c-kit gene mutations in human germ cell tumors. Am. J. Pathol. *154*, 1643–1647.

Vergouwe, Y., Steyerberg, E.W., Eijkemans, M.J., Albers, P., and Habbema, J.D. (2003). Predictors of occult metastasis in clinical stage I nonseminoma: a systematic review. J. Clin. Oncol. *21*, 4092–4099.

Wang, L., Yamaguchi, S., Burstein, M.D., Terashima, K., Chang, K., Ng, H.K., Nakamura, H., He, Z., Doddapaneni, H., Lewis, L., et al. (2014). Novel somatic and germline mutations in intracranial germ cell tumours. Nature *511*, 241–245.

Wang, Z., McGlynn, K.A., Rajpert-De Meyts, E., Bishop, D.T., Chung, C.C., Dalgaard, M.D., Greene, M.H., Gupta, R., Grotmol, T., Haugen, T.B., et al.; Testicular Cancer Consortium (2017). Meta-analysis of five genome-wide association studies identifies multiple new loci associated with testicular germ cell tumor. Nat. Genet. *49*, 1141–1147.

Widschwendter, M., Fiegl, H., Egle, D., Mueller-Holzner, E., Spizzo, G., Marth, C., Weisenberger, D.J., Campan, M., Young, J., Jacobs, I., and Laird, P.W. (2007). Epigenetic stem cell signature in cancer. Nat. Genet. *39*, 157–158.

Wilson, K.D., Venkatasubrahmanyam, S., Jia, F., Sun, N., Butte, A.J., and Wu, J.C. (2009). MicroRNA profiling of human-induced pluripotent stem cells. Stem Cells Dev. *18*, 749–758.

Zhang, H., Yang, H., Zhang, C., Jing, Y., Wang, C., Liu, C., Zhang, R., Wang, J., Zhang, J., Zen, K., et al. (2015). Investigation of microRNA expression in human serum during the aging process. J. Gerontol. A Biol. Sci. Med. Sci. *70*, 102–109.

Cell Reports, Volume *23*

Supplemental Information

Integrated Molecular Characterization

of Testicular Germ Cell Tumors

Hui Shen, Juliann Shih, Daniel P. Hollern, Linghua Wang, Reanne Bowlby, Satish K. Tickoo, Vésteinn Thorsson, Andrew J. Mungall, Yulia Newton, Apurva M. Hegde, Joshua Armenia, Francisco Sánchez-Vega, John Pluta, Louise C. Pyle, Rohit Mehra, Victor E. Reuter, Guilherme Godoy, Jeffrey Jones, Carl S. Shelley, Darren R. Feldman, Daniel O. Vidal, Davor Lessel, Tomislav Kulis, Flavio M. Cárcano, Kristen M. Leraas, Tara M. Lichtenberg, Denise Brooks, Andrew D. Cherniack, Juok Cho, David I. Heiman, Katayoon Kasaian, Minwei Liu, Michael S. Noble, Liu Xi, Hailei Zhang, Wanding Zhou, Jean C. ZenKlusen, Carolyn M. Hutter, Ina Felau, Jiashan Zhang, Nikolaus Schultz, Gad Getz, Matthew Meyerson, Joshua M. Stuart, The Cancer Genome Atlas Research Network, Rehan Akbani, David A. Wheeler, Peter W. Laird, Katherine L. Nathanson, Victoria K. Cortessis, and Katherine A. Hoadley

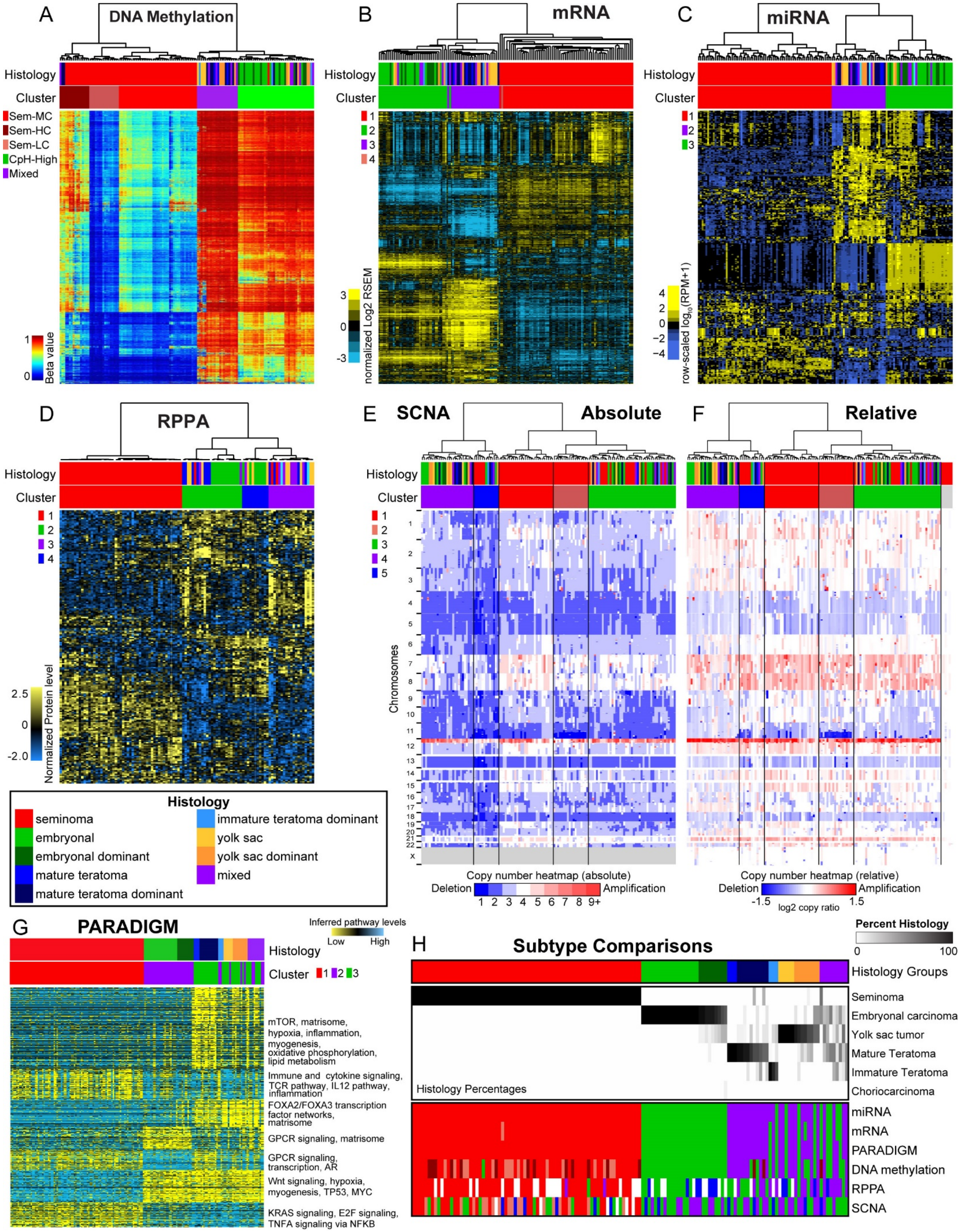
A
DNA Methylation
Histology
Cluster
Sem-MC
Sem-HC
Sem-LC
CpH-High
Mixed
Beta value
B
mRNA
normalized Log2 RSEM
C
miRNA
row-scaled log10(RPM+1)
D
RPPA
Normalized Protein level
Histology
seminoma
embryonal
embryonal dominant
mature teratoma
mature teratoma dominant
immature teratoma dominant
yolk sac
yolk sac dominant
mixed
E
SCNA
Absolute
Chromosomes
Copy number heatmap (absolute)
Deletion
Amplification
F
Relative
Copy number heatmap (relative)
log2 copy ratio
G
PARADIGM
Inferred pathway levels
Low
High
mTOR, matrisome, hypoxia, inflammation, myogenesis, oxidative phosphorylation, lipid metabolism
Immune and cytokine signaling, TCR pathway, IL12 pathway, inflammation
FOXA2/FOXA3 transcription factor networks, matrisome
GPCR signaling, matrisome
GPCR signaling, transcription, AR
Wnt signaling, hypoxia, myogenesis, TP53, MYC
KRAS signaling, E2F signaling, TNFA signaling via NFKB
H
Subtype Comparisons
Percent Histology
Histology Groups
Seminoma
Embryonal carcinoma
Yolk sac tumor
Mature Teratoma
Immature Teratoma
Choriocarcinoma
Histology Percentages
miRNA
mRNA
PARADIGM
DNA methylation
RPPA
SCNA

Figure S1. Related to Figure 1 and Table S1. Molecular classification of TGCT. A) DNA methylation, unsupervised clustering of 9,614 variably methylated autosomal CpG probes identifies five clusters predominantly separating histological types. **B) mRNA,** unsupervised consensus hierarchical clustering of 2,787 variably and highly expressed genes identified three main clusters with high concordance to histology types. **C) miRNA,** unsupervised hierarchical clustering of the top 25% most variable miRNA 5p or 3p strands identified 3 clusters. **D) Protein,** unsupervised clustering of RPPA data for 104 samples and 218 antibodies identified four clusters. **E,F) Copy number,** unsupervised hierarchical clustering of arm-level somatic copy number alterations separates 137 TGCT tumors into five groups. The heatmaps show absolute (total integer copies, **E**) and relative copy number (corrected to average sample ploidy, **F**), with chromosomes ordered from top to bottom. **G) Paradigm,** clustering of protein coding inferred pathway levels (IPLs) produced by PARADIGM integrating mRNA and copy number identified 3 subtypes. Distinct groups of genes are enriched in various pathways, annotated to the right of the gene group. **H)** Comparison of histology groups, individual histology percentages, and molecular subtypes.

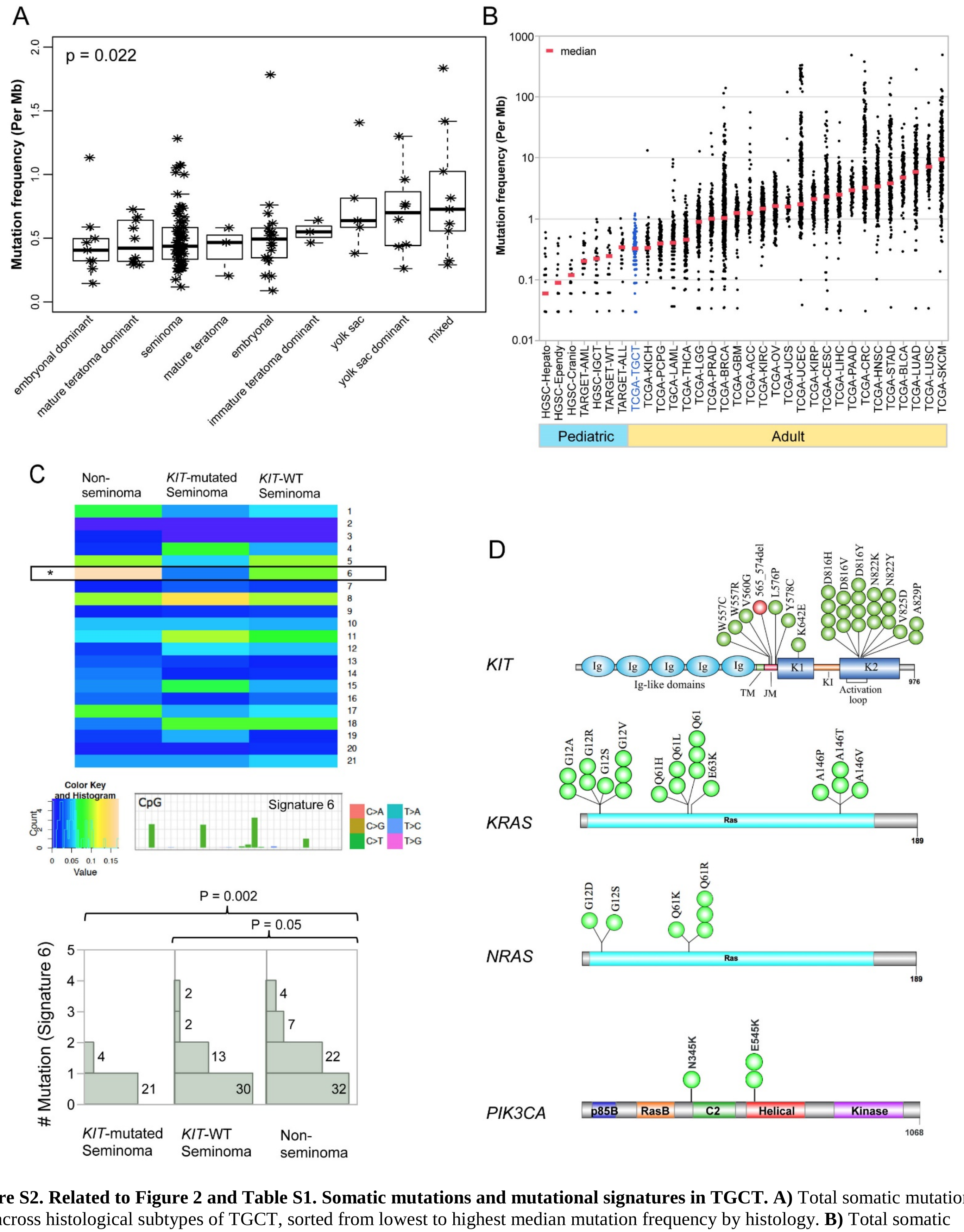

Figure S2. Related to Figure 2 and Table S1. Somatic mutations and mutational signatures in TGCT. A) Total somatic mutation rate across histological subtypes of TGCT, sorted from lowest to highest median mutation frequency by histology. **B)** Total somatic mutation rate across 7 pediatric and 25 adult tumor types, sorted from lowest to median mutation frequency. TGCT is highlighted in blue. **C)** Increased mutational signature 6 observed in non-seminomas. The heatmap shows the mutational signatures across three TGCT groups, defined by histology and *KIT* mutation status. Somatic mutations were pooled from tumors within the same group for signature analysis. Of 21 cancer mutation signatures (Covington and Wheeler, 2015), Signature 6 (C>T substitutions at CpG

dinucleotides suggestive of the number of cell replications) was the only one for which mutation frequencies were significantly different across groups. The middle panel shows the relative proportions of each base change that characterize Signature 6 displayed according to the 96 sequence contexts immediately 3’ and 5’ to the mutated base. Lower panel, somatic mutations attributable to Signature 6. The bar chart displays the number of somatic mutations in each sample with a Signature 6 mutation profile. P-values are calculated for each pair of groups using the Wilcoxon *T* test. D**)** Schematic representation of somatic mutations identified in *KIT*, *KRAS*, *NRAS*, and *PIK3CA*. Missense mutation, green circle; INDEL, red circle.

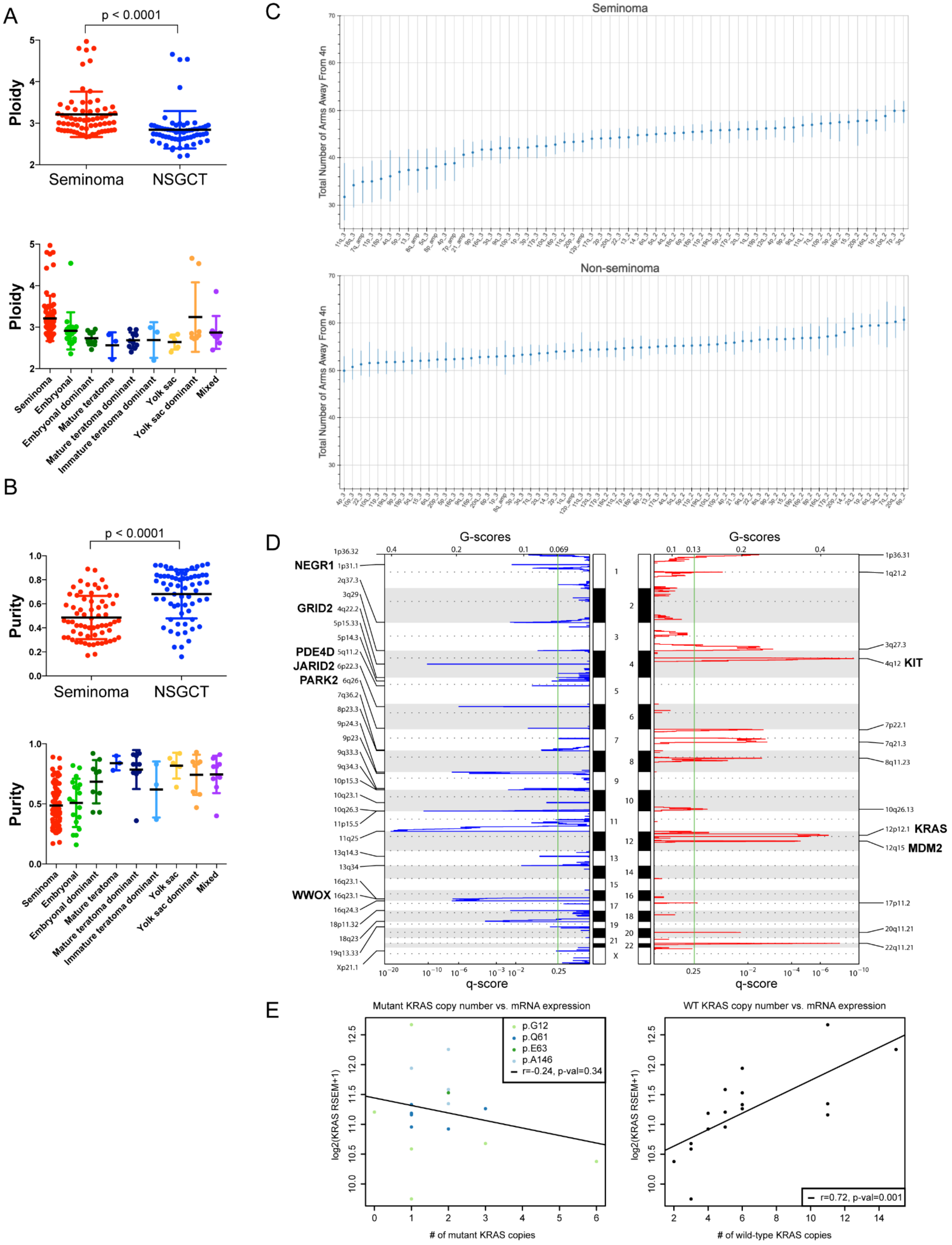
A
p < 0.0001
Ploidy
Seminoma
NSGCT
Ploidy
Seminoma
Embryonal
Embryonal dominant
Mature teratoma
Mature teratoma dominant
Immature teratoma dominant
Yolk sac
Yolk sac dominant
Mixed
B
p < 0.0001
Purity
Seminoma
NSGCT
Purity
Seminoma
Embryonal
Embryonal dominant
Mature teratoma
Mature teratoma dominant
Immature teratoma dominant
Yolk sac
Yolk sac dominant
Mixed
C
Seminoma
Total Number of Arms Away From 4n
Non-seminoma
Total Number of Arms Away From 4n
D
G-scores
G-scores
NEGR1
GRID2
PDE4D
JARID2
PARK2
WWOX
KIT
KRAS
MDM2
q-score
q-score
E
Mutant KRAS copy number vs. mRNA expression
p.G12
p.Q61
p.E63
p.A146
r=-0.24, p-val=0.34
log2(KRAS RSEM+1)
# of mutant KRAS copies
WT KRAS copy number vs. mRNA expression
r=0.72, p-val=0.001
log2(KRAS RSEM+1)
# of wild-type KRAS copies

Figure S3. Related to Figure 2 and Tables S1 and S3. Copy number alterations and characteristics differ across TGCT histology types. A) Tumor purity and **B)** ploidy across SCNA clusters by histology. **C)** Significant focal copy number alterations by GISTIC 2.0 analysis. Left: significantly amplified genomic regions; known oncogenes *KIT, KRAS*, and *MDM2* are within significant peaks as noted. Right: significantly deleted genomic regions; known fragile sites *NEGR1, GRID2, PDE4D, JARID2, PARK2,* and *WWOX* are within significant peaks as noted. **D)** Timing of arm-level events in groups of TGCT. Arm-level copy number alterations with at least seven occurrences across samples ordered from left to right by the mean "aneuploidy score" (total number of arms away from tetraploidy) of tumors possessing the alteration. Because tumors tend to gain aneuploidy or chromosomal instability over the course of tumorigenesis, "aneuploidy score" acts as a surrogate for relative timing, with events sorted from earliest to latest from left to right. The span of each vertical line represents bootstrapped 95% confidence intervals. **E)** DNA copies of wild-type *KRAS*, but not copies of mutant *KRAS*, are associated with *KRAS* gene expression. *KRAS* mRNA expression by the number of mutant (left) or wild-type (WT, right) *KRAS* copies present in each of the 17/19 tumors with copy number calls. Each tumor is colored by the identity of the mutant *KRAS* allele it possesses. Pearson's correlation and p-value are reported (right).

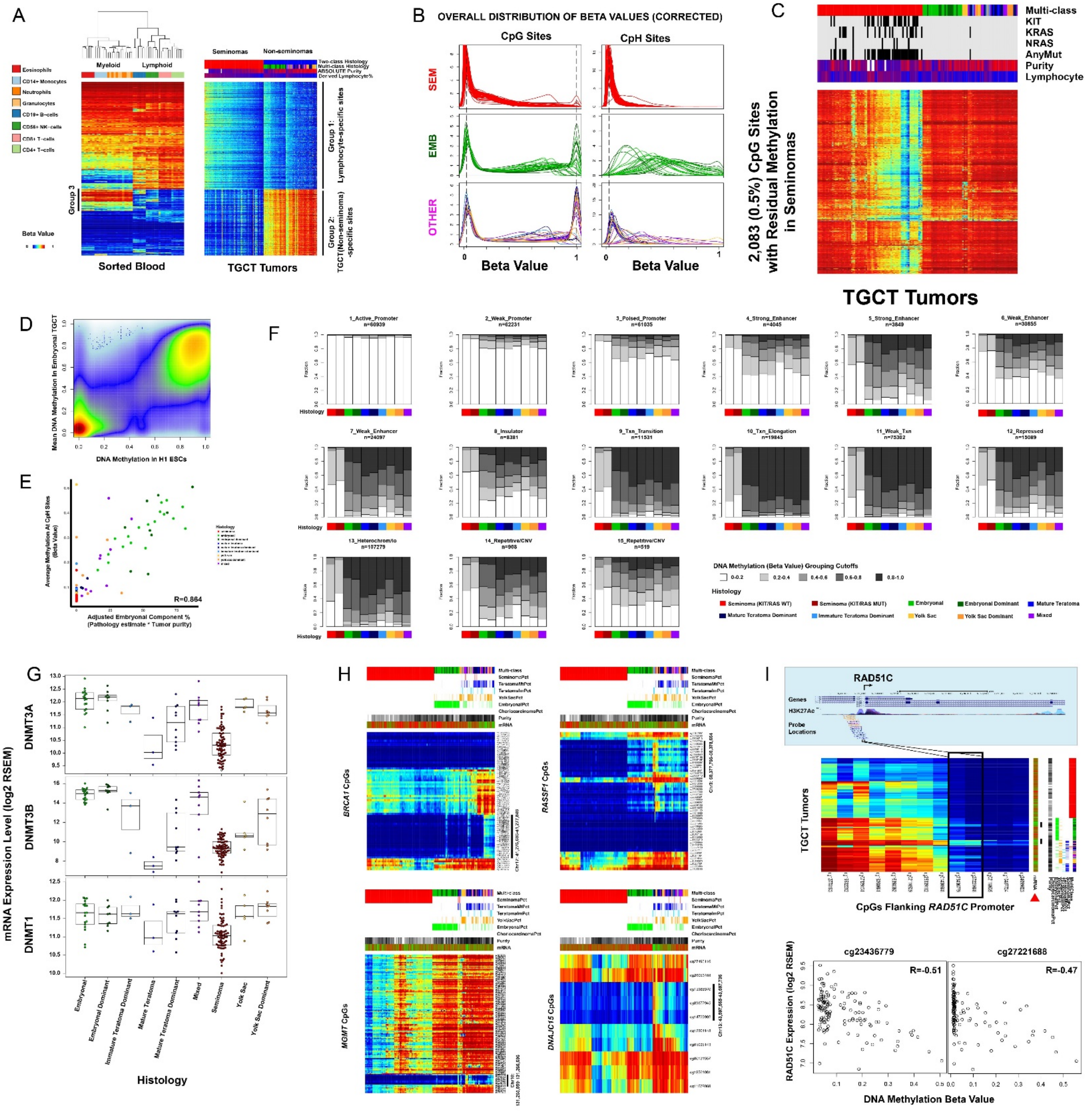

Figure S4. Related to Figure 4 and Table S1. ***DNA methylation patterns across TGCT*. A)** Lymphocyte-specific DNA methylation fingerprint is present in the TGCT tumors, especially seminomas. DNA methylation levels for 1,211 probes were plotted in the same order for sorted blood populations (left panel, samples ordered by hierarchical clustering) and TGCT (right panel, samples ordered by histology, then lymphocytic methylation signature). Lymphocytic-specific probes (Group 1, n=719 probes) were chosen by selecting CpG sites with a high DNA methylation level in lymphocytes (mean beta value >0.7) and low in non-seminomas (mean beta value <0.3). As a control, TGCT (Non-seminoma)-specific probes (Group 2, n=472) were chosen by selecting CpG sites with low DNA methylation level in lymphocytes (<0.3) and high DNA methylation level in non-seminomas (>0.7). Within Group 2, the myeloid DNA-methylation signature (Group 3) was not observed in seminoma. The kernel-smoothed DNA methylation distribution at these sites is used as a surrogate for lymphocyte fraction and is plotted as the bottom column-side color bar on the right panel (labeled

‘Derived Lymphocyte %’; blue to red – low to high lymphocyte %). **B)** Overall CpH and CpG methylation distributions after correction for lymphocyte methylation reveal almost complete lack of methylation in seminomas but not in other subtypes. Methylated peaks in seminomas disappear after lymphocyte methylation subtraction, while this correction does not eliminate the methylated peaks in non-seminomas. The color of each smoothed density plot lines indicates the annotated histology of the corresponding sample. **C)** Residual DNA methylation in seminomas differs based on *KIT*/*RAS* mutation status before correction for lymphocyte methylation. A heatmap shows 2,083 (0.5%) loci (rows) with residual methylation in seminomas (columns). Blue to red indicates 0% to 100% methylation, before correction for lymphocyte methylation. Top color bars annotate the histology of each tumor and mutation status in *KIT/KRAS/NRAS* (black – mutants; gray – wildtype). **D)** Comparison of global DNA methylation in embryonal and embryonal dominant TGCT tumors and H1 ESC. DNA methylation levels in H1 ESCs from ENCODE (x-axis) for all HM450 probes are plotted against the average DNA methylation levels in embryonal and embryonal dominant TGCT tumors (y-axis). Color indicates smoothed density of CpGs/probes, where blue indicates lowest and red indicates highest density. **E)** Average CpH methylation highly correlates with percent embryonal carcinoma component. The x-axis represents adjusted embryonal carcinoma component percentage, calculated as pathologists’ estimate of embryonal carcinoma component percentage within the tumor compartment, multiplied by ABSOLUTE-based tumor purity estimate (to derive the percentage of embryonal carcinoma component in the bulk DNA). The y-axis denotes the average CpH methylation level for the corresponding tumor, while the color of the tumor indicates its histological classification. Pearson’s correlation coefficient (R) between the two measurements is denoted in the lower right. **F)** Distribution of DNA methylation in the genome. The genome is segmented into different chromatin states based on the ChromHMM algorithm {Ernst, 2012 #81} applied to ChIP-seq results obtained from H1 embryonic stem cells (ESCs). Probes/CpGs that overlap with each chromatin state are plotted as one panel and the total number of probes for each panel is indicated below the name of the corresponding chromatin state. TGCT tumors with sufficient purity (>0.7 based on ASBOLUTE estimates) are grouped into ten histology groups and within each group, the fractions of CpGs with median DNA methylation levels within five consecutive ranges (0-0.2, 0.2-0.4,0.4-0.6,0.6-0.8,0.8-1.0) are shown with bar charts. Seminomas are split into KIT/RAS wildtype (WT) cases and mutant cases (MUT). **G)** The *de novo* DNA methyltransferases DNMT3A/B are overexpressed in embyronal and embryonal dominant TGCT tumors. mRNA expression levels (log2 RSEM) for the *de novo* DNA methyltransferases DNMT3A, DNMT3B and maintenance DNA methyltransferase DNMT1 are plotted as boxplots, with dot plots showing each tumor. **H)** Promoter hypermethylation and/or epigenetic silencing of *BRCA1*, *RASSF1*, *DNAJC15*, and *MGMT* (clockwise from top right). DNA methylation levels (blue – red: low to high level of DNA methylation) of all probes located within (-1500, +200) bp of all transcripts mapped to each gene (rows; sorted by increasing genomic location from bottom to top) for each sample (columns) are shown as a heatmap. Selected features of each sample are plotted as column-side color bars with the same notations used in other figures. In particular, an mRNA expression bar (green – red: low – high level of expression) is plotted, to show that samples with hypermethylation across the region of interest have low expression. A vertical bar indicates the location of the region showing cancer-specific hypermethylation together with the corresponding hg19/GRCh37 coordinates. **I)** Epigenetic silencing of *RAD51C*. Upper panel – a genomic view of the *RAD51C* gene. H3K27Ac peaks indicate two separate regulatory elements. Locations of probes plotted in Panel B are indicated in relation to the presumed *RAD51C* promoter. Middle panel – heatmap showing the DNA methylation level (blue – red: low to high level of DNA methylation) of all probes located within +/- 1500bp of the *RAD51C* TSS. Hypermethylation at two CpGs (black box) from the first H3K27Ac peak closest to the TSS is observed. Samples with methylation at these sites also have low mRNA expression (mRNA bar indicated by a red triangle; green to red color represent low to high level of mRNA expression). Bottom panel – scatterplots of the *RAD51C* mRNA level (y-axis) versus DNA methylation level (x-axis) at these two CpG sites demonstrate decreased expression with increased methylation.

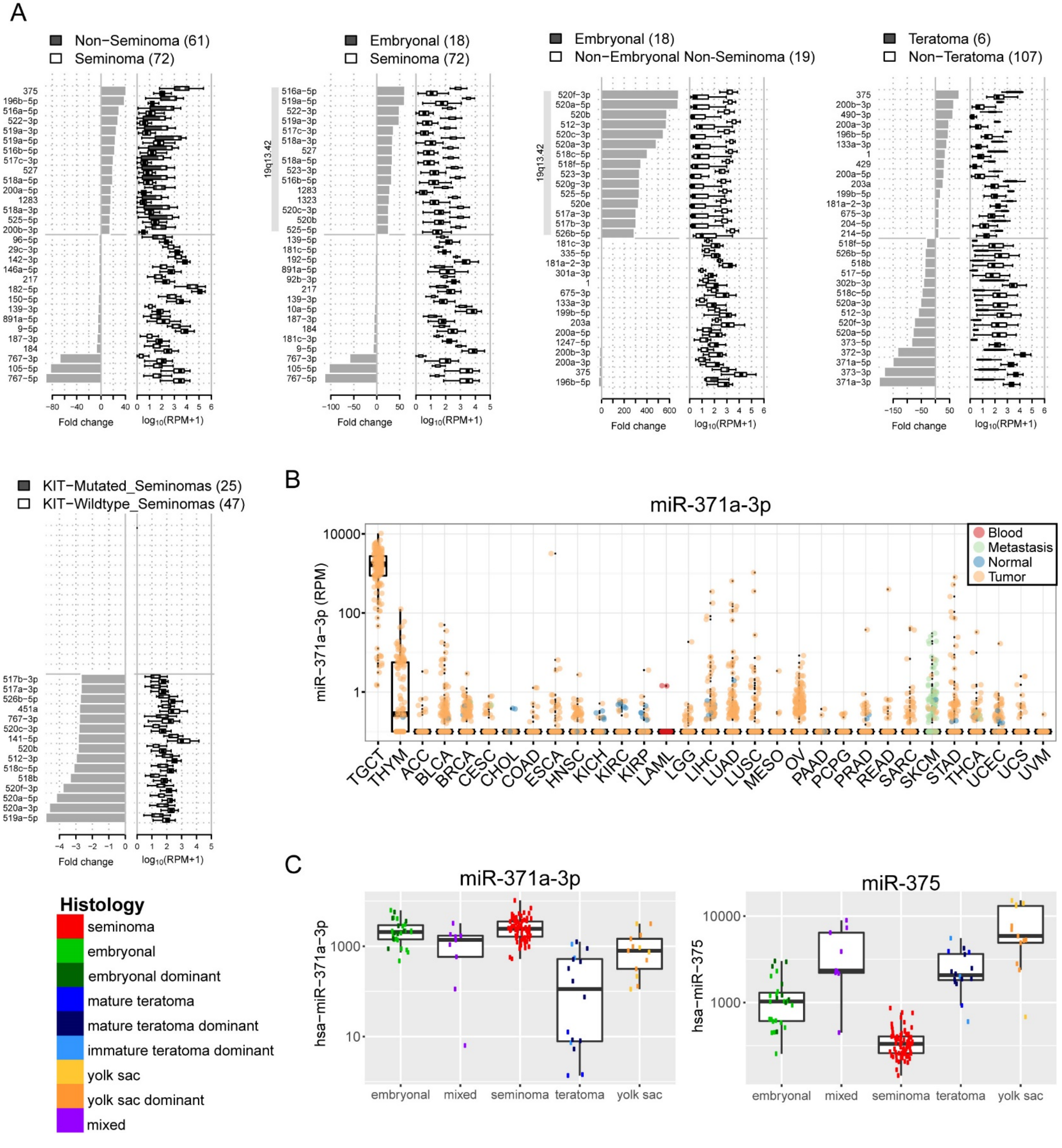

Figure S5. Related to Figure 1 and Tables S1, S4, and S5. miRNA analysis of 137 TGCTs. A) Differentially abundant miRNAs between histological types. Each panel has (left) a barplot of median-based fold change, and (right) boxplots showing distributions of normalized (RPM) abundance, with black vertical lines indicating medians. Up to 15 of the largest fold changes in each direction are shown. The numbers of samples in each group are in parentheses. Because miRNAs with higher abundance are likely more influential (Mullokandov 2012, Tay 2014), the graph shows only miRNAs that have a mean abundance of at least 50 RPM. **B)** TGCT-specific expression of miR-371a-3p in TCGA. RPM abundance of miR-371a-3p across TCGA tumor and normal samples is shown, sorted from left to right by decreasing median. **C)** Two additional discriminatory miRNAs (miR-371a-3p and miR-375) based on differential expression analysis are shown.

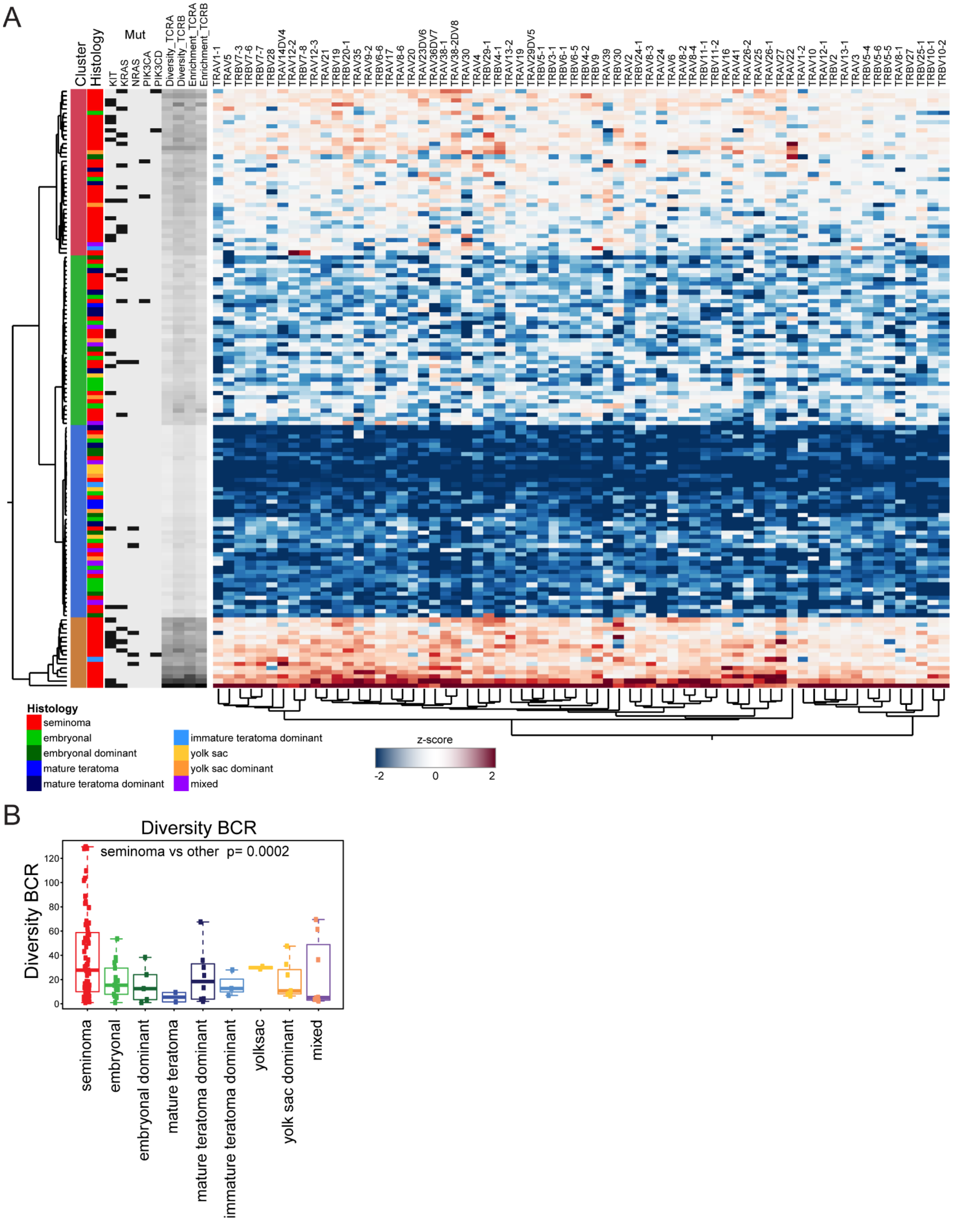

A
Cluster
Histology
Mut
KIT
KRAS
NRAS
PIK3CA
PIK3CD
Diversity_TCRA
Diversity_TCRB
Enrichment_TCRA
Enrichment_TCRB
TRAV1-1
TRAV5
TRBV7-3
TRBV7-6
TRBV7-7
TRBV28
TRAV14DV4
TRAV12-2
TRBV7-8
TRAV12-3
TRAV21
TRBV19
TRBV20-1
TRAV35
TRAV9-2
TRBV6-6
TRAV17
TRAV8-6
TRAV20
TRAV23DV6
TRAV36DV7
TRAV38-1
TRAV38-2DV8
TRAV30
TRAV4
TRBV29-1
TRBV4-1
TRAV13-2
TRAV19
TRAV29DV5
TRBV5-1
TRBV3-1
TRBV6-1
TRBV6-5
TRBV4-2
TRBV9
TRAV39
TRBV30
TRAV2
TRBV24-1
TRAV8-3
TRAV24
TRAV6
TRAV8-2
TRAV8-4
TRBV11-1
TRBV11-2
TRAV16
TRAV41
TRAV26-2
TRAV25
TRAV26-1
TRAV27
TRAV22
TRAV1-2
TRAV10
TRAV12-1
TRBV2
TRAV13-1
TRAV3
TRBV5-4
TRBV5-6
TRBV5-5
TRAV8-1
TRBV27
TRBV25-1
TRBV10-1
TRBV10-2
Histology
seminoma
embryonal
embryonal dominant
mature teratoma
mature teratoma dominant
immature teratoma dominant
yolk sac
yolk sac dominant
mixed
z-score
-2
0
2
B
Diversity BCR
seminoma vs other p= 0.0002
Diversity BCR
120
100
80
60
40
20
0
seminoma
embryonal
embryonal dominant
mature teratoma
mature teratoma dominant
immature teratoma dominant
yolksac
yolk sac dominant
mixed

Figure S6. Related to Figure 5. T-Cell and B-Cell sequence diversity across TGCTs and key molecular associations with immune infiltration in seminomas. A) The diversity, enrichment, and expression of T-cell receptor across testicular germ cell. **B)** B-cell receptor diversity across histological types testicular germs tumors.

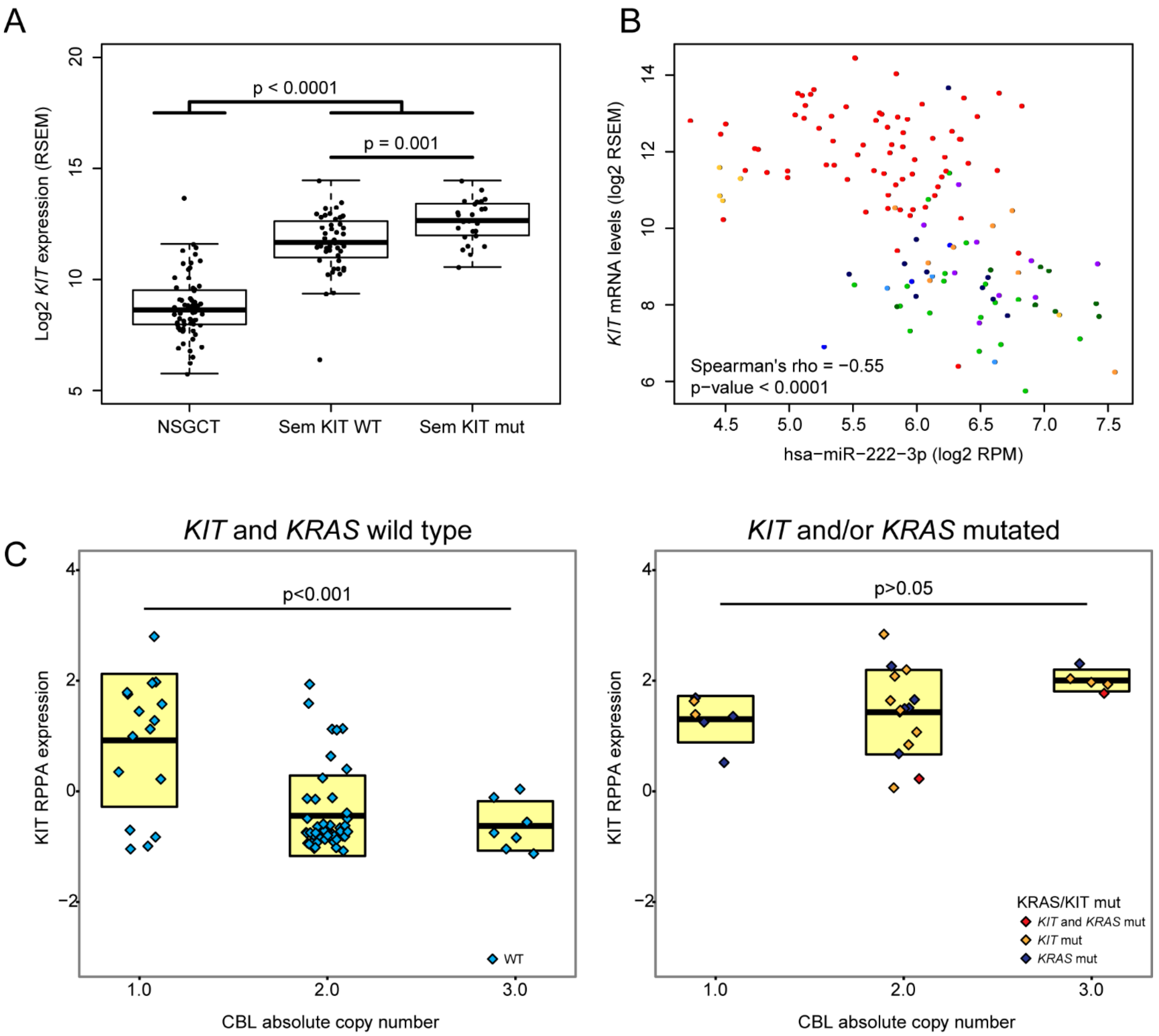

Figure S7. Related to Figure 6 and Table S1. Somatic alterations in *KIT* pathway genes. A) *KIT* gene expression is higher in in Sem and SEM with *KIT*-mutated. Nonparametric comparisons for each pair were done using the Wilcoxon Method to calculate the p values. **B)** Negative correlation between has-miR-222-3p and *KIT* mRNA expression. Spearman's rho and p-values were calculated. **C)** *CBL* copy number is negatively correlated with *KIT* RPPA expression in *KIT/KRAS* wild-type tumors (left), but not in *KIT/KRAS* mutated tumors (right). Tumors with mutations in *KIT*, *KRAS*, or both are indicated in yellow, dark blue, and red respectively and those wild-type (WT) for both genes are in light blue.

Table S1. Related to Table 1. Detailed Patient Characteristics.

Table S2. Related to Table 1. Patient Summary Characteristics.

	Histology of First or Only TGCT	
	Sem[a] (N=68)	NSGCT[b] (N=65)
Age at first or only diagnosis (median in years)	33	28
Race		
European descent (89%)	59	59
African descent (5%)	3	3
Asian descent (3%)	3	1
Unknown (4%)	3	2
Ethnicity		
Hispanic or Latino (9%)	6	6
Non-Hispanic (83%)	58	52
Unknown (8%)	4	7
Family history of any cancer (41%)	35	19
Family history of TGCT (11%)	7	7
Personal history of cryptorchidism (17%)	17	5
Detailed TGCT histology		
Seminoma[c]	68	-
Embryonal carcinoma[c]	-	18
Embryonal carcinoma dominant[d]	-	9
Immature teratoma dominant[d]	-	3
Mature teratoma[c]	-	3
Mature teratoma dominant[c]	-	10
Yolk sac[c]	-	5
Yolk sac dominant[d]	-	8
Mixed[e]	-	9
History of two primary TGCTs (7%)	3	6
Tumor Clinical Stage at diagnosis		
Stage I (70%)	52	41
Stage II-III (23%)	9	22
Unknown (7%)	7	2
Clinical outcomes		
Local recurrence (15%)	7	13
Distant metastasis (5%)	1	5

[a] 100% seminoma,

[b] Any histology except 100% seminoma

[c] 100% named histology

[d] >60% named histology

[e] No single dominant histology

Table S3. Related to Figure 2 and 3. Inferred integer copy number values by arm per tumor.

Table S4. Related to Figures 1, S1, and S5. Differentially abundant miRNAs by histology type.

Table S5. Related to Figures 1 and S5. Random forest classification out for miRNAs available to distinguish seminoma, embryonal carcinoma, and NSGCT.

Table S6. Related to Figures 1 and S1. Differentially expressed mRNAs by histology types.

Table S7. Related to Figures 1 and S1. Differentially expressed proteins by histology types.

Supplemental Experimental Methods

Sample Acquisition

Patients with testicular germ cell tumors were enrolled into the TCGA from 15 referral centers (Analytical Biological Services, Inc. (Indianapolis, IN, USA); Barretos Cancer Hospital (Barretos, Brazil); two contributing sites from Baylor (Houston, TX, USA); Cleveland Clinic (Cleveland, OH, USA); Erasmus Medical Center (Rotterdam, Netherlands); Gundersen Lutheran (La Crosse, WI, USA); International Genomics Consortium (Phoenix, AZ, USA); ProteoGenex (Culver City, CA, USA); Spectrum Health (Grand Rapids, MI, USA); University of Minnesota (Minneapolis, MN, USA); University of North Carolina (Chapel Hill, NC, USA); University of Pennsylvania (Philadelphia, PA, USA); University of Southern California (Los Angeles, CA, USA); University of Ulm (Ulm, Germany)) under IRB-approved protocols. Spermatocytic seminoma cases were excluded from this study. Primary tumor samples and matched germline control DNA (blood or blood components, including DNA extracted at the submitting site) were obtained from patients who had received no prior treatment for their disease (chemotherapy or radiotherapy; exception allowed for second primary tumor collection). Specimens were shipped overnight to the Biospecimen Core Resource (BCR) using a cryoport that maintained an average temperature of less than -180°C.

Cases were staged according to the American Joint Committee on Cancer (AJCC) staging system and, if the patient had received chemotherapy, the International Germ Cell Cancer Collaborative Group (IGCCG) staging. Pathology quality control was performed on each tumor specimen from either a frozen section slide prepared by the BCR or from a permanent section taken from a FFPE block immediately adjacent to the submitted frozen tumor specimen. Hematoxylin and eosin (H&E) stained sections from each sample were subjected to independent pathology review to confirm that the tumor specimen was histologically consistent the reported testicular germ cell histology. The percent rumor nuclei, percent necrosis, and other pathology annotations were also assessed. Tumor samples with ≥60% tumor nuclei and ≤20% necrosis were submitted for nucleic acid extraction.

TCGA Project Management has collected necessary human subjects documentation to ensure the project complies with 45-CFR-46 (the "Common Rule"). The program has obtained documentation from every contributing clinical site to verify that IRB approval has been obtained to participate in TCGA. Such documented approval may include one or more of the following:

- An IRB-approved protocol with Informed Consent specific to TCGA or a substantially similar program. In the latter case, if the protocol was not TCGA-specific, the clinical site PI provided a further finding from the IRB that the already-approved protocol is sufficient to participate in TCGA.
- A TCGA-specific IRB waiver has been granted.
- A TCGA-specific letter that the IRB considers one of the exemptions in 45-CFR-46 applicable. The two most common exemptions cited were are that the research fall under 46.102(f)(2) or 46.101(b)(4). Both exempt requirements for informed consent because the received data and material do not contain directly identifiable private information.
- A TCGA-specific letter that the IRB does not consider the use of these data and materials to be human subjects research. This was most common for collections in which the donors were deceased.

Sample Processing

DNA and RNA were extracted and quality was assessed at the central BCR. RNA and DNA were extracted from tumor using a modification of the DNA/RNA AllPrep kit (Qiagen). The flow-through from the Qiagen DNA column was processed using a *mir*Vana miRNA Isolation Kit (Ambion). This latter step generated RNA preparations that included RNA <200 nt suitable for miRNA analysis. DNA was extracted from blood using the QiaAmp DNA Blood Midi kit (Qiagen).

RNA samples were quantified by measuring Abs_{260} with a UV spectrophotometer and DNA quantified by PicoGreen assay. DNA specimens were resolved by 1% agarose gel electrophoresis to confirm high molecular weight fragments. A custom Sequenom SNP panel or the AmpFISTR Identifiler (Applied Biosystems) was utilized to verify that tumor DNA and germline DNA representing a case were derived from the same patient. Five hundred nanograms of each tumor and germline DNA were sent to Qiagen (Hilden, Germany) for REPLI-g whole genome amplification using a 100 µg reaction scale. RNA was analyzed via the RNA6000 Nano assay (Agilent) for determination of an RNA Integrity Number (RIN), and only analytes with a RIN≥7.0 were included in this study. Only cases yielding a minimum of 6.9 µg of tumor DNA, 5.15 µg RNA, and 4.9 µg of germline DNA were included in this study.

Sample Qualification

The BCR received tumor samples with germline controls from a total of 379 cases, of which 296 cases qualified. Of these qualified cases, 150 were sent for genomic analysis. Of the 83 that disqualified, 1 case was an unacceptable diagnosis, 4 cases had insufficient tumor nuclei (<60%), 2 cases had excessive necrosis (≥20%), 3 had both insufficient tumor nuclei and excessive necrosis. Another 3 had a genotypic mismatch between the tumor and germline samples, 39 had RNA integrity scores of <7.0, and the remaining had insufficient nucleic acid yields.

Twelve cases had sufficient residual tumor tissue following extraction of nucleic acids to allow for proteomics analysis. A 10 to 20 mg piece of snap-frozen tumor adjacent to the piece used for molecular sequencing and characterization was submitted to MD Anderson for reverse phase protein array (RPPA analysis).

Clinical Data
The clinical data collected included patient age, sex, race, ethnicity, height, weight, histologic diagnoses and percentage of each in the submitted tumor, tumor anatomic location and extent, history of undescended tesis and related correction information, history of hypospadias, pre- and post-treatment serum marker levels, clinical and pathologic AJCC staging, IGCCCG staging, history of prior cancers, synchronous cancers and subsequent cancers including distant metastasis or second primary cancers, date of death, and date of last contact.

Expert Pathology Committee Histologic Evaluation of Testicular Germ Cell Tumors
Hematoxylin and eosin (H&E) stained sections from each frozen tissue sample were subjected to independent pathology review by an expert pathology panel consisting of 4 genitourinary pathologists. Each pathologist independently reported the germ cell tumor component/s and the percentage of each component present in the frozen tissue block. The diagnosis was considered "consensus" when at least 3 pathologists agreed on the tumor components and their percentage (within a range of 10%) in the tissue block. In the small number of cases with non-consensus diagnosis (primarily because of less than ideal quality of the frozen tissue sections), the slides from the frozen tissue block were re-evaluated along with formalin-fixed, paraffin-embedded permanent tissue sections from the case to arrive at a final diagnosis.

Tumors were classified into histology groups. Tumors were classified as "pure" if they contained 100% of the histology on the EPC review and dominant if the sample had at least 60% of a given histology type. Samples with no dominant histology were classified as mixed.

Exome Mutation Analysis and Validation
WES library preparation
DNA samples were constructed into Illumina paired-end (PE) pre-capture libraries according to the manufacture's protocol (Illumina Multiplexing_SamplePrep_Guide_1005361_D) with modifications as described in the BCM-HGSC Illumina Barcoded Paired-End Capture Library Preparation protocol. Libraries were prepared using Beckman robotic workstations (Biomek NXp and FXp models). The complete protocol and oligonucleotide sequences are accessible from the HGSC website https://www.hgsc.bcm.edu/sites/default/files/documents/Illumina_Barcoded_Paired-End_Capture_Library_Preparation.pdf. Briefly, 1 μg DNA was sheared into fragments of 200–300 base pairs in a Covaris plate with E210 system (Covaris, Inc. Woburn, MA) followed by end-repair, A-tailing and ligation of Illumina multiplexing PE adaptors. Pre-capture Ligation Mediated-PCR (LM-PCR) was performed using the Library Amplification Readymix containing KAPA HiFi DNA Polymerase (Kapa Biosystems, Cat. No. KK2612). Universal primer IMUX-P1.0 and a pre-capture barcoded primer IBC were used in the PCR amplification. In total, a set of 12 such barcoded primers were used on these samples. Purification was performed with Agencourt AMPure XP beads after enzymatic reactions. Following the final XP beads purification, quantification and size distribution of the pre-capture LM-PCR product was determined using the LabChip GX electrophoresis system (PerkinElmer) and gel analysis using AlphaView SA v3.4 software.

Exome capture
For the hybridization step, four pre-capture libraries were pooled together (~250 ng/sample). These pooled libraries were then hybridized in solution to the HGSC VCRome 2.1 design (42Mb, NimbleGen) according to the manufacturer's protocol NimbleGen SeqCap EZ Exome Library SR User's Guide (Version 2.2) with minor revisions(Bainbridge et al., 2011). Human COT1 DNA and full-length Illumina adaptor-specific blocking oligonucleotides were added into the hybridization to block repetitive genomic sequences and the adaptor sequences. For post-capture LM-PCR amplification, either matching KAPA HiFi DNA Polymerase or Phusion PCR Supermix HiFi (2×) was used. After the final AMPure XP bead purification, quantity and size of the capture library was analyzed using the Agilent Bioanalyzer 2100 DNA Chip 7500. The efficiency of the capture was evaluated by performing a qPCR-based quality check on the four standard NimbleGen internal controls. Successful enrichment of the capture libraries was estimated to range from a 6 to 9 of ΔCt value over the non-enriched samples.

Sequencing
Library templates were prepared for sequencing using Illumina's cBot cluster generation system with TruSeq PE Cluster Generation Kits. Briefly, these libraries were denatured with sodium hydroxide and diluted to 6-9 pM in hybridization buffer in order to achieve a load density of ~800K clusters/mm. Each library pool was loaded in a single lane of a HiSeq flow cell, and each lane was spiked with 1% phiX control library for run quality control. The sample libraries then underwent bridge amplification to form clonal clusters, followed by hybridization with the sequencing primer. Sequencing runs were performed in paired-end mode using the Illumina HiSeq 2000 platform. Using the TruSeq SBS Kits, sequencing-by-synthesis reactions were extended for 101 cycles from each end, with an additional 7 cycles for the index read. Sequencing runs generated approximately 300-400 million successful reads on each lane of a flow cell, yielding 7-14 Gb per sample. With these sequencing yields, samples achieved an average of 96% of the targeted exome bases covered to a depth of 20X or greater.

WES data alignment
Initial sequence analysis was performed using the HGSC Mercury analysis pipeline(Reid et al., 2014). First, the primary analysis software on the instrument produces .bcl files that are transferred off-instrument into the HGSC analysis infrastructure by the HiSeq Real-time Analysis module. Once the run is complete and all .bcl files are transferred, Mercury runs the vendor's primary analysis

software (CASAVA), which demultiplexes pooled samples and generates sequence reads and base-call confidence values (qualities). The next step is the mapping of reads to the GRCh37 Human reference genome (http://www.ncbi.nlm.nih.gov/projects/genome/assembly/grc/human/) using the Burrows- Wheeler aligner (BWA(Li and Durbin, 2009), http://bio-bwa.sourceforge.net/) and producing a BAM (binary alignment/map) file(Li et al., 2009). The third step involves quality recalibration (using GATK(DePristo et al., 2011), http://www.broadinstitute.org/gatk/), and where necessary the merging of separate sequence event BAMs into a single sample-level BAM. BAM sorting, duplicate read marking, and realignment to improve insertion and deletion (Indel) discovery all occur at this step.

Mutation calling

Mutation calling by multiple analysis centers was carried out, essentially as previously described (The Cancer Genome Atlas Research Network, 2013). Mutations were called using tumor and matched normal BAM files by the BCM HGSC, Broad Institute, University of California, Santa Cruz (UCSC), and British Colombia Genome Sequencing Center.
Strelka (Saunders et al., 2012)(v1.0.6) was used to identify somatic single nucleotide variants, and short insertions and deletions from the TCGA TGCT exome dataset. All parameters were set to defaults, with the exception of "isSkipDepthFilters", which was set to 1 in order to skip depth filtration given the higher coverage in exome datasets. 156 pairs of libraries were analyzed. When a blood sample was available, it served as the matched normal specimen; otherwise, the matched normal tissue was used. The variants were subsequently annotated using SnpEff (Cingolani et al., 2012)and the COSMIC (v61)(Forbes et al., 2009) and dbSNP (v137) (Smigielski et al., 2000)databases.

Single nucleotide somatic mutations were identified by RADIA (RNA AND DNA Integrated Analysis), a method that combines the patient matched normal and tumor DNA whole exome sequencing (DNA-WES) with the tumor RNA sequencing (RNA-Seq) for somatic mutation detection (software available at: https://github.com/aradenbaugh/radia/)(Radenbaugh et al., 2014). The inclusion of the RNA-Seq data in RADIA increases the power to detect somatic mutations, especially at low DNA allelic frequencies. By integrating the DNA and RNA, mutations that would be missed by traditional mutation calling algorithms that only examine the DNA can be rescued back. RADIA classifies somatic mutations into 3 categories depending on the read support from the DNA and RNA: 1) DNA calls – mutations that had high support in the DNA, 2) RNA Confirmation calls – mutations that had high support in both the DNA and RNA, 3) RNA Rescue calls – mutations that had high support in the RNA and weak support in the DNA. Here RADIA identified 6,894 DNA mutations, 908 RNA Confirmation mutations, and 366 RNA Rescue mutations.

Significantly mutated gene and mutational signature analysis

The statistical significance of mutation frequency in each gene was determined using the MutSigCV algorithm (v1.4)(Lawrence et al., 2013). A p value of < 0.05 and a q value (false discovery rate) of < 0.1 were considered statistically significant. Mutation signature analysis was performed as previously described(Wang et al., 2015).

Mutation validation using NimbleGen capture array

Twenty-five frequently altered genes and five SNPs in functionally related genes were selected for mutation validation. A custom NimbleGen array (target size: ~200kb) was designed for mutation validation. All the samples including both tumors their matched control DNAs were sequenced. The basic Illumina library preparation protocol and the regents used were identical to that of WES except that for validation, a set of 96 molecular barcodes were used to allow high density multiplexing. The capture enrichment protocol was also same as described earlier for WES, with the following modifications: for capture, six pools of 43-48 libraries/pool were prepared and hybridized in solution to the custom capture design. Overnight hybridization at 56^0C was carried out instead of the 3-day hybridization at 47^0C as done for WES. Post enrichment steps were also similar: six enriched pools were pooled into three groups (86, 92, 92 libraries/pool) and sequenced on three lanes of HiSeq 2500. Except one control sample (TCGA-2G-AAKM-10A-01D-A433-10) had a library failure, all the samples were successfully sequenced. On an average, 298 Mb of uniquely mapped sequence data were generated across the target region and the targeted bases were covered to 599×.

Genes and sites selected for validation study.

**Gene**	**Selection criteria**	**Design**
KIT	Significantly mutated gene by MutSigCV	capture array#
KRAS	Significantly mutated gene by MutSigCV	capture array
NRAS	Significantly mutated gene by MutSigCV	capture array
PIK3CD	KIT/RAS/PI3K pathway, Recurrently mutated	capture array
PIK3CA	KIT/RAS/PI3K pathway, Recurrently mutated	capture array
PIK3CB	KIT/RAS/PI3K pathway, Recurrently mutated	capture array
PIK3C2G	KIT/RAS/PI3K pathway, Recurrently mutated	capture array
PIK3IP1	KIT/RAS/PI3K pathway, Recurrently mutated	capture array

SOS1	KIT/RAS/PI3K pathway, Recurrently mutated	capture array
NF1	Regulator of KIT/RAS/PI3K pathway, Recurrently mutated	capture array
PIK3C2A	KIT/RAS/PI3K pathway	capture array
PIK3C2B	KIT/RAS/PI3K pathway	capture array
PIK3R1	KIT/RAS/PI3K pathway	capture array
PIK3R5	KIT/RAS/PI3K pathway	capture array
BRAF	KIT/RAS/PI3K pathway	capture array
AKT3	KIT/RAS/PI3K pathway	capture array
MTOR	KIT/RAS/PI3K pathway	capture array
CBL	Regulator of KIT/RAS/PI3K pathway	capture array
JARID2	Frequently altered, with mutation, focal deletion and fusion, functionally important	capture array
CREBBP	Recurrently mutated and functionally important	capture array
SRCAP	Recurrently mutated, CREBBP activator	capture array
BIRC6	Recurrently mutated and functionally important	capture array
NUDC	Fusion partner of JARID2	capture array
rs995030	KITLG SNP	capture array
rs1508595	KITLG SNP	capture array
rs4590952	KITLG SNP	capture array
rs4624820	SPRY4 SNP	capture array
rs60180747	MAP2K1 SNP	capture array
CYTB	Recurrent mutation, mitochondrial gene, a mediator of FAS-induced apoptosis	LR-PCR*
RNR2	Recurrent mutation, mitochondrial RNA gene, suppress apoptosis	LR-PCR

#Capture array, NimbleGen targeted capture array; *LR-PCR: long-range PCR.

Mutation validation using LR-PCR

Mutations in two mitochondrial genes (CYTB and RNR2) were validated using long-range PCR (LR-PCR) approach. The mitochondrial DNA (mtDNA) were isolated from the tumor and matched control of 17 related TGCT cases, the LR-PCR, Illumina library construction and sequencing was performed using the same protocol as previously described(Davis et al., 2014). Briefly, the mtDNA products were successfully obtained from all 34 samples. A total of 500 ng final mtDNA LR-PCR product was used for Illumina paired-end library construction. All libraries were equally pooled and sequenced on one lane of HiSeq 2500. The sequencing reads were mapped to the mitochondrial reference genome (NC_012920.1 Mito Reference) using BWA(Li and Durbin, 2009), producing a BAM file(Li et al., 2009). The workflow of sequencing data processing and mutation calling was identical to that of WES.

Validation data analysis

A mutation is valid somatic if it was detected in the tumor sample but not in its matched control sample and passed all the quality filters as shown in the figure.

Mutation filtering and generation of final somatic MAF

To prepare the final somatic MAF for downstream integrative analysis, a stepwise filtering approach was applied to the multi-center MAF. First, the quality filters we applied and only those meeting the following criteria were selected: 1) the total read coverage in tumor and its matched control sample ≥ 5; 2) the variant read coverage in tumor ≥ 5 and the variant allele fraction (VAF) in tumor ≥ 0.05; 3) the ratio of VAF in normal and its tumor ≤ 0.15. Second, the germline filters were applied and any variants observed in the germline MAF file of the cohort were removed. Third, common SNPs were filtered out using dbSNP139 (MAF$\leq$0.005). Then, all mutations left were visualized in Integrative Genomics Viewer (IGV, https://www.broadinstitute.org/igv/) and the mapping quality and uniqueness of the supporting reads was assessed using BLAT (https://genome.ucsc.edu/FAQ/FAQblat.html). The ones with ambiguous mapping and poor mapping quality were removed from the list. After that, the non-coding calls (at 3'UTR, 5'UTR, 3'Flank, 5'Flank, RNA, Intron, IGR) were removed. Finally, the validation data were added, the invalid calls, valid germline calls, and the single-center calls without validation data were removed. The valid mutations and cancer hotspot mutations are retained.

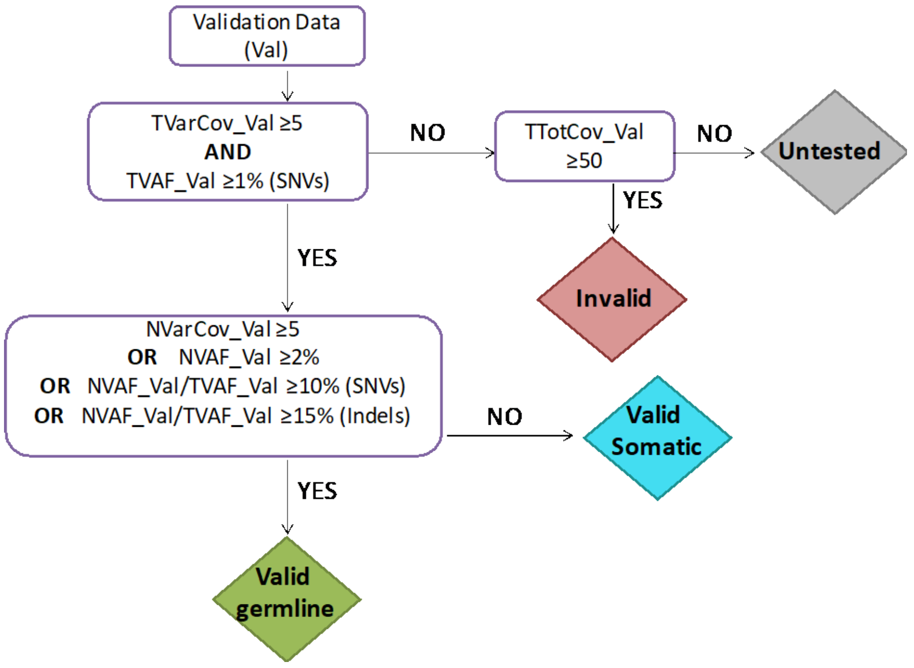

The workflow for validation analysis. Val, validation data; TVarCov, the tumor variant coverage; TVAF, the tumor variant allelic fraction; TTotCov, the tumor total coverage; NVarCov, the control variant coverage, NVAF, the control variant allelic fraction; NVAF_Val/TVAF_Val, the ratio of the variant allelic fraction in the matched control sample to that of the tumor sample.

Pathway Analysis

All sentinel loci established from eleven published germ-line GWAS of individuals with history of TGCT were evaluated. All genes within r2>0.8 linkage disequilibrium (LD) of the sentinel locus were included in analysis. Where more than one gene lay within the LD block and one gene contained a highly suggestive coding change in close LD with the sentinel risk variant, analysis of that gene was favored (e.g. *PMF1*). PubMed (http://www.ncbi.nih.gov/pubmed/), Online Mendelian Inheritance in Man (http://omim.org), and the Mouse Genome Informatics (http://www.informatics.jax.org/) databases were systematically searched using the gene name as primary search term to elicit primary references (April 2016). Where multiple historic names for the same gene were identified (e.g. *ZFP42/REX1*) each name was searched independently. Genes were assigned to pathways as stated by functional studies in the primary literature. All implicated pathways were included, including where multiple pathways were implicated.

Risk Alleles

Risk allele genotypes were determined by imputation for 36 SNPs in 128 cases in the cohort, and were assigned a risk burden value of 0 (homozygous for risk allele), 1 (heterozygous), or 2 (homozygous for non-risk allele).

Viral detection

The microbial database (v10) used for virus mapping was built by the CAMERA project (The Community Cyberinfrastructure for Advanced Marine Microbial Ecology Research and Analysis, http://camera.calit2.net) and was downloaded from its ftp site (ftp://portal.camera.calit2.net/ftp-links/cam_datasets/blastdb/). All unmapped reads (both paired and unpaired) were extracted from the full RNA-seq bam file. PCR or optical duplicates as well as anything that does not meet QC standards were removed and the remaining unmapped reads were converted to FASTA format. The unmapped reads were blasted against the microbial database to look for paired reads in which one read mapped to the human genome and the other mapped to the virus genome. Only unmapped reads in which had a significant blast hit to the virus genome and have the pair read mapped to the human genome were included for further analysis. The NDC (normalized depth coverage) was then calculated. NDC is the depth (total base pair) coverage normalized by the percent coverage, it is a combined scoring metric that takes into account the total number of mapped base pair to a given virus as well as the distribution of those mapped base pairs across that virus.

Filtering, annotation, and analysis of germline variants

The germline variants were filtered against the Phase-3 1000 Genome Project (http://phase3browser.1000genomes.org/Homo_sapiens/Info/Index), the NHLBI GO Exome Sequencing Project (ESP6500, http://evs.gs.washington.edu/EVS/), and the ExAc (the Exome Aggregation Consortium, http://exac.broadinstitute.org) and the variant with a population minor allele frequency greater than 0.5% were removed. The nonsynonymous variants including nonsense, missense, frame-shift variants and variants at splicing site were then selected for further analysis and the intronic mutations, mutations

at 3’ or 5’ UTR or UTR flanking regions, silent mutations, small in-frame insertions and deletions were excluded. To further evaluate the probability of a missense mutation being functionally deleterious, dbNSFP (v3.0) (Liu et al., 2016) was applied to add prediction scores for all missense mutations from twelve commonly used functional prediction algorithms: Polyphen-2 (Adzhubei et al., 2013), SIFT (Kumar et al., 2009), MutationTaster (Schwarz et al., 2014), Mutation Assessor (Reva et al., 2011), LRT (Chun and Fay, 2009), FATHMM-MKL (Shihab et al., 2015) and DANN (Quang et al., 2015), PROVEAN(Choi et al., 2012), WEST3 (Carter et al., 2013), CADD (Kircher et al., 2014), GERP++(Davydov et al., 2010), MetaSVM and MetaLR (Dong et al., 2015). A missense mutation that was called as “deleterious” or “damaging” by five or more algorithms were defined as a “deleterious” mutation. The genes with truncating or deleterious variants in two or more patients with documented 2nd primary tumors (n=9) were selected and the gene mutation frequencies were compared to patients without documented 2nd primary tumors (n=124) on the freeze set and the p-values were calculated using two-tailed Fisher’s Exact test.

Copy number analysis

SNP-based copy number analysis

DNA from each tumor or germline sample was hybridized to Affymetrix SNP 6.0 arrays using protocols at the Genome Analysis Platform of the Broad Institute as previously described(McCarroll et al., 2008). From raw .CEL files, Birdseed was used to infer a preliminary copy-number at each probe locus(Korn et al., 2008). For each tumor, genome-wide copy number estimates were refined using tangent normalization, in which tumor signal intensities are divided by signal intensities from the linear combination of all normal samples that are most similar to the tumor(Network, 2011) (and Tabak B. and Beroukhim R. Manuscript in preparation). This linear combination of normal samples tends to match the noise profile of the tumor better than any set of individual normal samples, thereby reducing the contribution of noise to the final copy-number profile. Individual copy-number estimates then underwent segmentation using Circular Binary Segmentation(Olshen et al., 2004). As part of this process of copy-number assessment and segmentation, regions corresponding to germline copy-number alterations were removed by applying filters generated from either the TCGA germline samples from the ovarian cancer analysis or from samples from this collection. Segmented copy number profiles for tumor and matched control DNAs were analyzed using Ziggurat Deconstruction, an algorithm that parsimoniously assigns a length and amplitude to the set of inferred copy-number changes underlying each segmented copy number profile(Mermel et al., 2011). Allelic copy number, whole genome doubling, subclonality, and purity and ploidy estimates were calculated using the ABSOLUTE algorithm(Carter et al., 2012). For samples with ABSOLUTE corrected copy number, CBS-derived segmented copy number values were re-centered using the *In Silico* Admixture Removal (ISAR) procedure(Zack et al., 2013). Significant focal copy number alterations across all tumors and within each histologic subtype were identified from ISAR-corrected segmented data using GISTIC 2.0.22(Mermel et al., 2011). For copy number based clustering, tumors were clustered based on total integer arm-level copy number as derived from the ABSOLUTE algorithm, normalized over four copies for tumors estimated to have been whole genome doubled once, and eight copies for tumors estimated to have been whole genome doubled twice. Clustering was done in R based on Manhattan distance using Ward's method. Timing of arm-level copy number alterations was inferred based on frequency of the event and level of aneuploidy in tumors containing the event relative to other events(Frigyesi et al., 2004). Assuming that level of aneuploidy increases overall during tumor progression, events associated with low levels of aneuploidy were predicted to occur early and events associated with high aneuploidy were predicted to occur late.

Inference of isochromosome 12p status

For samples with ABSOLUTE corrected copy number (as described above), integer segmental copy numbers were designated to allele a1 or a2 (minor or major alleles respectively). The modal copy number was recorded across each chromosome arm for both a1 and a2 (see “Before Inference” below). If the a1 (minor) allele copy number of chromosome arm 12p was equal to either the minor or major allele copy number of 12q, the specified 12q allele was inferred to correspond with a1; i.e., from the same homologous chromosome as the 12p a1 (minor) allele. If the difference between the a2 copy number of 12p and the a2 copy number of 12q was greater or equal to 2, the tumor was considered consistent with harboring an isochromosome 12p.

Tumor	TCGA-2G-AAFJ-01		
	chr 12 arm	modal a1	modal a2
Before Inference	p	2	5
	q	1	2

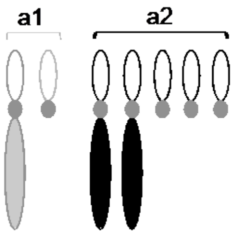

After Inference	p	2	5
	q	2	1

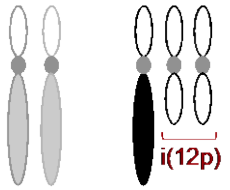

Methylation Analysis

Data processing

We performed bisulfite conversion on 1 µg of genomic DNA from 157 samples (including 150 primary, five secondary TGCT tumors and two technical replicates) using the EZ-96 DNA Methylation Kit (Zymo Research, Irvine, CA) according to the manufacturer's instructions. We assessed the amount of bisulfite converted DNA and completeness of bisulfite conversion using a panel of MethyLight-based quality control (QC) reactions as previously described(Campan et al., 2009). All bisulfite-converted DNA samples were then subject to repair using the Illumina Restoration Solution. The entire amount of each repaired sample entered the Infinium DNA methylation assay pipeline. Bisulfite-converted DNAs were whole genome amplified (WGA) and enzymatically-fragmented prior to hybridization to the Illumina Infinium HumanMethylation450 Beadchip array. BeadArrays were scanned using the Illumina iScan technology to produce IDAT files. Raw IDAT files for each sample were processed with the R/Bioconductor package *methylumi*.

Level 1 data contain raw IDAT files (two per sample) as produced by the iScan system and as mapped by the SDRF. These IDAT files can be directly processed by the R/Bioconductor package *methylumi*. Level 2 data contain background-corrected methylated (M) and unmethylated (U) summary intensities as extracted by the R/Bioconductor package *methylumi*. Non-detection probabilities (*P* values) were computed as the minimum of the two values (one per allele) for the empirical cumulative density function of the negative control probes in the appropriate color channel. Background correction was performed via normal-exponential deconvolution, as previously described(Triche et al., 2013). Multiple-batch archives have the intensities in each of the two channels multiplicatively scaled to match a reference sample (sample with R/G ratio of the normalization control probes closest to 1.0). Level 3 data contain β-value calculations with annotations for HGNC gene symbol, chromosome (UCSC hg19, Feb 2009), and genomic coordinate (UCSC hg19, Feb 2009) for each targeted CpG/CpH site on the array. Probes having a common SNP (Minor Allele Frequency > 0.01, per dbSNP build 135 via the UCSC snp135common track) within 10bp of the interrogated CpG site or having a 15bp from the interrogated CpG site which overlaps with a repetitive element (as defined by RepeatMasker and Tandem Repeat Finder Masks contained in the *BSgenome.Hsapiens.UCSC.hg19* R package) were masked as "NA" across all samples, and probes with a non-detection probability (*P* value) greater than 0.05 in a given sample were masked as "NA" on that array. Probes that were mapped to multiple sites on hg19 were annotated as "NA" for chromosome and 0 for CpG/CpH coordinate.

Unsupervised clustering

We downloaded Level 3 DNA methylation data from the TCGA data portal. For each CpG probe, we calculated the standard deviation (SD) of beta values across the 137 tumor samples in the final data freeze. Autosomal CpG probes (with probe IDs starting with 'cg') with an SD of greater than 0.26 (n=9,614) were used for clustering. The R function *hclust* was used for hierarchical clustering of these tumors with Ward's method, and the resulting dendrogram was cut at different levels with the R function *cutree* to evaluate cluster stability and biological relevance. When the tree is cut at k=2, seminomas and non-seminomas formed two major clusters. As illustrated in Figure S1, seminomas with different degrees of lymphocytic infiltration/contamination split from the main seminoma cluster, and non-seminomas further divided into two clusters, one with mixed components and low CpH methylation, and another primarily with embryonal components and relatively high CpH methylation at k=5. Therefore, we renamed the DNA methylation clusters to Sem-HC (seminomas with high contamination, Cluster 2), Sem-MC (seminomas with moderate contamination, Cluster 1), Sem-LC (seminomas with low contamination, Cluster 5), Mixed (Cluster 4) and CpH-high (Cluster 3). These names suggest group behavior and a sample in the CpH-high group could have low CpH methylation level.

Estimate of Lymphocyte Percentage and Subtraction of Lymphocyte DNA Contamination

We downloaded sorted blood dataset as previously published (GSE49618), and calculated mean DNA methylation beta values for lymphocytes (CD4 Tell, CD8 T cell, CD19 B cell) for each CpG locus. We correlated the DNA methylation levels observed in seminomas with different normal cell types generated throughout TCGA and in house and identified lymphocytes to be of the highest correlation. We identified 719 CpGs with high methylation level in lymphocytes (mean beta value >0.7) and low in non-seminomas (mean beta value <0.3) (Group 1 in Figure S1B). These probes represent a lymphocyte methylation signatures on an unmethylated testicular germ cell tumor background; therefore, we calculated the mode of kernel-smoothed DNA methylation distribution at these sites for each sample *j* as a surrogate for lymphocyte fraction for that sample, denoted as $\pi_{lym,j}$. 492 probes with low DNA methylation level in lymphocytes (mean beta value <0.3) and high DNA methylation level in non-seminomas (mean beta value >0.7) (Group 2 probes in Figure S1B) were used as a control, as seminomas were indistinguishable from lymphocytes at these loci and therefore lymphocytic infiltration would not influence the DNA methylation readouts in seminomas, but will dilute the high-level DNA methylation in non-seminomas. In contrast, no myeloid-specific DNA methylation fingerprint (Group 3 probes in Figure S1B) was observed. Pearson correlation coefficient between the calculated lymphocyte estimates with ABSOLUTE-derived tumor purity using 131 samples with estimates from both platforms was calculated with R and was determined to be -0.94.

Using a linear model, we can derive the corrected methylation (beta value) level for tumor sample *j* at locus *i*, denoted as $\beta'_{tum,i,j}$ using the following equation:

$$\beta'_{tum,i,j} = \frac{\beta_{tum,i,j} - \beta_{lym,i,j} * \pi_{lym,j}}{1 - \pi_{lym,j}}$$

Imprinted Loci

We downloaded the list of imprinted loci from a previously published study (Court et al., 2014), along with reference samples used in this study (GSE52578).

miRNA Sequencing Analysis

microRNA libraries and sequencing

We generated microRNA sequence (miRNA-seq) data for 133 TGCT tumor samples using methods described previously except that 1ug of total RNA (at 250ng/uL) was used as input instead of messenger RNA-depleted RNA (Chu et al., 2015). Briefly, reads were aligned to the GRCh37/hg19 reference human genome using BWA-MEM, and read count abundance was annotated with miRBase v16 stemloops and mature strands. While the read counts included only exact-match read alignments, .bam files at CGHub (cghub.ucsc.edu) (Wilks et al., 2014) include all sequence reads. We used miRBase v20 to assign 5p and 3p mature strand (miR) names to MIMAT accession IDs.

Unsupervised clustering of miRNA mature strands

To identify subtypes within the TGCT cohort we used hierarchical clustering with pheatmap v1.0.2 in R. The input was a reads-per-million (RPM) data matrix for the 303 (top 25%) miRBase v16 5p or 3p mature strands that had the largest variances across the cohort. We transformed each row of the matrix by $\log_{10}(RPM + 1)$, then used pheatmap to scale the rows. We used Ward.D2 for the clustering method with Pearson correlation and Euclidean as the distance measures for clustering the columns and rows respectively (Figure S3A).

Differentially abundant microRNAs

We identified miRs that were differentially abundant using unpaired two-class SAM analyses (samr v2.0) with an RPM input matrix and an FDR threshold of 0.05 (Li and Tibshirani, 2013). We compared pure seminoma (n=72) to samples with no seminoma component (n=61), pure seminoma (n=72) to pure embryonal (n=18), pure embryonal (n=18) to samples with no embryonal and no seminoma component (n=19), samples that only have a mature or immature teratoma component (n=6) to samples that have no mature or immature teratoma component (n=107), and pure seminomas with a KIT mutation (n=25) to pure seminomas without a KIT mutation (n=47) (Figure S3B, Table S4).

Random Forest

We ranked miRNAs from most important to least important for classifying samples into 3 groups: pure seminoms (n=72), pure or dominant embryonal (n=27), and pure or dominant yolk sac and teratoma (mature/immature) (n=29). The 9 samples that had no dominant histology were excluded from this analysis. To do this, we used a random forest classification software from Salford Systems, Inc and created 10,000 trees, each using 3 miRNAs as predictors (Figure S3C, Table S5).

mRNA Sequencing Analysis

RNA library construction, sequencing, and analysis

One µg of total RNA was converted to mRNA libraries using the lllumina mRNA TruSeq kit (RS-122-2001 or RS-122-2002) following the manufacturer's directions. Libraries were sequenced 48x7x48bp on the Illumina HiSeq 2000 as previously described (TCGA, 2012). FASTQ files were generated by CASAVA. RNA reads were aligned to the hg19 genome assembly using MapSplice 0.7.4 (Wang et al., 2010). Gene expression was quantified for the transcript models corresponding to the TCGA GAF2.1 (http://tcga-data.nci.nih.gov/docs/GAF/GAF.hg19.June2011.bundle/outputs/TCGA.hg19.June2011.gaf), using RSEM (Li and Dewey, 2011) and normalized within-sample to a fixed upper quartile. For further details on this processing, refer to Description file at the DCC data portal under the V2_MapSpliceRSEM workflow (https://tcga-data.nci.nih.gov/tcgafiles/ftp_auth/distro_ftpusers/anonymous/tumor/tgct/cgcc/unc.edu/illuminahiseq_rnaseqv2/rnaseqv2/unc.edu_TGCT.IlluminaHiSeq_RNASeqV2.mage-tab.1.0.0/DESCRIPTION.txt) or our alignment pipeline summary at CGHUB (https://cghub.ucsc.edu/docs/tcga/UNC_mRNAseq_summary.pdf).

Quantification of genes, transcripts, exons and junctions can be found at the TCGA Data Portal (https://tcga-data.nci.nih.gov/tcga/).

Expression data processing

Gene expression data was median centered and log2 transformed using the cluster 3.0 software(de Hoon et al., 2004). Missing data was imputed using Euclidean distance to find the nearest neighbor and calculate the missing value using the impute.knn R function(Hastie et al., 1999).

Subtype analysis

Seminoma subtypes were identified using consensus cluster plus(Wilkerson and Hayes, 2010). Gene selection criteria included genes with a standard deviation and overall log2 expression level greater than or equal to 1.5; yielding 1,504 genes for consensus clustering. Average silhouette widths were calculated using the R package 'cluster'(Rousseeuw, 1987). Principle components analysis was conducted and visualized in Matlab using the source code available(Hollern and Andrechek, 2014).

Pathway and Immune Module Analysis

Gene expression analysis of pathway and immune signatures was conducted by median centering samples to the median expression of each signature in R(Fan et al., 2011). Within each histology, samples were clustered based on expression of the immune signatures using the centroid linkage setting in Cluster 3.0(de Hoon et al., 2004). Gene signatures were compiled from individual manuscripts (Bindea et al., 2013; Fan et al., 2011; Gatza et al., 2010; Ghassabeh et al., 2006; Iglesia et al., 2014; Rody et al., 2009)and the broad institute's molecular signature database.

Testes Specific Antigens
The list of antigens used for analyses was derived from the CTdatabase(Almeida et al., 2009).

T-Cell Receptor and B-Cell Receptor Diversity
T-cell receptor diversity was calculated using MixCr(Bolotin et al., 2015; Bolotin et al., 2013). MiXCR is available at http://mixcr.milaboratory.com/ and https://github.com/milaboratory/mixcr/.

B-cell receptor diversity was calculated using V'DJer(Mose et al., 2016). V'DJer is implemented in C/C ++, free for academic use and can be downloaded from Github: https://github.com/mozack/vdjer.

Box and X-Y Correlation Plots
Correlation and box plots were built in R Studio. The R value was calculated using pearson correlation and the cor.test function in R.

RPPA Analysis

Protein extraction and data processing
Protein was extracted using RPPA lysis buffer (1% Triton X-100, 50 mmol/L Hepes (pH 7.4), 150 mmol/L NaCl, 1.5 mmol/L MgCl2, 1 mmol/L EGTA, 100 mmol/L NaF, 10 mmol/L NaPPi, 10% glycerol, 1 mmol/L phenylmethylsulfonyl fluoride, 1 mmol/L Na3VO4, and aprotinin 10 ug/mL) from human tumors and RPPA was performed as described previously (Coombes et al., 2009; Hennessy et al., 2007; Hu et al., 2007; Liang et al., 2007; Tibes et al., 2006). Lysis buffer was used to lyse frozen tumors by Precellys homogenization. Tumor lysates were adjusted to 1 µg/µL concentration as assessed by bicinchoninic acid assay (BCA) and boiled with 1% SDS. Tumor lysates were manually serial diluted in two-fold of 5 dilutions with lysis buffer. An Aushon Biosystems 2470 arrayer (Burlington, MA) printed 1,056 samples on nitrocellulose-coated slides (Grace Bio-Labs). Slides were probed with 218 validated primary antibodies followed by corresponding secondary antibodies (Goat anti-Rabbit IgG, Goat anti-Mouse IgG or Rabbit anti-Goat IgG). Signal was captured using a DakoCytomation-catalyzed system and DAB colorimetric reaction. Slides were scanned in a CanoScan 9000F. Spot intensities were analyzed and quantified using Array-Pro Analyzer (Media Cybernetics Washington DC) to generate spot signal intensities (Level 1 data). The software SuperCurveGUI (Coombes et al., 2009; Hu et al., 2007), available at http://bioinformatics.mdanderson.org/Software/supercurve/, was used to estimate the EC50 values of the proteins in each dilution series (in log2 scale). Briefly, a fitted curve ("supercurve") was plotted with the signal intensities on the Y-axis and the relative log2 concentration of each protein on the X-axis using the non-parametric, monotone increasing B-spline model(Tibes et al., 2006). During the process, the raw spot intensity data were adjusted to correct spatial bias before model fitting. A QC metric (Coombes et al., 2009) was returned for each slide to help determine the quality of the slide: if the score is less than 0.8 on a 0-1 scale, the slide was dropped. In most cases, the staining was repeated to obtain a high quality score. If more than one slide was stained for an antibody, the slide with the highest QC score was used for analysis (Level 2 data). Protein measurements were corrected for loading as described [3,5,6] using median centering across antibodies (level 3 data). In total, 218 antibodies and 104 Testicular Germ Cell Tumor (TGCT) samples were used for the analysis, including 51 seminoma, 20 embryonal carcinoma, 19 non-seminoma (mixed), 6 yolk sac tumors, 6 mature teratomas, and 2 immature teratomas. Final selection of antibodies was also driven by the availability of high quality antibodies that consistently pass a strict validation process as previously described(Hennessy et al., 2010). These antibodies are assessed for specificity, quantification and sensitivity (dynamic range) in their application for protein extracts from cultured cells or tumor tissue. Antibodies are labeled as validated and use with caution based on degree of validation by criteria previously described (Hennessy et al., 2010).

RPPA arrays were quantitated and processed (including normalization and load controlling) as described previously, using MicroVigene (VigeneTech, Inc., Carlisle, MA) and the R package SuperCurve (version-1.3), available at http://bioinformatics.mdanderson.org/OOMPA (Hu et al., 2007; Tibes et al., 2006). Raw data (level 1), SuperCurve nonparameteric model fitting on a single array (level 2), and loading corrected data (level 3) were deposited at the DCC.

Data normalization
Data was normalized by median centering across all the antibodies for each sample to correct for sample loading differences.

RPPA subtypes
RPPA subtypes were derived using consensus clustering(Monti et al., 2003). Pearson correlation was used as distance metric and Ward was used as a linkage algorithm in the clustering analysis. A total of 104 samples and 218 antibodies were used in the analysis.

Pathway analysis

To illustrate the role of cell signaling network in TGCT, we calculated twelve pathway scores based on a previously described method (Akbani et al., 2014).

Cross Platform analysis

Neoantigen predictions

TCGA Testicular Germ Cell Tumor samples with available mRNA-sequencing data, exome-sequencing data, and tumor-specific mutation annotation data were used as inputs for the neoantigen prediction pipeline. The Neoantigen prediction bioinformatics pipeline was similar to that developed by Rajasagi et al. (Rajasagi et al., 2014). Pysam was used to determine RNA-seq read support of missense mutations. Eight-, Nine-, Ten-, and eleven-mer peptides derived from 3 ORFs with all possible combinations of missense mutations that overlap the genomic location of peptide in the ENCODE reference transcript set were generated by the in-silico peptide generation pipeline. Peptide sequences with internal stop codon(s) were discarded by the in-silico peptide generation pipeline. The expression level of each peptide was inferred by the lowest missense mutation RNA-seq read support. HLA class I (HLA-A, HLA-B, HLA-C) type of each tumor sample was identified using PHLAT (Bai et al., 2014). Binding affinity of peptides to HLA molecules expressed by the tumor was predicted using NetMHCpan (version 2.8). Binding affinity of peptides to null alleles, alternatively expressed alleles, and alleles not supported by NetMHCpan were not predicted. Neoantigens were considered high-affinity for IC50 values of less than or equal to 50nM, moderate-affinity for IC50 values of less than or equal to 150nM, and weak-affinity neoantigen for IC50 values of less than or equal to 500nM. Peptides were filtered by their binding affinities (IC50 nM) to class I alleles in the tumor sample's HLA type and RNA expression level of the predicted source transcript(s). Peptides with an IC50 value of less than or equal to 500 nM for at least 1 class I allele and RNA read support of at least 2 reads were considered predicted neoantigens.

Tumor Map

The Tumor Map is a published method (Newton et al., 2017) and represents a dimensionality reduction and visualization method for high dimensional genomic data (Ceccarelli et al., 2016; Farshidfar et al., 2017). It allows viewing and browsing relationships between high dimensional heterogeneous genomic samples in a two-dimensional map, analogous to exploring geographical maps in the Google Maps web application. Samples are arranged in a two-dimensional space and then associated to hexagons in a regular hexagonal grid. The relative distances in the map approximate the relative similarities between the samples in the original high-dimensional genomic space. Samples with similar genomic profiles should be placed near each other in the map. Samples that are less similar should be farther away from each other. Given such relations are preserved, clusters of samples that appear as "islands" in the map will indicate groups of samples that share genomic and/or epigenomic events.

To build multi-platform map, we combined tumor mRNA expression, copy number variation, and methylation profiles for Testicular Germ Cell Tumors (TGCT). We used mRNA expression data from RNA-seq (n = 137), copy number Gistic calls (n = 137), and methylation profiles from HumanMethylation450 (HM450) platform (n = 137). We computed sample-by-sample pair-wise similarities for each platform, producing three square similarity matrices. We used Spearman rank correlation (Spearman, 1904) as a similarity measure on these continuous-valued datasets (mRNA expression, copy number variation, and methylation). Next, we standardized each similarity matrix using the Context Likelihood of Relatedness (CLR) approach (Faith et al., 2007).Given a similarity matrix as input, this method outputs a set of *relative similarities*, where each similarity pair reflects how similar the two samples are to each other compared to how similar each is to *any sample* in a particular cohort. More formally, we compute the standardized similarity measure $Z_D(i,j)$ between two samples *i* and *j* from the dth dataset:

$$Z_d(i,j) = \frac{1}{2}\left(\frac{S_d(i,j) - m_d(i)}{\sqrt{v_d(i)}} + \frac{S_d(i,j) - m_d(j)}{\sqrt{v_d(j)}} \right),$$

where $m_d(k)$ is the mean and $v_d(k)$ is the variance of the similarities to a particular sample *k* in dataset d. We compute integrated similarity measures Z^* from the relative *Z*-scores across *D* different data platforms as:

$$Z^*(i,j) = \frac{\sum_{d=1}^{D} I(d,i) \cdot I(d,j) \cdot Z_d(i,j)}{\sum_{d=1}^{D} I(d,i) \cdot I(d,j)},$$

where $I(d,k)$ records whether sample *k* has data in dataset *d*. Thus, Z^* represents a simple averaging of the relative similarities between two samples across only those datasets for which both of the samples have valid (non-missing) observations. We note that Z^* could incorporate weightings for each dataset w_d indicating the importance of each of the *D* platforms being combined. However, we did not explore this option for the analysis presented here. The resulting standardized similarity matrix is a square samples-by-samples matrix where the samples are the union of all samples represented in the platforms.

To build the map layout, the closest neighborhood of 10 samples was selected for each sample from the standardized integrated similarity matrix. We represent the local neighborhoods as a graph where the nodes are the samples and an edge links any two samples if one of them is in the top 10 neighbors of the other. The magnitude of the similarity was used as the edge weight. An X-Y position in the two-dimensional plane was calculated from the graph using a spring-embedded graph layout (Golbeck and Mutton, 2005) algorithm implemented in the Distributed Recursive Graph Layout toolbox (Martin et al., 2011).The spring-embedded layout algorithm treats edges as springs and allows the springs to oscillate for a fixed amount of time with the energy inversely proportional to the edge weights. Under these conditions, springs with large weights do not oscillate much, causing those vertices to stay together. However, springs with small weights oscillate more and end up farther away from each other. This method allows constructing a two-dimensional spatial layout of the graph with clusters of samples forming clique-like hub sub-structures. Our method then associates each of the nodes with a fixed location a two-dimensional hexagonal grid. Each hexagon-shaped cell in the grid can be assigned no more than one vertex, and some can be assigned none representing “empty space” in the map. If multiple vertices contest for the same grid cell, a random vertex selection is made and placed into the cell; and the other competing vertices are assigned to neighboring empty cells using a greedy strategy, snapping around the original cell in a spiral-like manner.

PARADIGM Analysis

We applied PARADIGM (Vaske et al., 2010) method to the TGCT RNASeq mRNA expression (n=137) and copy number Gistic calls (n=137) data. PARADIGM is a computational inference method for activity levels of molecular entities (e.g. gene products, complexes, etc.) in the cell based on the mRNA levels, copy number, and molecular pathway relationships information. PARADIGM output for this TGCT cohort consists of 21,545 inferred pathway levels (IPLs). We restricted the IPLs to only those IPLs that had more than 3% of samples in the cohort with significant levels over the randomized background model, resulting in 7,617 significant IPLs.

For unsupervised analysis of TGCT cohort based on PARADIGM IPLs, we selected the top 3,000 most varying IPLs and performed unsupervised consensus k-means clustering, using complete linkage. We identified, using silhouette method, that the best solution contains three (k=3) clusters. These clusters are driven by tumor histology, consisting of seminoma tumors, embryonal non-seminoma tumors, and other non-seminoma tumors respectively.

For IPL enrichment analysis, we further restricted the 7,617 IPLs to only protein-coding IPLs, resulting in 1,477 IPLs. We ordered the samples (n=137) in the cohort by the histology groups and applied k-means clustering to the final set of IPLs, resulting in a solution of seven (k=7) major clusters. We annotated these clusters by performing gene set enrichment analysis on the IPLs in each cluster using MSigDB (Liberzon et al., 2011)molecular pathway database.

Supplemental References

Adzhubei, I., Jordan, D. M., and Sunyaev, S. R. (2013). Predicting functional effect of human missense mutations using PolyPhen-2. Curr Protoc Hum Genet *Chapter 7*, Unit7 20.

Akbani, R., Ng, P. K. S., Werner, H. M., Shahmoradgoli, M., Zhang, F., Ju, Z., Liu, W., Yang, J.-Y., Yoshihara, K., and Li, J. (2014). A pan-cancer proteomic perspective on The Cancer Genome Atlas. Nature communications *5*.

Almeida, L. G., Sakabe, N. J., Silva, M. C. C., Mundstein, A. S., Cohen, T., Chen, Y.-T., Chua, R., Gurung, S., Gnjatic, S., and Jungbluth, A. A. (2009). CTdatabase: a knowledge-base of high-throughput and curated data on cancer-testis antigens. Nucleic acids research *37*, D816-D819.

Bai, Y., Ni, M., Cooper, B., Wei, Y., and Fury, W. (2014). Inference of high resolution HLA types using genome-wide RNA or DNA sequencing reads. BMC genomics *15*, 325.

Bainbridge, M. N., Wang, M., Wu, Y., Newsham, I., Muzny, D. M., Jefferies, J. L., Albert, T. J., Burgess, D. L., and Gibbs, R. A. (2011). Targeted enrichment beyond the consensus coding DNA sequence exome reveals exons with higher variant densities. Genome Biol *12*, R68.

Bindea, G., Mlecnik, B., Tosolini, M., Kirilovsky, A., Waldner, M., Obenauf, A. C., Angell, H., Fredriksen, T., Lafontaine, L., and Berger, A. (2013). Spatiotemporal dynamics of intratumoral immune cells reveal the immune landscape in human cancer. Immunity *39*, 782-795.

Bolotin, D. A., Poslavsky, S., Mitrophanov, I., Shugay, M., Mamedov, I. Z., Putintseva, E. V., and Chudakov, D. M. (2015). MiXCR: software for comprehensive adaptive immunity profiling. Nature methods *12*, 380-381.

Bolotin, D. A., Shugay, M., Mamedov, I. Z., Putintseva, E. V., Turchaninova, M. A., Zvyagin, I. V., Britanova, O. V., and Chudakov, D. M. (2013). MiTCR: software for T-cell receptor sequencing data analysis. Nature methods *10*, 813-814.

Campan, M., Weisenberger, D. J., Trinh, B., and Laird, P. W. (2009). MethyLight. Methods Mol Biol *507*, 325-337.

Carter, H., Douville, C., Stenson, P. D., Cooper, D. N., and Karchin, R. (2013). Identifying Mendelian disease genes with the variant effect scoring tool. BMC Genomics *14 Suppl 3*, S3.

Carter, S. L., Cibulskis, K., Helman, E., McKenna, A., Shen, H., Zack, T., Laird, P. W., Onofrio, R. C., Winckler, W., and Weir, B. A. (2012). Absolute quantification of somatic DNA alterations in human cancer. Nature biotechnology *30*, 413-421.

Ceccarelli, M., Barthel, F. P., Malta, T. M., Sabedot, T. S., Salama, S. R., Murray, B. A., Morozova, O., Newton, Y., Radenbaugh, A., Pagnotta, S. M., *et al.* (2016). Molecular Profiling Reveals Biologically Discrete Subsets and Pathways of Progression in Diffuse Glioma. Cell *164*, 550-563.

Choi, Y., Sims, G. E., Murphy, S., Miller, J. R., and Chan, A. P. (2012). Predicting the functional effect of amino acid substitutions and indels. PLoS One *7*, e46688.

Chu, A., Robertson, G., Brooks, D., Mungall, A. J., Birol, I., Coope, R., Ma, Y., Jones, S., and Marra, M. A. (2015). Large-scale profiling of microRNAs for The Cancer Genome Atlas. Nucleic Acids Res.

Chun, S., and Fay, J. C. (2009). Identification of deleterious mutations within three human genomes. Genome Res *19*, 1553-1561.

Cingolani, P., Platts, A., Wang, L. L., Coon, M., Nguyen, T., Wang, L., Land, S. J., Lu, X., and Ruden, D. M. (2012). A program for annotating and predicting the effects of single nucleotide polymorphisms, SnpEff: SNPs in the genome of Drosophila melanogaster strain w1118; iso-2; iso-3. Fly *6*, 80-92.

Coombes, K., Neeley, E., and Joy, C. (2009). SuperCurve: SuperCurve Package. R package version *1*.

Court, F., Tayama, C., Romanelli, V., Martin-Trujillo, A., Iglesias-Platas, I., Okamura, K., Sugahara, N., Simon, C., Moore, H., Harness, J. V., *et al.* (2014). Genome-wide parent-of-origin DNA methylation analysis reveals the intricacies of human imprinting and suggests a germline methylation-independent mechanism of establishment. Genome research *24*, 554-569.

Davis, C. F., Ricketts, C. J., Wang, M., Yang, L., Cherniack, A. D., Shen, H., Buhay, C., Kang, H., Kim, S. C., Fahey, C. C., *et al.* (2014). The somatic genomic landscape of chromophobe renal cell carcinoma. Cancer Cell *26*, 319-330.

Davydov, E. V., Goode, D. L., Sirota, M., Cooper, G. M., Sidow, A., and Batzoglou, S. (2010). Identifying a high fraction of the human genome to be under selective constraint using GERP++. PLoS computational biology *6*, e1001025.

de Hoon, M. J., Imoto, S., Nolan, J., and Miyano, S. (2004). Open source clustering software. Bioinformatics *20*, 1453-1454.

DePristo, M. A., Banks, E., Poplin, R., Garimella, K. V., Maguire, J. R., Hartl, C., Philippakis, A. A., del Angel, G., Rivas, M. A., Hanna, M., *et al.* (2011). A framework for variation discovery and genotyping using next-generation DNA sequencing data. Nat Genet *43*, 491-498.

Dong, C., Wei, P., Jian, X., Gibbs, R., Boerwinkle, E., Wang, K., and Liu, X. (2015). Comparison and integration of deleteriousness prediction methods for nonsynonymous SNVs in whole exome sequencing studies. Hum Mol Genet *24*, 2125-2137.

Faith, J. J., Hayete, B., Thaden, J. T., Mogno, I., Wierzbowski, J., Cottarel, G., Kasif, S., Collins, J. J., and Gardner, T. S. (2007). Large-scale mapping and validation of Escherichia coli transcriptional regulation from a compendium of expression profiles. PLoS biol *5*, e8.

Fan, C., Prat, A., Parker, J. S., Liu, Y., Carey, L. A., Troester, M. A., and Perou, C. M. (2011). Building prognostic models for breast cancer patients using clinical variables and hundreds of gene expression signatures. BMC medical genomics *4*, 1.

Farshidfar, F., Zheng, S., Gingras, M. C., Newton, Y., Shih, J., Robertson, A. G., Hinoue, T., Hoadley, K. A., Gibb, E. A., Roszik, J., *et al.* (2017). Integrative Genomic Analysis of Cholangiocarcinoma Identifies Distinct IDH-Mutant Molecular Profiles. Cell reports *18*, 2780-2794.

Forbes, S. A., Tang, G., Bindal, N., Bamford, S., Dawson, E., Cole, C., Kok, C. Y., Jia, M., Ewing, R., and Menzies, A. (2009). COSMIC (the Catalogue of Somatic Mutations in Cancer): a resource to investigate acquired mutations in human cancer. Nucleic acids research, gkp995.
Frigyesi, A., Gisselsson, D., Hansen, G. B., Soller, M., Mitelman, F., and Höglund, M. (2004). A model for karyotypic evolution in testicular germ cell tumors. Genes, Chromosomes and Cancer *40*, 172-178.
Gatza, M. L., Lucas, J. E., Barry, W. T., Kim, J. W., Wang, Q., Crawford, M. D., Datto, M. B., Kelley, M., Mathey-Prevot, B., and Potti, A. (2010). A pathway-based classification of human breast cancer. Proceedings of the National Academy of Sciences *107*, 6994-6999.
Ghassabeh, G. H., De Baetselier, P., Brys, L., Noël, W., Van Ginderachter, J. A., Meerschaut, S., Beschin, A., Brombacher, F., and Raes, G. (2006). Identification of a common gene signature for type II cytokine–associated myeloid cells elicited in vivo in different pathologic conditions. Blood *108*, 575-583.
Golbeck, J., and Mutton, P. (2005). Spring-Embedded graphs for semantic visualization. Visualizing the Semantic Web, 172-182.
Hastie, T., Tibshirani, R., Sherlock, G., Eisen, M., Brown, P., and Botstein, D. (1999). Imputing missing data for gene expression arrays. In, (Stanford University Statistics Department Technical report).
Hennessy, B. T., Lu, Y., Gonzalez-Angulo, A. M., Carey, M. S., Myhre, S., Ju, Z., Davies, M. A., Liu, W., Coombes, K., and Meric-Bernstam, F. (2010). A technical assessment of the utility of reverse phase protein arrays for the study of the functional proteome in non-microdissected human breast cancers. Clinical proteomics *6*, 129.
Hennessy, B. T., Lu, Y., Poradosu, E., Yu, Q., Yu, S., Hall, H., Carey, M. S., Ravoori, M., Gonzalez-Angulo, A. M., and Birch, R. (2007). Pharmacodynamic markers of perifosine efficacy. Clinical cancer research *13*, 7421-7431.
Hollern, D. P., and Andrechek, E. R. (2014). A genomic analysis of mouse models of breast cancer reveals molecular features of mouse models and relationships to human breast cancer. Breast Cancer Res *16*.
Hu, J., He, X., Baggerly, K. A., Coombes, K. R., Hennessy, B. T., and Mills, G. B. (2007). Non-parametric quantification of protein lysate arrays. Bioinformatics *23*, 1986-1994.
Iglesia, M. D., Vincent, B. G., Parker, J. S., Hoadley, K. A., Carey, L. A., Perou, C. M., and Serody, J. S. (2014). Prognostic B-cell signatures using mRNA-seq in patients with subtype-specific breast and ovarian cancer. Clinical Cancer Research *20*, 3818-3829.
Kircher, M., Witten, D. M., Jain, P., O'Roak, B. J., Cooper, G. M., and Shendure, J. (2014). A general framework for estimating the relative pathogenicity of human genetic variants. Nat Genet *46*, 310-315.
Korn, J. M., Kuruvilla, F. G., McCarroll, S. A., Wysoker, A., Nemesh, J., Cawley, S., Hubbell, E., Veitch, J., Collins, P. J., and Darvishi, K. (2008). Integrated genotype calling and association analysis of SNPs, common copy number polymorphisms and rare CNVs. Nature genetics *40*, 1253-1260.
Kumar, P., Henikoff, S., and Ng, P. C. (2009). Predicting the effects of coding non-synonymous variants on protein function using the SIFT algorithm. Nat Protoc *4*, 1073-1081.
Lawrence, M. S., Stojanov, P., Polak, P., Kryukov, G. V., Cibulskis, K., Sivachenko, A., Carter, S. L., Stewart, C., Mermel, C. H., Roberts, S. A., *et al.* (2013). Mutational heterogeneity in cancer and the search for new cancer-associated genes. Nature *499*, 214-218.
Li, B., and Dewey, C. N. (2011). RSEM: accurate transcript quantification from RNA-Seq data with or without a reference genome. BMC bioinformatics *12*, 323.
Li, H., and Durbin, R. (2009). Fast and accurate short read alignment with Burrows-Wheeler transform. Bioinformatics *25*, 1754-1760.
Li, H., Handsaker, B., Wysoker, A., Fennell, T., Ruan, J., Homer, N., Marth, G., Abecasis, G., Durbin, R., and Genome Project Data Processing, S. (2009). The Sequence Alignment/Map format and SAMtools. Bioinformatics *25*, 2078-2079.
Li, J., and Tibshirani, R. (2013). Finding consistent patterns: a nonparametric approach for identifying differential expression in RNA-Seq data. Stat Methods Med Res *22*, 519-536.
Liang, J., Shao, S. H., Xu, Z.-X., Hennessy, B., Ding, Z., Larrea, M., Kondo, S., Dumont, D. J., Gutterman, J. U., and Walker, C. L. (2007). The energy sensing LKB1–AMPK pathway regulates p27kip1 phosphorylation mediating the decision to enter autophagy or apoptosis. Nature cell biology *9*, 218-224.
Liberzon, A., Subramanian, A., Pinchback, R., Thorvaldsdottir, H., Tamayo, P., and Mesirov, J. P. (2011). Molecular signatures database (MSigDB) 3.0. Bioinformatics *27*, 1739-1740.
Liu, X., Wu, C., Li, C., and Boerwinkle, E. (2016). dbNSFP v3.0: A One-Stop Database of Functional Predictions and Annotations for Human Nonsynonymous and Splice-Site SNVs. Hum Mutat *37*, 235-241.
Martin, S., Brown, W. M., Klavans, R., and Boyack, K. W. (2011). OpenOrd: an open-source toolbox for large graph layout. Paper presented at: IS&T/SPIE Electronic Imaging (International Society for Optics and Photonics).
McCarroll, S. A., Kuruvilla, F. G., Korn, J. M., Cawley, S., Nemesh, J., Wysoker, A., Shapero, M. H., de Bakker, P. I., Maller, J. B., and Kirby, A. (2008). Integrated detection and population-genetic analysis of SNPs and copy number variation. Nature genetics *40*, 1166-1174.
Mermel, C. H., Schumacher, S. E., Hill, B., Meyerson, M. L., Beroukhim, R., and Getz, G. (2011). GISTIC2. 0 facilitates sensitive and confident localization of the targets of focal somatic copy-number alteration in human cancers. Genome biology *12*, 1.
Monti, S., Tamayo, P., Mesirov, J., and Golub, T. (2003). Consensus clustering: a resampling-based method for class discovery and visualization of gene expression microarray data. Machine learning *52*, 91-118.
Mose, L. E., Selitsky, S. R., Bixby, L. M., Marron, D. L., Iglesia, M. D., Serody, J. S., Perou, C. M., Vincent, B. G., and Parker, J. S. (2016). Assembly-based inference of B-cell receptor repertoires from short read RNA sequencing data with V'DJer. Bioinformatics, btw526.

Network, C. G. A. R. (2011). Integrated genomic analyses of ovarian carcinoma. Nature *474*, 609-615.
Newton, Y., Novak, A. M., Swatloski, T., McColl, D. C., Chopra, S., Graim, K., Weinstein, A. S., Baertsch, R., Salama, S. R., and Ellrott, K. (2017). TumorMap: Exploring the Molecular Similarities of Cancer Samples in an Interactive Portal. Cancer research *77*, e111-e114.
Olshen, A. B., Venkatraman, E., Lucito, R., and Wigler, M. (2004). Circular binary segmentation for the analysis of array-based DNA copy number data. Biostatistics *5*, 557-572.
Quang, D., Chen, Y., and Xie, X. (2015). DANN: a deep learning approach for annotating the pathogenicity of genetic variants. Bioinformatics *31*, 761-763.
Radenbaugh, A. J., Ma, S., Ewing, A., Stuart, J. M., Collisson, E. A., Zhu, J., and Haussler, D. (2014). RADIA: RNA and DNA integrated analysis for somatic mutation detection. PloS one *9*, e111516.
Rajasagi, M., Shukla, S. A., Fritsch, E. F., Keskin, D. B., DeLuca, D., Carmona, E., Zhang, W., Sougnez, C., Cibulskis, K., Sidney, J., *et al.* (2014). Systematic identification of personal tumor-specific neoantigens in chronic lymphocytic leukemia. Blood *124*, 453-462.
Reid, J. G., Carroll, A., Veeraraghavan, N., Dahdouli, M., Sundquist, A., English, A., Bainbridge, M., White, S., Salerno, W., Buhay, C., *et al.* (2014). Launching genomics into the cloud: deployment of Mercury, a next generation sequence analysis pipeline. BMC Bioinformatics *15*, 30.
Reva, B., Antipin, Y., and Sander, C. (2011). Predicting the functional impact of protein mutations: application to cancer genomics. Nucleic Acids Res *39*, e118.
Rody, A., Holtrich, U., Pusztai, L., Liedtke, C., Gaetje, R., Ruckhaeberle, E., Solbach, C., Hanker, L., Ahr, A., and Metzler, D. (2009). T-cell metagene predicts a favorable prognosis in estrogen receptor-negative and HER2-positive breast cancers. Breast Cancer Research *11*, 1.
Rousseeuw, P. J. (1987). Silhouettes: a graphical aid to the interpretation and validation of cluster analysis. Journal of computational and applied mathematics *20*, 53-65.
Saunders, C. T., Wong, W. S., Swamy, S., Becq, J., Murray, L. J., and Cheetham, R. K. (2012). Strelka: accurate somatic small-variant calling from sequenced tumor–normal sample pairs. Bioinformatics *28*, 1811-1817.
Schwarz, J. M., Cooper, D. N., Schuelke, M., and Seelow, D. (2014). MutationTaster2: mutation prediction for the deep-sequencing age. Nat Methods *11*, 361-362.
Shihab, H. A., Rogers, M. F., Gough, J., Mort, M., Cooper, D. N., Day, I. N., Gaunt, T. R., and Campbell, C. (2015). An integrative approach to predicting the functional effects of non-coding and coding sequence variation. Bioinformatics *31*, 1536-1543.
Smigielski, E. M., Sirotkin, K., Ward, M., and Sherry, S. T. (2000). dbSNP: a database of single nucleotide polymorphisms. Nucleic acids research *28*, 352-355.
Spearman, C. (1904). The proof and measurement of association between two things. The American journal of psychology *15*, 72-101.
Tibes, R., Qiu, Y., Lu, Y., Hennessy, B., Andreeff, M., Mills, G. B., and Kornblau, S. M. (2006). Reverse phase protein array: validation of a novel proteomic technology and utility for analysis of primary leukemia specimens and hematopoietic stem cells. Molecular cancer therapeutics *5*, 2512-2521.
Triche, T. J., Jr., Weisenberger, D. J., Van Den Berg, D., Laird, P. W., and Siegmund, K. D. (2013). Low-level processing of Illumina Infinium DNA Methylation BeadArrays. Nucleic Acids Res *41*, e90.
Vaske, C. J., Benz, S. C., Sanborn, J. Z., Earl, D., Szeto, C., Zhu, J., Haussler, D., and Stuart, J. M. (2010). Inference of patient-specific pathway activities from multi-dimensional cancer genomics data using PARADIGM. Bioinformatics *26*, i237-i245.
Wang, K., Singh, D., Zeng, Z., Coleman, S. J., Huang, Y., Savich, G. L., He, X., Mieczkowski, P., Grimm, S. A., Perou, C. M., *et al.* (2010). MapSplice: accurate mapping of RNA-seq reads for splice junction discovery. Nucleic acids research *38*, e178.
Wang, L., Ni, X., Covington, K. R., Yang, B. Y., Shiu, J., Zhang, X., Xi, L., Meng, Q., Langridge, T., Drummond, J., *et al.* (2015). Genomic profiling of Sezary syndrome identifies alterations of key T cell signaling and differentiation genes. Nat Genet *47*, 1426-1434.
Wilkerson, M. D., and Hayes, D. N. (2010). ConsensusClusterPlus: a class discovery tool with confidence assessments and item tracking. Bioinformatics *26*, 1572-1573.
Wilks, C., Cline, M. S., Weiler, E., Diehkans, M., Craft, B., Martin, C., Murphy, D., Pierce, H., Black, J., Nelson, D., *et al.* (2014). The Cancer Genomics Hub (CGHub): overcoming cancer through the power of torrential data. Database (Oxford) *2014*.
Zack, T. I., Schumacher, S. E., Carter, S. L., Cherniack, A. D., Saksena, G., Tabak, B., Lawrence, M. S., Zhang, C.-Z., Wala, J., and Mermel, C. H. (2013). Pan-cancer patterns of somatic copy number alteration. Nature genetics *45*, 1134-1140.
